# Studies in Hawaiian Diptera II: New Distributional Records for Endemic *Scatella* (Ephydridae)

**DOI:** 10.3897/BDJ.2.e1110

**Published:** 2014-08-18

**Authors:** Patrick M O'Grady, Keith Arakaki, Neal L Evenhuis

**Affiliations:** † UC Berkeley, Berkeley, United States of America; ‡ Bernice Pauahi Bishop Museum, Honolulu, Hawai‘i 96817-2704, United States of America

**Keywords:** Hawaiian Islands, Diptera, Ephydridae, *
Scatella
*, distributions, new island records

## Abstract

Here we summarize the known distributional data for the Hawaiian *Scatella* (Ephydridae). We report on four new island records; *Scatella amnica* and *Scatella stagnalis* from Kauai, *Scatella oahuense* from Lanai, and *Scatella terryi* from Maui. A list of material present, comprising over 3100 individual specimen records in the collections of the Bernice Pauahi Bishop Museum, University of Hawaii at Manoa, and Essig Musuem of Entomology at UC Berkeley is included, along with details distributional maps for the Hawaiian endemic species.

## Introduction

Diptera research in Hawaii dates to the latter part of the 18th century when RCL Perkins was employed to survey the terrestrial biota of the Hawaiian Archipelago (Evenhuis 2007). Subsequent research by Sweezy, Bryan, Williams, Cresson, Wirth, Hardy and others greatly expanded the number of endemic species and refined our understanding of their relationships (reviewed in Zimmerman and Liebherr 2001). The senior author's laboratory has been collecting Hawaiian Diptera for the past 15 years in order to elucidate the patterns and processes involved in the impressive radiations seen in these lineages in the Hawaiian Islands. We have amassed a large amount of fine-scale distributional data and new island records for many species and this is the second in a series of papers on the taxonomy and distribution of endemic Hawaiian Diptera. The current paper reports four new island records from the genus *Scatella* (Diptera: Ephydridae) and provides a comprehensive catalog of the available material in the Bernice Pauahi Bishop Museum (BPBM), University of Hawaii at Manoa (UHM), and Essig Musuem of Entomology at UC Berkeley (EMEC).

Ephydridae, or the shore flies, is a cosmopolitan family of ecologically diverse acalyptrate Diptera, with 2000 species placed in five subfamilies (Mathis and Zatwarnicki 1995, Pape et al. 2011). Most ephydrids are aquatic or semi-aquatic, but some genera have adapted to harsh environments and can survive in high elevations, sulfurous hot springs, highly alkaline or saline lakes, and even in pools of crude petroleum (Evenhuis 2008, Foote 1995). Many ephydrids are beneficial and provide food for wildlife, such as migratory birds (Wirth et al. 1987). Others are agricultural pests, mining in the stems or leaves of watercress, rice, barley, and other irrigated cereals (Grigarick 1959). The majority of native Hawaiian ephydrids are placed in the cosmopolitan genus *Scatella*, a group containing a total of 135 species (Brown 2010). *Scatella* is widespread in the Pacific basin and it has been recorded from Hawaii, French Polynesia, Western Samoa, Australia, New Zealand, and Easter Island (http://hbs.bishopmuseum.org/aocat/aocathome.html). There are 17 Hawaiian species (Table 1), 15 of which are endemic (Nishida 2002). *Scatella* has a complex taxonomic history, particularly in Hawaii. Tenorio (1980) classified these taxa in three separate genera (*Scatella*, *Neoscatella*, and *Apulvillus*), but they have since been combined into the same genus. Ten species are found on multiple islands and only five (*Scatellacinereifacies*, *Scatellakauaiensis*, *Scatellawilliamsi*, *Scatellawirthi*, and *Scatellafemoralis*) are single island endemics (Nishida 2002).

## Materials and methods

### Literature and specimen review

We have reviewed the literature for the Hawaiian *Scatella* and have attempted to include infomation on both the taxonomic history of the species and all occurrences of the endemic taxa in the literature, including the original descriptions, subsequent revisions, additional descriptive notes, range expansions and new island records and catalogs. We list the primary type, paratypes and other material examined. We include all records from the EMEC and the BPBM. Additional material deposited in the National Museum of Natural History, Smithsonian Institution (USNM) and the UHM are also presented here. All museum abbreviations conform to Evenhuis (2014).

### Collection methods

All material was obtained from general sweeping of vegetation, seeps and streams. Samples were preserved in 95% ethanol (ETOH) and transported to UC Berkeley for identification and subsequent molecular work. The key and descriptions in Tenorio (1980) were used to identify the Hawaiian *Scatella*. Vouchers for all species were preserved in 95% ETOH and are deposited in the BPBM and EMEC.

### Species distribution maps

Distribution maps for each species were produced by combining literature records and the senior author's collections during the past 15 years. Historical collections, shown in red, were georeferenced based on known place names and collection localities. These localities may not be exact but will give the reader some notion of the distribution of these taxa. Placemarks in the .kml file (Suppl. materials 1, 2, 3, 4, 5, 6, 7, 8, 9, 10, 11, 12, 13, 14, 15) are named by the collector and the year of collection. Two widespread species, *Scatella sexnotata* and *Scatella stagnalis*, were not mapped. These taxa are very common in agricultural settings (irrigation ditches, greenhouses, etc.) and in aquatic habitats close to human habitation. More recent collections, shown in white, were georeferenced using a Garmin Oregon 450 GPS unit.

## Checklists

### Checklist of *Scatella* species found in the Hawaiian Islands

#### Scatella amnica

(Tenorio, 1980)

##### Materials

- **Type status:** Holotype. **Occurrence:** catalogNumber: 9476; recordedBy: JA Tenorio; individualCount: 1; sex: male; lifeStage: adult; **Location:** islandGroup: Hawaiian Islands; island: Hawaii; country: United States; stateProvince: Hawaii; county: Hawaii; verbatimLocality: Wailuku River; verbatimElevation: 2170 ft.; **Identification:** identifiedBy: JA Tenorio; **Event:** verbatimEventDate: 14.viii.1970; **Record Level:** institutionCode: BPBM
- **Type status:** Paratype. **Occurrence:** recordedBy: WW Wirth; individualCount: 1; lifeStage: adult; **Location:** islandGroup: Hawaiian Islands; island: Hawaii; country: United States; stateProvince: Hawaii; county: Hawaii; verbatimLocality: Akaka Falls, Hilo; **Identification:** identifiedBy: JA Tenorio; **Event:** verbatimEventDate: iii.1946; **Record Level:** institutionCode: UHM
- **Type status:** Paratype. **Occurrence:** recordedBy: DE Hardy; individualCount: 1; lifeStage: adult; **Location:** islandGroup: Hawaiian Islands; island: Molokai; country: United States; stateProvince: Hawaii; county: Maui; verbatimLocality: Halawa Valley; **Identification:** identifiedBy: JA Tenorio; **Event:** verbatimEventDate: vi.1952; **Record Level:** institutionCode: UHM
- **Type status:** Paratype. **Occurrence:** recordedBy: WC Mitchel; individualCount: 1; lifeStage: adult; **Location:** islandGroup: Hawaiian Islands; island: Hawaii; country: United States; stateProvince: Hawaii; county: Hawaii; verbatimLocality: Paauilo; **Identification:** identifiedBy: JA Tenorio; **Event:** verbatimEventDate: viii.1952; **Record Level:** institutionCode: UHM
- **Type status:** Paratype. **Occurrence:** recordedBy: DE Hardy; individualCount: 1; lifeStage: adult; **Location:** islandGroup: Hawaiian Islands; island: Maui; country: United States; stateProvince: Hawaii; county: Maui; verbatimLocality: Wailua; **Identification:** identifiedBy: JA Tenorio; **Event:** verbatimEventDate: vii.1953; **Record Level:** institutionCode: UHM
- **Type status:** Paratype. **Occurrence:** recordedBy: DE Hardy, R Namba; individualCount: 1; lifeStage: adult; **Location:** islandGroup: Hawaiian Islands; island: Maui; country: United States; stateProvince: Hawaii; county: Maui; verbatimLocality: Waikamoi; verbatimElevation: 4000 ft.; **Identification:** identifiedBy: JA Tenorio; **Event:** verbatimEventDate: vi.1956; **Record Level:** institutionCode: UHM
- **Type status:** Paratype. **Occurrence:** recordedBy: R. Namba; individualCount: 1; lifeStage: adult; **Location:** islandGroup: Hawaiian Islands; island: Maui; country: United States; stateProvince: Hawaii; county: Maui; verbatimLocality: Kula Pipeline; verbatimElevation: 4200 ft.; **Identification:** identifiedBy: JA Tenorio; **Event:** verbatimEventDate: vii.1958; **Record Level:** institutionCode: UHM
- **Type status:** Paratype. **Occurrence:** recordedBy: DE Hardy; individualCount: 1; lifeStage: adult; **Location:** islandGroup: Hawaiian Islands; island: Hawaii; country: United States; stateProvince: Hawaii; county: Hawaii; verbatimLocality: Akaka Falls; **Identification:** identifiedBy: JA Tenorio; **Event:** verbatimEventDate: 19.iv.1964; **Record Level:** institutionCode: UHM
- **Type status:** Paratype. **Occurrence:** recordedBy: DE Hardy; individualCount: 1; lifeStage: adult; **Location:** islandGroup: Hawaiian Islands; island: Molokai; country: United States; stateProvince: Hawaii; county: Maui; verbatimLocality: Waiahanau Valley; **Identification:** identifiedBy: JA Tenorio; **Event:** verbatimEventDate: 17.xi.1964; **Record Level:** institutionCode: UHM
- **Type status:** Paratype. **Occurrence:** recordedBy: TW Fisher; individualCount: 1; lifeStage: adult; **Location:** islandGroup: Hawaiian Islands; island: Maui; country: United States; stateProvince: Hawaii; county: Maui; verbatimLocality: 5 miles east of Kaeleku; **Identification:** identifiedBy: JA Tenorio; **Event:** verbatimEventDate: 8.iv.1965; **Record Level:** institutionCode: UHM
- **Type status:** Paratype. **Occurrence:** recordedBy: JA Tenorio; individualCount: 1; lifeStage: adult; **Location:** islandGroup: Hawaiian Islands; island: Hawaii; country: United States; stateProvince: Hawaii; county: Hawaii; verbatimLocality: Piihonua, Wailuku Stream; **Identification:** identifiedBy: JA Tenorio; **Event:** verbatimEventDate: 27.xii.1969; **Record Level:** institutionCode: UHM
- **Type status:** Paratype. **Occurrence:** recordedBy: DE Hardy; individualCount: 1; lifeStage: adult; **Location:** islandGroup: Hawaiian Islands; island: Maui; country: United States; stateProvince: Hawaii; county: Maui; verbatimLocality: Kipahulu Valley; **Identification:** identifiedBy: JA Tenorio; **Event:** verbatimEventDate: 21.ii.1970; **Record Level:** institutionCode: UHM
- **Type status:** Paratype. **Occurrence:** recordedBy: JA Tenorio; individualCount: 1; lifeStage: adult; **Location:** islandGroup: Hawaiian Islands; island: Hawaii; country: United States; stateProvince: Hawaii; county: Hawaii; verbatimLocality: Akaka Falls State Park; **Identification:** identifiedBy: JA Tenorio; **Event:** verbatimEventDate: 28.v.1970; **Record Level:** institutionCode: UHM
- **Type status:** Paratype. **Occurrence:** recordedBy: JA Tenorio; individualCount: 4; lifeStage: adult; **Location:** islandGroup: Hawaiian Islands; island: Hawaii; country: United States; stateProvince: Hawaii; county: Hawaii; verbatimLocality: Boiling Pots; **Identification:** identifiedBy: JA Tenorio; **Event:** verbatimEventDate: 28.v.1970; **Record Level:** institutionCode: BPBM
- **Type status:** Paratype. **Occurrence:** recordedBy: JA Tenorio; individualCount: 1; lifeStage: adult; **Location:** islandGroup: Hawaiian Islands; island: Hawaii; country: United States; stateProvince: Hawaii; county: Hawaii; verbatimLocality: Kawainui Stream; **Identification:** identifiedBy: JA Tenorio; **Event:** verbatimEventDate: 28.v.1970; **Record Level:** institutionCode: UHM
- **Type status:** Paratype. **Occurrence:** recordedBy: JA Tenorio; individualCount: 1; lifeStage: adult; **Location:** islandGroup: Hawaiian Islands; island: Hawaii; country: United States; stateProvince: Hawaii; county: Hawaii; verbatimLocality: Rainbow Falls; **Identification:** identifiedBy: JA Tenorio; **Event:** verbatimEventDate: 28.v.1970; **Record Level:** institutionCode: UHM
- **Type status:** Paratype. **Occurrence:** recordedBy: JA Tenorio; individualCount: 4; lifeStage: adult; **Location:** islandGroup: Hawaiian Islands; island: Hawaii; country: United States; stateProvince: Hawaii; county: Hawaii; verbatimLocality: Wailuku River; verbatimElevation: 2170 ft.; **Identification:** identifiedBy: JA Tenorio; **Event:** verbatimEventDate: 14.viii.1970; **Record Level:** institutionCode: BPBM
- **Type status:** Paratype. **Occurrence:** catalogNumber: 9476a; recordedBy: JA Tenorio; individualCount: 1; sex: female; lifeStage: adult; **Location:** islandGroup: Hawaiian Islands; island: Hawaii; country: United States; stateProvince: Hawaii; county: Hawaii; verbatimLocality: Wailuku River; verbatimElevation: 2170 ft.; **Identification:** identifiedBy: JA Tenorio; **Event:** verbatimEventDate: 14.viii.1970; **Record Level:** institutionCode: BPBM
- **Type status:** Paratype. **Occurrence:** recordedBy: JA Tenorio; individualCount: 1; lifeStage: adult; **Location:** islandGroup: Hawaiian Islands; island: Maui; country: United States; stateProvince: Hawaii; county: Maui; verbatimLocality: Kopiliula Stream; verbatimElevation: 1200 ft.; **Identification:** identifiedBy: JA Tenorio; **Event:** verbatimEventDate: 4.ix.1970; **Record Level:** institutionCode: UHM
- **Type status:** Other material. **Occurrence:** catalogNumber: EMEC7356016; recordedBy: PM O'Grady, KN Magnacca, RT Lapoint, GM Bennett; individualCount: 5; lifeStage: adult; **Location:** islandGroup: Hawaiian Islands; island: Molokai; country: United States; stateProvince: Hawaii; county: Maui; verbatimLocality: Kamakou Forest Reserve, sweeping in stream before tunnel; verbatimLatitude: 21°6'42.78"N; verbatimLongitude: 156°54'25.20"W; geodeticDatum: WGS84; **Identification:** identifiedBy: PM O'Grady; **Event:** verbatimEventDate: 19.ii.2007; **Record Level:** institutionCode: EMEC; collectionCode: 379.2
- **Type status:** Other material. **Occurrence:** catalogNumber: EMEC7356017; recordedBy: PM O'Grady, NL Evenhuis, GM Bennett; individualCount: 3; lifeStage: adult; **Location:** islandGroup: Hawaiian Islands; island: Maui; country: United States; stateProvince: Hawaii; county: Maui; verbatimLocality: Hana Highway, first stop, sweeping stream; verbatimLatitude: 20°54'0.07"N; verbatimLongitude: 156°13'17.20"W; geodeticDatum: WGS85; **Identification:** identifiedBy: PM O'Grady; **Event:** verbatimEventDate: 21.xi.2009; **Record Level:** institutionCode: EMEC; collectionCode: 547.9
- **Type status:** Other material. **Occurrence:** catalogNumber: EMEC7356018; recordedBy: PM O'Grady, BS Ort, NA Pantoja; lifeStage: adult; **Location:** islandGroup: Hawaiian Islands; island: Hawaii; country: United States; stateProvince: Hawaii; county: Hawaii; verbatimLocality: Whittington Beach Park, South Coast, Kau, sweeping lava rocks and estuary; verbatimLatitude: 19°5'7.18"N; verbatimLongitude: 155°32'59.65"W; geodeticDatum: WGS84; **Identification:** identifiedBy: PM O'Grady; **Event:** verbatimEventDate: 1.viii.2010; **Record Level:** institutionCode: EMEC; collectionCode: 612.5
- **Type status:** Other material. **Occurrence:** catalogNumber: EMEC7356015; recordedBy: PM O'Grady, RT Lapoint, GM Bennett, BS Ort, NA Pantoja, E Owen; individualCount: 1; lifeStage: adult; **Location:** islandGroup: Hawaiian Islands; island: Kauai; country: United States; stateProvince: Hawaii; county: Kauai; verbatimLocality: Kohua Ridge Trail, Mohihi Stream, 0-1.0 miles, sweeping stream; verbatimLatitude: 22°6'44.61"N; verbatimLongitude: 159°36'21.69"W; geodeticDatum: WGS84; **Identification:** identifiedBy: PM O'Grady; **Event:** verbatimEventDate: 6.i.2010; **Record Level:** institutionCode: EMEC; collectionCode: 574.5

##### Ecological interactions

###### Native status

Endemic.

##### Distribution

Hawaiian Islands: Kauai, Molokai, Maui, Hawaii.

#### Scatella bryani

Cresson, 1926

##### Materials

- **Type status:** Holotype. **Occurrence:** catalogNumber: 354; recordedBy: EH Bryan Jr.; individualCount: 1; sex: male; lifeStage: adult; **Location:** islandGroup: Hawaiian Islands; island: Kauai; country: United States; stateProvince: Hawaii; county: Kauai; verbatimLocality: Awaawapuhi; **Event:** verbatimEventDate: 16.vi.1922; **Record Level:** institutionCode: BPBM
- **Type status:** Other material. **Occurrence:** recordedBy: RCL Perkins; individualCount: 1; lifeStage: adult; **Location:** islandGroup: Hawaiian Islands; island: Oahu; country: United States; stateProvince: Hawaii; county: Honolulu; verbatimLocality: Honolulu; **Identification:** identifiedBy: JA Tenorio; **Record Level:** institutionCode: BPBM
- **Type status:** Other material. **Occurrence:** recordedBy: EH Bryan Jr.; individualCount: 1; lifeStage: adult; **Location:** islandGroup: Hawaiian Islands; island: Oahu; country: United States; stateProvince: Hawaii; county: Honolulu; verbatimLocality: Waianae, Kolekole; **Event:** verbatimEventDate: 29.ii.1920; **Record Level:** institutionCode: BPBM
- **Type status:** Other material. **Occurrence:** recordedBy: EH Bryan Jr.; individualCount: 1; lifeStage: adult; **Location:** islandGroup: Hawaiian Islands; island: Oahu; country: United States; stateProvince: Hawaii; county: Honolulu; verbatimLocality: Waianae; **Event:** verbatimEventDate: 29.ii.1920; **Record Level:** institutionCode: BPBM
- **Type status:** Other material. **Occurrence:** recordedBy: EH Bryan Jr.; individualCount: 1; lifeStage: adult; **Location:** islandGroup: Hawaiian Islands; island: Oahu; country: United States; stateProvince: Hawaii; county: Honolulu; verbatimLocality: Kamehameha School Campus; **Event:** verbatimEventDate: ii.1923; **Record Level:** institutionCode: BPBM
- **Type status:** Other material. **Occurrence:** recordedBy: EH Bryan Jr.; individualCount: 2; lifeStage: adult; **Location:** islandGroup: Hawaiian Islands; island: Oahu; country: United States; stateProvince: Hawaii; county: Honolulu; verbatimLocality: Honolulu, Kalihi Valley, about pools of stagnant rainwater near the Bishop Museum; **Identification:** identifiedBy: ET Cresson; **Event:** verbatimEventDate: 12.ii.1923; **Record Level:** institutionCode: BPBM
- **Type status:** Other material. **Occurrence:** recordedBy: EH Bryan Jr.; individualCount: 1; lifeStage: adult; **Location:** islandGroup: Hawaiian Islands; island: Oahu; country: United States; stateProvince: Hawaii; county: Honolulu; verbatimLocality: Honolulu; **Identification:** identifiedBy: ET Cresson; **Event:** verbatimEventDate: 2.iv.1923; **Record Level:** institutionCode: BPBM
- **Type status:** Other material. **Occurrence:** recordedBy: O Bryant; individualCount: 1; lifeStage: adult; **Location:** islandGroup: Hawaiian Islands; island: Oahu; country: United States; stateProvince: Hawaii; county: Honolulu; verbatimLocality: Honolulu; **Event:** verbatimEventDate: 8.ii.1932; **Record Level:** institutionCode: BPBM
- **Type status:** Other material. **Occurrence:** recordedBy: RL Usinger; individualCount: 1; lifeStage: adult; **Location:** islandGroup: Hawaiian Islands; island: Hawaii; country: United States; stateProvince: Hawaii; county: Hawaii; verbatimLocality: Humuula; **Identification:** identifiedBy: JA Tenorio; **Event:** verbatimEventDate: 8.viii.1935; **Record Level:** institutionCode: BPBM
- **Type status:** Other material. **Occurrence:** recordedBy: Y Kondo; individualCount: 1; lifeStage: adult; **Location:** islandGroup: Hawaiian Islands; island: Oahu; country: United States; stateProvince: Hawaii; county: Honolulu; verbatimLocality: Honolulu; **Identification:** identifiedBy: JA Tenorio; **Event:** verbatimEventDate: 16.iv.1941; **Record Level:** institutionCode: BPBM
- **Type status:** Other material. **Occurrence:** recordedBy: WW Wirth; lifeStage: adult; **Location:** islandGroup: Hawaiian Islands; island: Oahu; country: United States; stateProvince: Hawaii; county: Honolulu; verbatimLocality: Hering Valley; **Event:** verbatimEventDate: 29.iv.1945; **Record Level:** institutionCode: BPBM
- **Type status:** Other material. **Occurrence:** catalogNumber: USNMENT 00972745; recordedBy: WW Wirth; individualCount: 1; sex: female; lifeStage: adult; **Location:** islandGroup: Hawaiian Islands; island: Oahu; country: United States; stateProvince: Hawaii; county: Honolulu; verbatimLocality: Kahuku, at light trap; **Event:** verbatimEventDate: 1.ii.1946; **Record Level:** institutionCode: USNM
- **Type status:** Other material. **Occurrence:** catalogNumber: USNMENT 00972746; recordedBy: WW Wirth; individualCount: 1; sex: female; lifeStage: adult; **Location:** islandGroup: Hawaiian Islands; island: Oahu; country: United States; stateProvince: Hawaii; county: Honolulu; verbatimLocality: Kahuku, at light trap; **Event:** verbatimEventDate: 1.ii.1946; **Record Level:** institutionCode: USNM
- **Type status:** Other material. **Occurrence:** catalogNumber: USNMENT 00972747; recordedBy: WW Wirth; individualCount: 1; sex: male; lifeStage: adult; **Location:** islandGroup: Hawaiian Islands; island: Oahu; country: United States; stateProvince: Hawaii; county: Honolulu; verbatimLocality: Kahuku, at light trap; **Event:** verbatimEventDate: 1.ii.1946; **Record Level:** institutionCode: USNM
- **Type status:** Other material. **Occurrence:** catalogNumber: USNMENT 00972748; recordedBy: WW Wirth; individualCount: 1; sex: male; lifeStage: adult; **Location:** islandGroup: Hawaiian Islands; island: Oahu; country: United States; stateProvince: Hawaii; county: Honolulu; verbatimLocality: Kahuku, at light trap; **Event:** verbatimEventDate: 1.ii.1946; **Record Level:** institutionCode: USNM
- **Type status:** Other material. **Occurrence:** catalogNumber: USNMENT 00972749; recordedBy: WW Wirth; individualCount: 1; sex: male; lifeStage: adult; **Location:** islandGroup: Hawaiian Islands; island: Oahu; country: United States; stateProvince: Hawaii; county: Honolulu; verbatimLocality: Kahuku, at light trap; **Event:** verbatimEventDate: 1.ii.1946; **Record Level:** institutionCode: USNM
- **Type status:** Other material. **Occurrence:** catalogNumber: USNMENT 00972750; recordedBy: WW Wirth; individualCount: 1; sex: male; lifeStage: adult; **Location:** islandGroup: Hawaiian Islands; island: Oahu; country: United States; stateProvince: Hawaii; county: Honolulu; verbatimLocality: Kahuku, at light trap; **Event:** verbatimEventDate: 1.ii.1946; **Record Level:** institutionCode: USNM
- **Type status:** Other material. **Occurrence:** catalogNumber: USNMENT 00972751; recordedBy: WW Wirth; individualCount: 1; sex: female; lifeStage: adult; **Location:** islandGroup: Hawaiian Islands; island: Oahu; country: United States; stateProvince: Hawaii; county: Honolulu; verbatimLocality: Kahuku, at light trap; **Event:** verbatimEventDate: 1.ii.1946; **Record Level:** institutionCode: USNM
- **Type status:** Other material. **Occurrence:** catalogNumber: USNMENT 00972752; recordedBy: WW Wirth; individualCount: 1; sex: female; lifeStage: adult; **Location:** islandGroup: Hawaiian Islands; island: Oahu; country: United States; stateProvince: Hawaii; county: Honolulu; verbatimLocality: Kahuku, at light trap; **Event:** verbatimEventDate: 1.ii.1946; **Record Level:** institutionCode: USNM
- **Type status:** Other material. **Occurrence:** catalogNumber: USNMENT 00972753; recordedBy: WW Wirth; individualCount: 1; sex: male; lifeStage: adult; **Location:** islandGroup: Hawaiian Islands; island: Oahu; country: United States; stateProvince: Hawaii; county: Honolulu; verbatimLocality: Kahuku, at light trap; **Event:** verbatimEventDate: 1.ii.1946; **Record Level:** institutionCode: USNM
- **Type status:** Other material. **Occurrence:** catalogNumber: USNMENT 00972755; recordedBy: WW Wirth; individualCount: 1; sex: male; lifeStage: adult; **Location:** islandGroup: Hawaiian Islands; island: Oahu; country: United States; stateProvince: Hawaii; county: Honolulu; verbatimLocality: Kahuku, at light trap; **Event:** verbatimEventDate: 1.ii.1946; **Record Level:** institutionCode: USNM
- **Type status:** Other material. **Occurrence:** catalogNumber: USNMENT 00972756; recordedBy: WW Wirth; individualCount: 1; sex: female; lifeStage: adult; **Location:** islandGroup: Hawaiian Islands; island: Oahu; country: United States; stateProvince: Hawaii; county: Honolulu; verbatimLocality: Kahuku, at light trap; **Event:** verbatimEventDate: 1.ii.1946; **Record Level:** institutionCode: USNM
- **Type status:** Other material. **Occurrence:** catalogNumber: USNMENT 00972755; recordedBy: CJ Davis; individualCount: 61; lifeStage: adult; **Location:** islandGroup: Hawaiian Islands; island: Oahu; country: United States; stateProvince: Hawaii; county: Hawaii; verbatimLocality: Hawaii National Park, Kilauea, at light trap; **Event:** verbatimEventDate: x.1946; **Record Level:** institutionCode: USNM
- **Type status:** Other material. **Occurrence:** recordedBy: NLH Krauss; individualCount: 1; lifeStage: adult; **Location:** islandGroup: Hawaiian Islands; island: Lanai; country: United States; stateProvince: Hawaii; county: Maui; verbatimLocality: Maunlei Gulch; **Event:** verbatimEventDate: 28.x.1947; **Record Level:** institutionCode: BPBM
- **Type status:** Other material. **Occurrence:** recordedBy: EH Bryan Jr.; individualCount: 1; lifeStage: adult; **Location:** islandGroup: Hawaiian Islands; island: Hawaii; country: United States; stateProvince: Hawaii; county: Hawaii; verbatimLocality: Kilauea; **Identification:** identifiedBy: JA Tenorio; **Event:** verbatimEventDate: 22.v.1952; **Record Level:** institutionCode: BPBM
- **Type status:** Other material. **Occurrence:** recordedBy: CP Hoyt; individualCount: 1; lifeStage: adult; **Location:** islandGroup: Hawaiian Islands; island: Hawaii; country: United States; stateProvince: Hawaii; county: Hawaii; verbatimLocality: Kaluakauka, Keanakolu Trail; verbatimElevation: 5000 ft.; **Event:** verbatimEventDate: 30.x.1952; **Record Level:** institutionCode: BPBM
- **Type status:** Other material. **Occurrence:** recordedBy: CP Hoyt; individualCount: 6; lifeStage: adult; **Location:** islandGroup: Hawaiian Islands; island: Oahu; country: United States; stateProvince: Hawaii; county: Honolulu; verbatimLocality: Mokuleia, Kukuiala Valley; **Identification:** identifiedBy: JA Tenorio; **Event:** verbatimEventDate: 13.xii.1952; **Record Level:** institutionCode: BPBM
- **Type status:** Other material. **Occurrence:** recordedBy: CP Hoyt; individualCount: 8; lifeStage: adult; **Location:** islandGroup: Hawaiian Islands; island: Oahu; country: United States; stateProvince: Hawaii; county: Honolulu; verbatimLocality: Kuliouou; verbatimElevation: 1500; **Identification:** identifiedBy: JA Tenorio; **Event:** verbatimEventDate: 7.ii.1953; **Record Level:** institutionCode: BPBM
- **Type status:** Other material. **Occurrence:** recordedBy: CP Hoyt; individualCount: 1; lifeStage: adult; **Location:** islandGroup: Hawaiian Islands; island: Oahu; country: United States; stateProvince: Hawaii; county: Honolulu; verbatimLocality: Kuliouou, B. P. Bishop Museum; verbatimElevation: 1500; **Identification:** identifiedBy: JA Tenorio; **Event:** verbatimEventDate: 7.ii.1953; **Record Level:** institutionCode: BPBM
- **Type status:** Other material. **Occurrence:** recordedBy: CP Hoyt; individualCount: 2; lifeStage: adult; **Location:** islandGroup: Hawaiian Islands; island: Oahu; country: United States; stateProvince: Hawaii; county: Honolulu; verbatimLocality: Kuliouou; verbatimElevation: 1500 ft.; **Identification:** identifiedBy: JA Tenorio; **Event:** verbatimEventDate: 7.ii.1953; **Record Level:** institutionCode: BPBM
- **Type status:** Other material. **Occurrence:** recordedBy: CP Hoyt; individualCount: 1; lifeStage: adult; **Location:** islandGroup: Hawaiian Islands; island: Oahu; country: United States; stateProvince: Hawaii; county: Honolulu; verbatimLocality: Kuliouou; verbatimElevation: 1500; **Identification:** identifiedBy: JA Tenorio; **Event:** verbatimEventDate: 7.ii.1953; **Record Level:** institutionCode: BPBM
- **Type status:** Other material. **Occurrence:** recordedBy: JL Gressit; individualCount: 3; lifeStage: adult; **Location:** islandGroup: Hawaiian Islands; island: Molokai; country: United States; stateProvince: Hawaii; county: Maui; verbatimLocality: above Waikolu Valley; verbatimElevation: 4593; **Identification:** identifiedBy: JA Tenorio; **Event:** verbatimEventDate: 28.iv.1955; **Record Level:** institutionCode: BPBM
- **Type status:** Other material. **Occurrence:** recordedBy: A Suehiro; individualCount: 1; lifeStage: adult; **Location:** islandGroup: Hawaiian Islands; island: Oahu; country: United States; stateProvince: Hawaii; county: Honolulu; verbatimLocality: Kalihi, B. P. Bishop Museum; **Identification:** identifiedBy: JA Tenorio; **Event:** verbatimEventDate: 19.iv.1958; **Record Level:** institutionCode: BPBM
- **Type status:** Other material. **Occurrence:** recordedBy: LW Quate; individualCount: 1; lifeStage: adult; **Location:** islandGroup: Hawaiian Islands; island: Hawaii; country: United States; stateProvince: Hawaii; county: Hawaii; verbatimLocality: Kohala Mts.; verbatimElevation: 1900; **Identification:** identifiedBy: JA Tenorio; **Event:** verbatimEventDate: 31.vii.1958; **Record Level:** institutionCode: BPBM
- **Type status:** Other material. **Occurrence:** recordedBy: NLH Krauss; individualCount: 1; lifeStage: adult; **Location:** islandGroup: Hawaiian Islands; island: Maui; country: United States; stateProvince: Hawaii; county: Maui; verbatimLocality: Nahiku; **Identification:** identifiedBy: JA Tenorio; **Event:** verbatimEventDate: 31.vii.1958; **Record Level:** institutionCode: BPBM
- **Type status:** Other material. **Occurrence:** recordedBy: NLH Krauss; individualCount: 14; lifeStage: adult; **Location:** islandGroup: Hawaiian Islands; island: Hawaii; country: United States; stateProvince: Hawaii; county: Hawaii; verbatimLocality: Kohala Mts.; verbatimElevation: 1900; **Identification:** identifiedBy: JA Tenorio; **Event:** verbatimEventDate: 31.vii.1958; **Record Level:** institutionCode: BPBM
- **Type status:** Other material. **Occurrence:** recordedBy: LW Quate; individualCount: 9; lifeStage: adult; **Location:** islandGroup: Hawaiian Islands; island: Maui; country: United States; stateProvince: Hawaii; county: Maui; verbatimLocality: Waikamoi; verbatimElevation: 3936; **Identification:** identifiedBy: JA Tenorio; **Event:** verbatimEventDate: 14.iii.1961; **Record Level:** institutionCode: BPBM
- **Type status:** Other material. **Occurrence:** recordedBy: NLH Krauss; individualCount: 1; lifeStage: adult; **Location:** islandGroup: Hawaiian Islands; island: Lanai; country: United States; stateProvince: Hawaii; county: Maui; verbatimLocality: Maunalei; **Identification:** identifiedBy: JA Tenorio; **Event:** verbatimEventDate: 18.ii.1965; **Record Level:** institutionCode: BPBM
- **Type status:** Other material. **Occurrence:** recordedBy: K Arakaki; individualCount: 11; lifeStage: adult; **Location:** islandGroup: Hawaiian Islands; island: Oahu; country: United States; stateProvince: Hawaii; county: Honolulu; verbatimLocality: Waiau Hawaiian Electric Power Plant, near dam at lower end of pond; verbatimElevation: 0; **Identification:** identifiedBy: K Arakaki; **Event:** verbatimEventDate: 30.vi.1998; **Record Level:** institutionCode: BPBM
- **Type status:** Other material. **Occurrence:** recordedBy: K Arakaki; individualCount: 2; lifeStage: adult; **Location:** islandGroup: Hawaiian Islands; island: Oahu; country: United States; stateProvince: Hawaii; county: Honolulu; verbatimLocality: E'oCanal; **Identification:** identifiedBy: K Arakaki; **Event:** verbatimEventDate: 27.vii.1998; **Record Level:** institutionCode: BPBM
- **Type status:** Other material. **Occurrence:** catalogNumber: EMEC7356076; recordedBy: PM O'Grady, RT Lapoint, GM Bennett, BS Ort, NA Pantoja, E Owen; lifeStage: adult; **Location:** islandGroup: Hawaiian Islands; island: Kauai; country: United States; stateProvince: Hawaii; county: Kauai; **Event:** verbatimEventDate: i.2010; **Record Level:** institutionCode: EMEC

##### Ecological interactions

###### Native status

Endemic.

##### Distribution

Hawaiian Islands: Kauai, Oahu, Molokai, Lanai, Maui, Hawaii.

#### Scatella cilipes

(Wirth, 1948)

##### Materials

- **Type status:** Holotype. **Occurrence:** catalogNumber: 1658; recordedBy: WW Wirth; individualCount: 1; sex: male; lifeStage: adult; **Location:** islandGroup: Hawaiian Islands; island: Oahu; country: United States; stateProvince: Hawaii; county: Honolulu; verbatimLocality: Mt. Kaala, bog at summit; verbatimElevation: 4000 ft.; **Event:** verbatimEventDate: 25.vii.1946; **Record Level:** institutionCode: BPBM
- **Type status:** Paratype. **Occurrence:** catalogNumber: 1658a; recordedBy: FX Williams; individualCount: 1; sex: female; lifeStage: adult; **Location:** islandGroup: Hawaiian Islands; island: Oahu; country: United States; stateProvince: Hawaii; county: Honolulu; verbatimLocality: Waianae, edge of swift water; **Event:** verbatimEventDate: 19.iv.1936; **Record Level:** institutionCode: BPBM
- **Type status:** Paratype. **Occurrence:** recordedBy: FX Williams; individualCount: 2; lifeStage: adult; **Location:** islandGroup: Hawaiian Islands; island: Oahu; country: United States; stateProvince: Hawaii; county: Honolulu; verbatimLocality: Waihi-iki, Manoa Valley, wet rocks; **Event:** verbatimEventDate: 28.ii.1937; **Record Level:** institutionCode: BPBM
- **Type status:** Paratype. **Occurrence:** recordedBy: WW Wirth; individualCount: 1; sex: male; lifeStage: adult; **Location:** islandGroup: Hawaiian Islands; island: Oahu; country: United States; stateProvince: Hawaii; county: Honolulu; verbatimLocality: Manoa Valley; **Event:** verbatimEventDate: 6.v.1945; **Record Level:** institutionCode: UHM
- **Type status:** Paratype. **Occurrence:** recordedBy: WW Wirth; individualCount: 2; sex: male; lifeStage: adult; **Location:** islandGroup: Hawaiian Islands; island: Oahu; country: United States; stateProvince: Hawaii; county: Honolulu; verbatimLocality: Manoa Valley; **Event:** verbatimEventDate: 10.iv.1946; **Record Level:** institutionCode: UHM
- **Type status:** Paratype. **Occurrence:** recordedBy: WW Wirth; individualCount: 4; lifeStage: adult; **Location:** islandGroup: Hawaiian Islands; island: Kauai; country: United States; stateProvince: Hawaii; county: Kauai; verbatimLocality: Wailua Falls, wet rocks at stream margin; **Event:** verbatimEventDate: 4.ix. 1946; **Record Level:** institutionCode: UHM
- **Type status:** Paratype. **Occurrence:** recordedBy: WW Wirth; individualCount: 1; sex: female; lifeStage: adult; **Location:** islandGroup: Hawaiian Islands; island: Kauai; country: United States; stateProvince: Hawaii; county: Kauai; verbatimLocality: Kokee; verbatimElevation: 4000 ft.; **Event:** verbatimEventDate: 7.ix.1946; **Record Level:** institutionCode: UHM
- **Type status:** Other material. **Occurrence:** recordedBy: NLH Krauss; individualCount: 3; lifeStage: adult; **Location:** islandGroup: Hawaiian Islands; island: Maui; country: United States; stateProvince: Hawaii; county: Maui; verbatimLocality: Nahiku; **Event:** verbatimEventDate: date unclear; **Record Level:** institutionCode: BPBM
- **Type status:** Other material. **Occurrence:** recordedBy: EH Bryan Jr.; individualCount: 1; lifeStage: adult; **Location:** islandGroup: Hawaiian Islands; island: Kauai; country: United States; stateProvince: Hawaii; county: Kauai; verbatimLocality: Honopu; **Identification:** identifiedBy: K Arakaki; **Event:** verbatimEventDate: 17.vi.1922; **Record Level:** institutionCode: BPBM
- **Type status:** Other material. **Occurrence:** recordedBy: EH Bryan Jr.; individualCount: 1; lifeStage: adult; **Location:** islandGroup: Hawaiian Islands; island: Oahu; country: United States; stateProvince: Hawaii; county: Honolulu; verbatimLocality: Kamokuiki Valley; **Event:** verbatimEventDate: 13.iv.1933; **Record Level:** institutionCode: BPBM
- **Type status:** Other material. **Occurrence:** recordedBy: FX Williams; individualCount: 1; lifeStage: adult; **Location:** islandGroup: Hawaiian Islands; island: Hawaii; country: United States; stateProvince: Hawaii; county: Hawaii; verbatimLocality: Akaka Falls; **Identification:** identifiedBy: JA Tenorio; **Event:** verbatimEventDate: 24.x.1933; **Record Level:** institutionCode: BPBM
- **Type status:** Other material. **Occurrence:** recordedBy: EC Zimmerman; individualCount: 1; lifeStage: adult; **Location:** islandGroup: Hawaiian Islands; island: Oahu; country: United States; stateProvince: Hawaii; county: Honolulu; verbatimLocality: Kalihi Valley; **Event:** verbatimEventDate: 13.iii.1937; **Record Level:** institutionCode: BPBM
- **Type status:** Other material. **Occurrence:** recordedBy: EC Zimmerman; individualCount: 1; lifeStage: adult; **Location:** islandGroup: Hawaiian Islands; island: Oahu; country: United States; stateProvince: Hawaii; county: Honolulu; verbatimLocality: Kalihi Valley; **Event:** verbatimEventDate: 13.iii.1937; **Record Level:** institutionCode: BPBM
- **Type status:** Other material. **Occurrence:** recordedBy: EC Zimmerman; individualCount: 1; lifeStage: adult; **Location:** islandGroup: Hawaiian Islands; island: Kauai; country: United States; stateProvince: Hawaii; county: Kauai; verbatimLocality: near Halemanu; **Event:** verbatimEventDate: 10.vii.1937; **Record Level:** institutionCode: BPBM
- **Type status:** Other material. **Occurrence:** recordedBy: NLH Krauss; individualCount: 17; lifeStage: adult; **Location:** islandGroup: Hawaiian Islands; island: Molokai; country: United States; stateProvince: Hawaii; county: Maui; verbatimLocality: Mapalehu; **Identification:** identifiedBy: K Arakaki; **Event:** verbatimEventDate: 5.xi.1947; **Record Level:** institutionCode: BPBM
- **Type status:** Other material. **Occurrence:** recordedBy: CP Hoyt; individualCount: 1; lifeStage: adult; **Location:** islandGroup: Hawaiian Islands; island: Oahu; country: United States; stateProvince: Hawaii; county: Honolulu; verbatimLocality: Lulumahu Valley; **Event:** verbatimEventDate: 20.ii.1953; **Record Level:** institutionCode: BPBM
- **Type status:** Other material. **Occurrence:** recordedBy: NLH Krauss; individualCount: 1; lifeStage: adult; **Location:** islandGroup: Hawaiian Islands; island: Maui; country: United States; stateProvince: Hawaii; county: Maui; verbatimLocality: Nahiku; **Identification:** identifiedBy: JA Tenorio; **Event:** verbatimEventDate: ii.1958; **Record Level:** institutionCode: BPBM
- **Type status:** Other material. **Occurrence:** recordedBy: LW Quate; individualCount: 2; lifeStage: adult; **Location:** islandGroup: Hawaiian Islands; island: Hawaii; country: United States; stateProvince: Hawaii; county: Hawaii; verbatimLocality: Kohala Mountains; verbatimElevation: 1900 ft.; **Event:** verbatimEventDate: 3.vii.1958; **Record Level:** institutionCode: BPBM
- **Type status:** Other material. **Occurrence:** recordedBy: LW Quate; individualCount: 2; lifeStage: adult; **Location:** islandGroup: Hawaiian Islands; island: Oahu; country: United States; stateProvince: Hawaii; county: Honolulu; verbatimLocality: Kalihi Valley; verbatimElevation: 1000 ft.; **Event:** verbatimEventDate: 10.xi.1960; **Record Level:** institutionCode: BPBM
- **Type status:** Other material. **Occurrence:** recordedBy: LW Quate; individualCount: 12; lifeStage: adult; **Location:** islandGroup: Hawaiian Islands; island: Maui; country: United States; stateProvince: Hawaii; county: Maui; verbatimLocality: Waikamoi; verbatimElevation: 3937 ft.; **Event:** verbatimEventDate: 14.iii.1961; **Record Level:** institutionCode: BPBM
- **Type status:** Other material. **Occurrence:** recordedBy: LW Quate; individualCount: 7; lifeStage: adult; **Location:** islandGroup: Hawaiian Islands; island: Hawaii; country: United States; stateProvince: Hawaii; county: Hawaii; verbatimLocality: Head of Waipio Valley; verbatimElevation: 3937 ft.; **Event:** verbatimEventDate: 23.iii.1961; **Record Level:** institutionCode: BPBM
- **Type status:** Other material. **Occurrence:** recordedBy: RCA Rice; individualCount: 3; lifeStage: adult; **Location:** islandGroup: Hawaiian Islands; island: Maui; country: United States; stateProvince: Hawaii; county: Maui; verbatimLocality: Haleakala National Park, Kaupo Gap; verbatimElevation: 5400 ft.; **Event:** verbatimEventDate: 4.vi.1976; **Record Level:** institutionCode: BPBM
- **Type status:** Other material. **Occurrence:** recordedBy: NL Evenhuis; individualCount: 1; lifeStage: adult; **Location:** islandGroup: Hawaiian Islands; island: Maui; country: United States; stateProvince: Hawaii; county: Maui; verbatimLocality: Kipahulu Valley, Palikea Stream, gauging station; verbatimElevation: 1640 ft.; **Identification:** identifiedBy: K Arakaki; **Event:** verbatimEventDate: 4.vii.1980; **Record Level:** institutionCode: BPBM
- **Type status:** Other material. **Occurrence:** recordedBy: WC Gagne; individualCount: 1; lifeStage: adult; **Location:** islandGroup: Hawaiian Islands; island: Maui; country: United States; stateProvince: Hawaii; county: Maui; verbatimLocality: Kipahulu Valley, Palikea Stream, gauging station; verbatimElevation: 492 ft.; **Identification:** identifiedBy: K Arakaki; **Event:** verbatimEventDate: 4.vii.1980; **Record Level:** institutionCode: BPBM
- **Type status:** Other material. **Occurrence:** recordedBy: WC Gagne; individualCount: 19; lifeStage: adult; **Location:** islandGroup: Hawaiian Islands; island: Maui; country: United States; stateProvince: Hawaii; county: Maui; verbatimLocality: Haleakala National Park, Kipahulu Valley, ridge E of Greensword Bog; verbatimElevation: 5947 ft.; **Identification:** identifiedBy: K Arakaki; **Event:** verbatimEventDate: 25.vi.1981; **Record Level:** institutionCode: BPBM
- **Type status:** Other material. **Occurrence:** catalogNumber: EMEC7356027; recordedBy: PM O'Grady, CD Specht, S Hotchkiss, G Schuurman, M Gianullo; individualCount: 39; lifeStage: adult; **Location:** islandGroup: Hawaiian Islands; island: Hawaii; country: United States; stateProvince: Hawaii; county: Hawaii; verbatimLocality: Kohala Mountains, Top of Waipio Falls, trail and stream; verbatimLatitude: 20°4'14.57"N; verbatimLongitude: 155°40'10.70"W; geodeticDatum: WGS84; **Identification:** identifiedBy: PM O'Grady; **Event:** verbatimEventDate: 16.vii.2004; **Record Level:** institutionCode: EMEC; collectionCode: 270.C
- **Type status:** Other material. **Occurrence:** catalogNumber: EMEC7356026; recordedBy: PM O'Grady, GM Bennett, JE Gatesy, C Hayashi; individualCount: 6; lifeStage: adult; **Location:** islandGroup: Hawaiian Islands; island: Maui; country: United States; stateProvince: Hawaii; county: Maui; verbatimLocality: Waikamoi Forest Preserve, Stream from Flume; verbatimLatitude: 20°48'45.72"N; verbatimLongitude: 156°14'17.94"W; geodeticDatum: WGS84; **Identification:** identifiedBy: PM O'Grady; **Event:** verbatimEventDate: 4.viii.2005; **Record Level:** institutionCode: EMEC; collectionCode: 300.4
- **Type status:** Other material. **Occurrence:** catalogNumber: EMEC7356019; recordedBy: PM O'Grady, RT Lapoint, GM Bennett, BS Ort, NA Pantoja; individualCount: 4; lifeStage: adult; **Location:** islandGroup: Hawaiian Islands; island: Kauai; country: United States; stateProvince: Hawaii; county: Kauai; verbatimLocality: Upper Kawaikoi Stream @ Alakai Swamp Trail Crossing; verbatimLatitude: 22°8'48.06"N; verbatimLongitude: 159°36'49.38"W; geodeticDatum: WGS84; **Identification:** identifiedBy: PM O'Grady; **Event:** verbatimEventDate: 8.i.2010; **Record Level:** institutionCode: EMEC; collectionCode: 581.5
- **Type status:** Other material. **Occurrence:** catalogNumber: EMEC7356020; recordedBy: PM O'Grady, RT Lapoint, GM Bennett, BS Ort, NA Pantoja; lifeStage: adult; **Location:** islandGroup: Hawaiian Islands; island: Kauai; country: United States; stateProvince: Hawaii; county: Kauai; verbatimLocality: Nualolo Creek, sweeping vegetation in dry creek; verbatimLatitude: 22°8'34.75"N; verbatimLongitude: 159°41'17.74"W; geodeticDatum: WGS84; **Identification:** identifiedBy: PM O'Grady; **Event:** verbatimEventDate: 9.i.2010; **Record Level:** institutionCode: EMEC; collectionCode: 584.A
- **Type status:** Other material. **Occurrence:** catalogNumber: EMEC7356021; recordedBy: PM O'Grady, RT Lapoint, GM Bennett, BS Ort; lifeStage: adult; **Location:** islandGroup: Hawaiian Islands; island: Kauai; country: United States; stateProvince: Hawaii; county: Kauai; verbatimLocality: Kokee Stream near Canyon Trail; verbatimLatitude: 22°6'18.56"N; verbatimLongitude: 159°39'41.12"W; geodeticDatum: WGS84; **Identification:** identifiedBy: PM O'Grady; **Event:** verbatimEventDate: 10.i.2010; **Record Level:** institutionCode: EMEC; collectionCode: 588.E
- **Type status:** Other material. **Occurrence:** catalogNumber: EMEC7356022; recordedBy: PM O'Grady, RT Lapoint, GM Bennett, BS Ort; individualCount: 2; lifeStage: adult; **Location:** islandGroup: Hawaiian Islands; island: Kauai; country: United States; stateProvince: Hawaii; county: Kauai; verbatimLocality: Stream #1, Trail along North Coast; verbatimLatitude: 22°12'40.15"N; verbatimLongitude: 159°35'40.29"W; geodeticDatum: WGS84; **Identification:** identifiedBy: PM O'Grady; **Event:** verbatimEventDate: 11.i.2010; **Record Level:** institutionCode: EMEC; collectionCode: 589.5
- **Type status:** Other material. **Occurrence:** catalogNumber: EMEC7356023; recordedBy: PM O'Grady, RT Lapoint, GM Bennett, BS Ort; individualCount: 1; lifeStage: adult; **Location:** islandGroup: Hawaiian Islands; island: Kauai; country: United States; stateProvince: Hawaii; county: Kauai; verbatimLocality: Hanapakai Stream, sweeping beach and stream; verbatimLatitude: 22°12'31.40"N; verbatimLongitude: 159°35'51.88"W; geodeticDatum: WGS84; **Identification:** identifiedBy: PM O'Grady; **Event:** verbatimEventDate: 11.i.2010; **Record Level:** institutionCode: EMEC; collectionCode: 590.4
- **Type status:** Other material. **Occurrence:** catalogNumber: EMEC7356024; recordedBy: PM O'Grady, RT Lapoint, GM Bennett, BS Ort; lifeStage: adult; **Location:** islandGroup: Hawaiian Islands; island: Kauai; country: United States; stateProvince: Hawaii; county: Kauai; verbatimLocality: Honokoa Trail, seep #2; verbatimLatitude: 22°12'22.91"N; verbatimLongitude: 159°36'3.96"W; geodeticDatum: WGS84; **Identification:** identifiedBy: PM O'Grady; **Event:** verbatimEventDate: 11.i.2010; **Record Level:** institutionCode: EMEC; collectionCode: 592.4
- **Type status:** Other material. **Occurrence:** catalogNumber: EMEC7356025; recordedBy: PM O'Grady, RT Lapoint, GM Bennett, BS Ort; lifeStage: adult; **Location:** islandGroup: Hawaiian Islands; island: Kauai; country: United States; stateProvince: Hawaii; county: Kauai; verbatimLocality: Honokoa Trail, seep #4; verbatimLatitude: 22°12'16.43"N; verbatimLongitude: 159°36'12.34"W; geodeticDatum: WGS84; **Identification:** identifiedBy: PM O'Grady; **Event:** verbatimEventDate: 11.i.2010; **Record Level:** institutionCode: EMEC; collectionCode: 594.7
- **Type status:** Other material. **Occurrence:** catalogNumber: EMEC7356028; recordedBy: PM O'Grady, RT Lapoint, GM Bennett, BS Ort, NA Pantoja; lifeStage: adult; **Location:** islandGroup: Hawaiian Islands; island: Hawaii; country: United States; stateProvince: Hawaii; county: Hawaii; verbatimLocality: 0.5 miles from start of scenic drive to Onomea Bay, first major bridge, sweeping along stream; verbatimLatitude: 19°47'42.20"N; verbatimLongitude: 155°5'38.58"W; geodeticDatum: WGS84; **Identification:** identifiedBy: PM O'Grady; **Event:** verbatimEventDate: 2.viii.2010; **Record Level:** institutionCode: EMEC; collectionCode: 613.3
- **Type status:** Other material. **Occurrence:** catalogNumber: EMEC7356029; recordedBy: PM O'Grady, RT Lapoint, GM Bennett, BS Ort, NA Pantoja; lifeStage: adult; **Location:** islandGroup: Hawaiian Islands; island: Hawaii; country: United States; stateProvince: Hawaii; county: Hawaii; verbatimLocality: Kohala State Forest, sweeping ditch and top of Waimanu Stream; verbatimLatitude: 20°4'14.57"N; verbatimLongitude: 155°40'10.70"W; geodeticDatum: WGS84; **Identification:** identifiedBy: PM O'Grady; **Event:** verbatimEventDate: 4.viii.2010; **Record Level:** institutionCode: EMEC; collectionCode: 621.5
- **Type status:** Other material. **Occurrence:** catalogNumber: EMEC7356030; recordedBy: PM O'Grady, RT Lapoint, GM Bennett, BS Ort, NA Pantoja; lifeStage: adult; **Location:** islandGroup: Hawaiian Islands; island: Hawaii; country: United States; stateProvince: Hawaii; county: Hawaii; verbatimLocality: Kohala Mountains, sweeping in stream where bridge crosses road, 0.5 miles before end; verbatimLatitude: 20°3'23.70"N; verbatimLongitude: 155°40'6.10"W; geodeticDatum: WGS84; **Identification:** identifiedBy: PM O'Grady; **Event:** verbatimEventDate: 4.viii.2010; **Record Level:** institutionCode: EMEC; collectionCode: 623.5
- **Type status:** Other material. **Occurrence:** catalogNumber: EMEC7356031; recordedBy: PM O'Grady, RT Lapoint, D Foote; lifeStage: adult; **Location:** islandGroup: Hawaiian Islands; island: Hawaii; country: United States; stateProvince: Hawaii; county: Hawaii; verbatimLocality: Kau Forest Reserve, Hionamoa Stream; verbatimLatitude: 19°11'49.11"N; verbatimLongitude: 155°34'42.38"W; geodeticDatum: WGS84; **Identification:** identifiedBy: PM O'Grady; **Event:** verbatimEventDate: 5.viii.2010; **Record Level:** institutionCode: EMEC; collectionCode: 628.7

##### Ecological interactions

###### Native status

Endemic.

##### Distribution

Hawaiian Islands: Kauai, Oahu, Molokai, Maui, Hawaii.

#### Scatella cinereifacies

(Tenorio, 1980)

##### Materials

- **Type status:** Holotype. **Occurrence:** recordedBy: MD Delfinado, L. Teramoto, L. Uyenishi; individualCount: 1; lifeStage: adult; **Location:** islandGroup: Hawaiian Islands; island: Kauai; country: United States; stateProvince: Hawaii; county: Kauai; verbatimLocality: Opaekaa Stream, collected on wet rocks; **Identification:** identifiedBy: JA Tenorio; **Event:** verbatimEventDate: 4.iv.1970; **Record Level:** institutionCode: BPBM
- **Type status:** Paratype. **Occurrence:** recordedBy: MD Delfinado, L. Teramoto, L. Uyenishi; individualCount: 1; lifeStage: adult; **Location:** islandGroup: Hawaiian Islands; island: Kauai; country: United States; stateProvince: Hawaii; county: Kauai; verbatimLocality: Opaekaa Stream, collected on wet rocks; **Identification:** identifiedBy: JA Tenorio; **Event:** verbatimEventDate: 4.iv.1970; **Record Level:** institutionCode: BPBM
- **Type status:** Paratype. **Occurrence:** recordedBy: EH Bryan Jr.; individualCount: 3; lifeStage: adult; **Location:** islandGroup: Hawaiian Islands; island: Kauai; country: United States; stateProvince: Hawaii; county: Kauai; verbatimLocality: Honopu; **Identification:** identifiedBy: JA Tenorio; **Event:** verbatimEventDate: 17.vi.1922; **Record Level:** institutionCode: BPBM
- **Type status:** Paratype. **Occurrence:** recordedBy: EH Bryan Jr.; individualCount: 1; lifeStage: adult; **Location:** islandGroup: Hawaiian Islands; island: Kauai; country: United States; stateProvince: Hawaii; county: Kauai; verbatimLocality: Nualolo; **Identification:** identifiedBy: JA Tenorio; **Event:** verbatimEventDate: 18.vi.1922; **Record Level:** institutionCode: BPBM
- **Type status:** Paratype. **Occurrence:** recordedBy: DE Hardy; lifeStage: adult; **Location:** islandGroup: Hawaiian Islands; island: Kauai; country: United States; stateProvince: Hawaii; county: Kauai; verbatimLocality: Kalalau Valley Trail; **Identification:** identifiedBy: JA Tenorio; **Event:** verbatimEventDate: 14.xii.1963; **Record Level:** institutionCode: UHM
- **Type status:** Paratype. **Occurrence:** recordedBy: MD Delfinado, L. Teramoto, L. Uyenishi; individualCount: 3; lifeStage: adult; **Location:** islandGroup: Hawaiian Islands; island: Kauai; country: United States; stateProvince: Hawaii; county: Kauai; verbatimLocality: Opaekaa Stream, collected on wet rocks; **Identification:** identifiedBy: JA Tenorio; **Event:** verbatimEventDate: 4.iv.1970; **Record Level:** institutionCode: BPBM
- **Type status:** Paratype. **Occurrence:** recordedBy: MD Delfinado, L. Teramoto, L. Uyenishi; individualCount: 8; lifeStage: adult; **Location:** islandGroup: Hawaiian Islands; island: Kauai; country: United States; stateProvince: Hawaii; county: Kauai; verbatimLocality: Opaekaa Stream, collected on wet rocks; **Identification:** identifiedBy: JA Tenorio; **Event:** verbatimEventDate: 4.iv.1970; **Record Level:** institutionCode: UHM
- **Type status:** Paratype. **Occurrence:** recordedBy: MD Delfinado, L. Teramoto, L. Uyenishi; lifeStage: adult; **Location:** islandGroup: Hawaiian Islands; island: Kauai; country: United States; stateProvince: Hawaii; county: Kauai; verbatimLocality: Waipoo Falls, on wet rocks; **Identification:** identifiedBy: JA Tenorio; **Event:** verbatimEventDate: 4.iv.1970; **Record Level:** institutionCode: UHM
- **Type status:** Other material. **Occurrence:** catalogNumber: EMEC7356032; recordedBy: PM O'Grady, RT Lapoint, GM Bennett, BS Ort, NA Pantoja; individualCount: 1; lifeStage: adult; **Location:** islandGroup: Hawaiian Islands; island: Kauai; country: United States; stateProvince: Hawaii; county: Kauai; verbatimLocality: Nualolo Creek, sweeping vegetation in dry creek; verbatimLatitude: 22°8'34.75"N; verbatimLongitude: 159°41'17.74"W; geodeticDatum: WGS84; **Identification:** identifiedBy: PM O'Grady; **Event:** verbatimEventDate: 9.i.2010; **Record Level:** institutionCode: EMEC; collectionCode: 584.B
- **Type status:** Other material. **Occurrence:** catalogNumber: EMEC7356033; recordedBy: PM O'Grady, RT Lapoint, GM Bennett, BS Ort, NA Pantoja; lifeStage: adult; **Location:** islandGroup: Hawaiian Islands; island: Kauai; country: United States; stateProvince: Hawaii; county: Kauai; verbatimLocality: Kokee Stream near Canyon Trail; verbatimLatitude: 22°6'18.56"N; verbatimLongitude: 159°39'41.12"W; geodeticDatum: WGS84; **Identification:** identifiedBy: PM O'Grady; **Event:** verbatimEventDate: 10.i.2010; **Record Level:** institutionCode: EMEC; collectionCode: 588.A
- **Type status:** Other material. **Occurrence:** catalogNumber: EMEC7356034; recordedBy: PM O'Grady, RT Lapoint, GM Bennett, BS Ort; individualCount: 2; lifeStage: adult; **Location:** islandGroup: Hawaiian Islands; island: Kauai; country: United States; stateProvince: Hawaii; county: Kauai; verbatimLocality: Honokoa Trail, seep #2; verbatimLatitude: 22°12'22.91"N; verbatimLongitude: 159°36'3.96"W; geodeticDatum: WGS84; **Identification:** identifiedBy: PM O'Grady; **Event:** verbatimEventDate: 11.i.2010; **Record Level:** institutionCode: EMEC; collectionCode: 592.5
- **Type status:** Other material. **Occurrence:** catalogNumber: EMEC7356016; recordedBy: PM O'Grady, KN Magnacca, RT Lapoint, GM Bennett; lifeStage: adult; **Location:** islandGroup: Hawaiian Islands; island: Molokai; country: United States; stateProvince: Hawaii; county: Maui; verbatimLocality: Kamakou Forest Reserve, sweeping in stream before tunnel; verbatimLatitude: 21°6'42.78"N; verbatimLongitude: 156°54'25.20"W; geodeticDatum: WGS84; **Identification:** identifiedBy: PM O'Grady; **Event:** verbatimEventDate: 19.ii.2007; **Record Level:** institutionCode: EMEC; collectionCode: 379.A

##### Ecological interactions

###### Native status

Endemic.

##### Distribution

Hawaiian Islands: Kauai.

#### Scatella clavipes

(Wirth, 1948)

##### Materials

- **Type status:** Holotype. **Occurrence:** catalogNumber: 1659; recordedBy: WW Wirth; individualCount: 1; sex: male; lifeStage: adult; **Location:** islandGroup: Hawaiian Islands; island: Hawaii; country: United States; stateProvince: Hawaii; county: Hawaii; verbatimLocality: Akaka Falls; **Event:** verbatimEventDate: 5.iii.1946; **Record Level:** institutionCode: BPBM
- **Type status:** Paratype. **Occurrence:** catalogNumber: 1659a; recordedBy: WW Wirth; individualCount: 1; sex: female; lifeStage: adult; **Location:** islandGroup: Hawaiian Islands; island: Hawaii; country: United States; stateProvince: Hawaii; county: Hawaii; verbatimLocality: Akaka Falls; **Event:** verbatimEventDate: 5.iii.1946; **Record Level:** institutionCode: BPBM
- **Type status:** Paratype. **Occurrence:** recordedBy: WW Wirth; individualCount: 7; lifeStage: adult; **Location:** islandGroup: Hawaiian Islands; island: Hawaii; country: United States; stateProvince: Hawaii; county: Hawaii; verbatimLocality: Akaka Falls; **Event:** verbatimEventDate: 5.iii.1946; **Record Level:** institutionCode: BPBM
- **Type status:** Paratype. **Occurrence:** recordedBy: WW Wirth; individualCount: 64; lifeStage: adult; **Location:** islandGroup: Hawaiian Islands; island: Hawaii; country: United States; stateProvince: Hawaii; county: Hawaii; verbatimLocality: Akaka Falls; **Event:** verbatimEventDate: 5.iii.1946; **Record Level:** institutionCode: UHM
- **Type status:** Paratype. **Occurrence:** recordedBy: WW Wirth; individualCount: 22; lifeStage: adult; **Location:** islandGroup: Hawaiian Islands; island: Hawaii; country: United States; stateProvince: Hawaii; county: Hawaii; verbatimLocality: Rainbow Falls, Wailuku River; **Event:** verbatimEventDate: 2.iii.1946; **Record Level:** institutionCode: UHM
- **Type status:** Other material. **Occurrence:** recordedBy: EH Bryan Jr.; individualCount: 1; lifeStage: adult; **Location:** islandGroup: Hawaiian Islands; island: Kauai; country: United States; stateProvince: Hawaii; county: Kauai; verbatimLocality: Awaawapuhi; **Identification:** identifiedBy: JA Tenorio; **Event:** verbatimEventDate: 16.vi.1922; **Record Level:** institutionCode: BPBM
- **Type status:** Other material. **Occurrence:** recordedBy: O Bryant; individualCount: 1; lifeStage: adult; **Location:** islandGroup: Hawaiian Islands; island: Maui; country: United States; stateProvince: Hawaii; county: Maui; verbatimLocality: Olinda; verbatimElevation: 4500 ft.; **Identification:** identifiedBy: WW Wirth; **Event:** verbatimEventDate: 15.iii.1932; **Record Level:** institutionCode: BPBM
- **Type status:** Other material. **Occurrence:** recordedBy: NLH Krauss; individualCount: 6; lifeStage: adult; **Location:** islandGroup: Hawaiian Islands; island: Maui; country: United States; stateProvince: Hawaii; county: Maui; verbatimLocality: Olinda; **Event:** verbatimEventDate: 1.viii.1932; **Record Level:** institutionCode: BPBM
- **Type status:** Other material. **Occurrence:** recordedBy: WW Wirth; individualCount: 1; sex: male; lifeStage: adult; previousIdentifications: holotype of N. clavipes tenda; **Location:** islandGroup: Hawaiian Islands; island: Oahu; country: United States; stateProvince: Hawaii; county: Honolulu; verbatimLocality: Kaluanui Valley; verbatimElevation: 2000 ft.; **Event:** verbatimEventDate: 14.iii.1946; **Record Level:** institutionCode: BPBM
- **Type status:** Other material. **Occurrence:** recordedBy: WW Wirth; individualCount: 1; sex: female; lifeStage: adult; previousIdentifications: allotype of N. clavipes tenda; **Location:** islandGroup: Hawaiian Islands; island: Oahu; country: United States; stateProvince: Hawaii; county: Honolulu; verbatimLocality: Kaluanui Valley; verbatimElevation: 2000 ft.; **Event:** verbatimEventDate: 14.iii.1946; **Record Level:** institutionCode: BPBM
- **Type status:** Other material. **Occurrence:** catalogNumber: USNMENT 00972732; recordedBy: WW Wirth; individualCount: 1; sex: male; lifeStage: adult; previousIdentifications: paratype of N. clavipes tenda; **Location:** islandGroup: Hawaiian Islands; island: Oahu; country: United States; stateProvince: Hawaii; county: Honolulu; verbatimLocality: Kaluanui Valley; verbatimElevation: 2000 ft.; **Event:** verbatimEventDate: 14.iii.1946; **Record Level:** institutionCode: USNM
- **Type status:** Other material. **Occurrence:** recordedBy: WW Wirth; individualCount: 1; sex: male; lifeStage: adult; previousIdentifications: paratype of N. clavipes tenda; **Location:** islandGroup: Hawaiian Islands; island: Oahu; country: United States; stateProvince: Hawaii; county: Honolulu; verbatimLocality: Kaluanui Valley; verbatimElevation: 2000 ft.; **Event:** verbatimEventDate: 14.iii.1946; **Record Level:** institutionCode: BPBM
- **Type status:** Other material. **Occurrence:** recordedBy: NLH Krauss; individualCount: 5; lifeStage: adult; **Location:** islandGroup: Hawaiian Islands; island: Maui; country: United States; stateProvince: Hawaii; county: Maui; verbatimLocality: Nahiku; **Event:** verbatimEventDate: ii.1958; **Record Level:** institutionCode: BPBM
- **Type status:** Other material. **Occurrence:** recordedBy: LW Quate; individualCount: 2; lifeStage: adult; **Location:** islandGroup: Hawaiian Islands; island: Hawaii; country: United States; stateProvince: Hawaii; county: Hawaii; verbatimLocality: Kohala Mountains; verbatimElevation: 1900 ft.; **Event:** verbatimEventDate: 31.vii.1958; **Record Level:** institutionCode: BPBM
- **Type status:** Other material. **Occurrence:** recordedBy: LW Quate; individualCount: 3; lifeStage: adult; **Location:** islandGroup: Hawaiian Islands; island: Maui; country: United States; stateProvince: Hawaii; county: Maui; verbatimLocality: Waikamoi; verbatimElevation: 3936 ft.; **Identification:** identifiedBy: WW Wirth; **Event:** verbatimEventDate: 14.iii.1961; **Record Level:** institutionCode: BPBM
- **Type status:** Other material. **Occurrence:** recordedBy: JL Gressitt; individualCount: 1; lifeStage: adult; **Location:** islandGroup: Hawaiian Islands; island: Molokai; country: United States; stateProvince: Hawaii; county: Maui; verbatimLocality: West Halawa Valley; verbatimElevation: 164 ft.; **Event:** verbatimEventDate: 23.iii.1970; **Record Level:** institutionCode: BPBM
- **Type status:** Other material. **Occurrence:** recordedBy: WA Steffan; individualCount: 1; lifeStage: adult; **Location:** islandGroup: Hawaiian Islands; island: Maui; country: United States; stateProvince: Hawaii; county: Maui; verbatimLocality: Kipahulu Valley, Pualuu Stream; verbatimElevation: 984 ft.; **Event:** verbatimEventDate: 21.vii.1980; **Record Level:** institutionCode: BPBM
- **Type status:** Other material. **Occurrence:** catalogNumber: EMEC7356041; recordedBy: PM O'Grady, RT Lapoint, GM Bennett, BS Ort, NA Pantoja, E Owen; lifeStage: adult; **Location:** islandGroup: Hawaiian Islands; island: Kauai; country: United States; stateProvince: Hawaii; county: Kauai; verbatimLocality: Kawaikoi Stream at Sugi Grove; verbatimLatitude: 22°7'53.12"N; verbatimLongitude: 159°37'18.12"W; geodeticDatum: WGS84; **Identification:** identifiedBy: PM O'Grady; **Event:** verbatimEventDate: 4.i.2010; **Record Level:** institutionCode: EMEC; collectionCode: 567.6
- **Type status:** Other material. **Occurrence:** catalogNumber: EMEC7356035; recordedBy: PM O'Grady, RT Lapoint, GM Bennett, BS Ort; individualCount: 7; lifeStage: adult; **Location:** islandGroup: Hawaiian Islands; island: Kauai; country: United States; stateProvince: Hawaii; county: Kauai; verbatimLocality: Hanapakai Stream, sweeping beach and stream; verbatimLatitude: 22°12'31.40"N; verbatimLongitude: 159°35'51.88"W; geodeticDatum: WGS84; **Identification:** identifiedBy: PM O'Grady; **Event:** verbatimEventDate: 11.i.2010; **Record Level:** institutionCode: EMEC; collectionCode: 590.3
- **Type status:** Other material. **Occurrence:** catalogNumber: EMEC7356042; recordedBy: PM O'Grady, RT Lapoint, GM Bennett, BS Ort; lifeStage: adult; **Location:** islandGroup: Hawaiian Islands; island: Kauai; country: United States; stateProvince: Hawaii; county: Kauai; verbatimLocality: Honokoa Trail, seep #2; verbatimLatitude: 22°12'22.91"N; verbatimLongitude: 159°36'3.96"W; geodeticDatum: WGS84; **Identification:** identifiedBy: PM O'Grady; **Event:** verbatimEventDate: 11.i.2010; **Record Level:** institutionCode: EMEC; collectionCode: 592.2
- **Type status:** Other material. **Occurrence:** catalogNumber: EMEC7356036; recordedBy: PM O'Grady, RT Lapoint, GM Bennett, BS Ort, NA Pantoja; lifeStage: adult; **Location:** islandGroup: Hawaiian Islands; island: Hawaii; country: United States; stateProvince: Hawaii; county: Hawaii; verbatimLocality: 0.5 miles from start of scenic drive to Onomea Bay, first major bridge, sweeping along stream; verbatimLatitude: 19°47'42.20"N; verbatimLongitude: 155°5'38.58"W; geodeticDatum: WGS84; **Identification:** identifiedBy: PM O'Grady; **Event:** verbatimEventDate: 2.viii.2010; **Record Level:** institutionCode: EMEC; collectionCode: 613.2
- **Type status:** Other material. **Occurrence:** catalogNumber: EMEC7356038; recordedBy: PM O'Grady, RT Lapoint, GM Bennett, BS Ort, NA Pantoja; lifeStage: adult; **Location:** islandGroup: Hawaiian Islands; island: Hawaii; country: United States; stateProvince: Hawaii; county: Hawaii; verbatimLocality: Alakahi Trail to Onomea Bay, adjacent to Hawaiia Botanical Garden, sweeping rocks in surf; verbatimLatitude: 19°48'32.54"N; verbatimLongitude: 155°5'34.98"W; geodeticDatum: WGS84; **Identification:** identifiedBy: PM O'Grady; **Event:** verbatimEventDate: 2.viii.2010; **Record Level:** institutionCode: EMEC; collectionCode: 614.2
- **Type status:** Other material. **Occurrence:** catalogNumber: EMEC7356039; recordedBy: PM O'Grady, RT Lapoint, GM Bennett, BS Ort, NA Pantoja; lifeStage: adult; **Location:** islandGroup: Hawaiian Islands; island: Hawaii; country: United States; stateProvince: Hawaii; county: Hawaii; verbatimLocality: Kawainui Stream, 6 ton bridge on Scenic Loop; verbatimLatitude: 19°48'42.10"N; verbatimLongitude: 155°5'37.88"W; geodeticDatum: WGS84; **Identification:** identifiedBy: PM O'Grady; **Event:** verbatimEventDate: 2.viii.2010; **Record Level:** institutionCode: EMEC; collectionCode: 615.2
- **Type status:** Other material. **Occurrence:** catalogNumber: EMEC7356037; recordedBy: PM O'Grady, RT Lapoint, GM Bennett, BS Ort, NA Pantoja; lifeStage: adult; **Location:** islandGroup: Hawaiian Islands; island: Hawaii; country: United States; stateProvince: Hawaii; county: Hawaii; verbatimLocality: Kolekole Beach Park, sweeping along ocean coast; verbatimLatitude: 19°52'57.93"N; verbatimLongitude: 155°7'9.22"W; geodeticDatum: WGS84; **Identification:** identifiedBy: PM O'Grady; **Event:** verbatimEventDate: 2.viii.2010; **Record Level:** institutionCode: EMEC; collectionCode: 616.2
- **Type status:** Other material. **Occurrence:** catalogNumber: EMEC7356040; recordedBy: PM O'Grady, RT Lapoint, GM Bennett, BS Ort, NA Pantoja; lifeStage: adult; **Location:** islandGroup: Hawaiian Islands; island: Hawaii; country: United States; stateProvince: Hawaii; county: Hawaii; verbatimLocality: Waipio Stream, near the base of He'elawae Falls; verbatimLatitude: 20°4'36.46"N; verbatimLongitude: 155°37'8.72"W; geodeticDatum: WGS84; **Identification:** identifiedBy: PM O'Grady; **Event:** verbatimEventDate: 2.viii.2010; **Record Level:** institutionCode: EMEC; collectionCode: 617.1
- **Type status:** Other material. **Occurrence:** catalogNumber: EMEC7356043; recordedBy: BS Ort; individualCount: 1; sex: male; lifeStage: adult; **Location:** islandGroup: Hawaiian Islands; island: Hawaii; country: United States; stateProvince: Hawaii; county: Hawaii; verbatimLocality: Ohanakupa Rd, 1st bridge at south end of road; verbatimLatitude: 19°47'42.20"N; verbatimLongitude: 155°5'38.58"W; geodeticDatum: WGS84; **Identification:** identifiedBy: PM O'Grady; **Event:** verbatimEventDate: 2.viii.2010; **Record Level:** institutionCode: EMEC; collectionCode: BO7.1
- **Type status:** Other material. **Occurrence:** catalogNumber: EMEC7356044; recordedBy: BS Ort; individualCount: 1; sex: male; lifeStage: adult; **Location:** islandGroup: Hawaiian Islands; island: Hawaii; country: United States; stateProvince: Hawaii; county: Hawaii; verbatimLocality: Waipio Valley, near base of He'eilawe Falls at dammed reservoir; verbatimLatitude: 20°4'36.46"N; verbatimLongitude: 155°37'8.72"W; geodeticDatum: WGS84; **Identification:** identifiedBy: PM O'Grady; **Event:** verbatimEventDate: 2.viii.2010; **Record Level:** institutionCode: EMEC; collectionCode: BO10.2
- **Type status:** Other material. **Occurrence:** catalogNumber: EMEC7356045; recordedBy: BS Ort; individualCount: 3; sex: females; lifeStage: adult; **Location:** islandGroup: Hawaiian Islands; island: Hawaii; country: United States; stateProvince: Hawaii; county: Hawaii; verbatimLocality: Waipio Valley, near base of He'eilawe Falls at dammed reservoir; verbatimLatitude: 20°4'36.46"N; verbatimLongitude: 155°37'8.72"W; geodeticDatum: WGS84; **Identification:** identifiedBy: PM O'Grady; **Event:** verbatimEventDate: 2.viii.2010; **Record Level:** institutionCode: EMEC; collectionCode: BO10.2

##### Ecological interactions

###### Native status

Endemic.

##### Distribution

Hawaiian Islands: Kauai, Oahu, Molokai, Maui, Hawaii.

##### Notes

52 specimens from USNM not examined.

#### Scatella femoralis

(Tenorio, 1980)

##### Materials

- **Type status:** Holotype. **Occurrence:** recordedBy: M Tamashiro; individualCount: 1; sex: male; lifeStage: adult; **Location:** islandGroup: Hawaiian Islands; island: Maui; country: United States; stateProvince: Hawaii; county: Maui; verbatimLocality: Puu Nianiau; **Event:** verbatimEventDate: iv.1954; **Record Level:** institutionCode: UHM

##### Ecological interactions

###### Native status

Endemic.

##### Distribution

Hawaiian Islands: Maui.

#### Scatella fluvialis

(Tenorio, 1980)

##### Materials

- **Type status:** Holotype. **Occurrence:** catalogNumber: 9477; recordedBy: JA Tenorio; individualCount: 1; sex: male; lifeStage: adult; **Location:** islandGroup: Hawaiian Islands; island: Hawaii; country: United States; stateProvince: Hawaii; county: Hawaii; verbatimLocality: Wailuku River; verbatimElevation: 2170 ft.; **Identification:** identifiedBy: JA Tenorio; **Event:** verbatimEventDate: 14.viii.1970; **Record Level:** institutionCode: BPBM
- **Type status:** Paratype. **Occurrence:** catalogNumber: 9477a; recordedBy: JA Tenorio; individualCount: 1; sex: female; lifeStage: adult; **Location:** islandGroup: Hawaiian Islands; island: Hawaii; country: United States; stateProvince: Hawaii; county: Hawaii; verbatimLocality: Wailuku River; verbatimElevation: 2170 ft.; **Identification:** identifiedBy: JA Tenorio; **Event:** verbatimEventDate: 14.viii.1970; **Record Level:** institutionCode: BPBM
- **Type status:** Paratype. **Occurrence:** recordedBy: JA Tenorio; individualCount: 5; lifeStage: adult; **Location:** islandGroup: Hawaiian Islands; island: Hawaii; country: United States; stateProvince: Hawaii; county: Hawaii; verbatimLocality: Wailuku River; verbatimElevation: 2170 ft.; **Identification:** identifiedBy: JA Tenorio; **Event:** verbatimEventDate: 14.viii.1970; **Record Level:** institutionCode: BPBM
- **Type status:** Paratype. **Occurrence:** recordedBy: DE Hardy; lifeStage: adult; **Location:** islandGroup: Hawaiian Islands; island: Hawaii; country: United States; stateProvince: Hawaii; county: Hawaii; verbatimLocality: Wailuku River; verbatimElevation: 2170 ft.; **Identification:** identifiedBy: JA Tenorio; **Event:** verbatimEventDate: 14.viii.1970; **Record Level:** institutionCode: UHM
- **Type status:** Paratype. **Occurrence:** recordedBy: JA Tenorio; individualCount: 3; lifeStage: adult; **Location:** islandGroup: Hawaiian Islands; island: Hawaii; country: United States; stateProvince: Hawaii; county: Hawaii; verbatimLocality: Wailuku River; verbatimElevation: 2000 ft.; **Identification:** identifiedBy: JA Tenorio; **Event:** verbatimEventDate: 14.viii.1970; **Record Level:** institutionCode: BPBM
- **Type status:** Paratype. **Occurrence:** recordedBy: JA Tenorio; lifeStage: adult; **Location:** islandGroup: Hawaiian Islands; island: Oahu; country: United States; stateProvince: Hawaii; county: Honolulu; verbatimLocality: Kahana Stream; verbatimElevation: 180 ft.; **Identification:** identifiedBy: JA Tenorio; **Event:** verbatimEventDate: 27.viii.1970; **Record Level:** institutionCode: UHM

##### Ecological interactions

###### Native status

Endemic.

##### Distribution

Hawaiian Islands: Oahu, Hawaii.

#### Scatella hawaiiensis

Grimshaw, 1901

##### Materials

- **Type status:** Syntype. **Occurrence:** catalogNumber: 15628; recordedBy: no collector given; individualCount: 8; lifeStage: adult; **Location:** islandGroup: Hawaiian Islands; island: Oahu; country: United States; stateProvince: Hawaii; county: Honolulu; verbatimLocality: Mt. Kaala; verbatimElevation: 2000 ft.; **Event:** verbatimEventDate: iii.1893; **Record Level:** institutionCode: BPBM
- **Type status:** Syntype. **Occurrence:** recordedBy: no collector given; individualCount: 2; lifeStage: adult; **Location:** islandGroup: Hawaiian Islands; island: Oahu; country: United States; stateProvince: Hawaii; county: Honolulu; verbatimLocality: Mt. Kaala; verbatimElevation: 2000 ft.; **Event:** verbatimEventDate: iii.1893; **Record Level:** institutionCode: BMNH
- **Type status:** Other material. **Occurrence:** recordedBy: JF Illingworth; individualCount: 2; lifeStage: adult; **Location:** islandGroup: Hawaiian Islands; island: Oahu; country: United States; stateProvince: Hawaii; county: Honolulu; verbatimLocality: Waianae Mts.; **Identification:** identifiedBy: ET Cresson; **Event:** verbatimEventDate: no date given; **Record Level:** institutionCode: BPBM
- **Type status:** Other material. **Occurrence:** recordedBy: JF Illingworth; individualCount: 5; sex: female; lifeStage: adult; **Location:** islandGroup: Hawaiian Islands; island: Oahu; country: United States; stateProvince: Hawaii; county: Honolulu; verbatimLocality: Waianae Mts.; **Identification:** identifiedBy: ET Cresson; **Event:** verbatimEventDate: no date given; **Record Level:** institutionCode: UHM
- **Type status:** Other material. **Occurrence:** recordedBy: O Bryant; individualCount: 1; sex: female; lifeStage: adult; **Location:** islandGroup: Hawaiian Islands; island: Maui; country: United States; stateProvince: Hawaii; county: Maui; verbatimLocality: Kula Pipe Line; verbatimElevation: 5000 ft.; **Event:** verbatimEventDate: no date given; **Record Level:** institutionCode: BPBM
- **Type status:** Other material. **Occurrence:** recordedBy: FW Terry; individualCount: 1; lifeStage: adult; **Location:** islandGroup: Hawaiian Islands; island: Hawaii; country: United States; stateProvince: Hawaii; county: Hawaii; verbatimLocality: Hutchinson Plant.; **Event:** verbatimEventDate: 1903; **Record Level:** institutionCode: BPBM
- **Type status:** Other material. **Occurrence:** recordedBy: WM Giffard; individualCount: 1; lifeStage: adult; **Location:** islandGroup: Hawaiian Islands; island: Oahu; country: United States; stateProvince: Hawaii; county: Honolulu; verbatimLocality: Mt. Tantalus; verbatimElevation: 1300; **Event:** verbatimEventDate: 3.iii.1907; **Record Level:** institutionCode: BPBM
- **Type status:** Other material. **Occurrence:** recordedBy: no collector given; individualCount: 1; lifeStage: adult; **Location:** islandGroup: Hawaiian Islands; island: Oahu; country: United States; stateProvince: Hawaii; county: Honolulu; verbatimLocality: Waimanalo; **Event:** verbatimEventDate: 7.x.1907; **Record Level:** institutionCode: BPBM
- **Type status:** Other material. **Occurrence:** recordedBy: no collector given; individualCount: 1; lifeStage: adult; **Location:** islandGroup: Hawaiian Islands; island: Oahu; country: United States; stateProvince: Hawaii; county: Honolulu; verbatimLocality: Waimanalo; **Event:** verbatimEventDate: 7.xi.1907; **Record Level:** institutionCode: BPBM
- **Type status:** Other material. **Occurrence:** recordedBy: JF Illingworth; individualCount: 1; lifeStage: adult; **Location:** islandGroup: Hawaiian Islands; island: Oahu; country: United States; stateProvince: Hawaii; county: Honolulu; verbatimLocality: Palolo Falls; **Event:** verbatimEventDate: vii.1916; **Record Level:** institutionCode: UHM
- **Type status:** Other material. **Occurrence:** recordedBy: JF Illingworth; individualCount: 3; lifeStage: adult; **Location:** islandGroup: Hawaiian Islands; island: Oahu; country: United States; stateProvince: Hawaii; county: Honolulu; verbatimLocality: Palolo Falls; **Identification:** identifiedBy: ET Cresson; **Event:** verbatimEventDate: vii.1916; **Record Level:** institutionCode: BPBM
- **Type status:** Other material. **Occurrence:** recordedBy: EH Bryan Jr.; individualCount: 1; lifeStage: adult; **Location:** islandGroup: Hawaiian Islands; island: Oahu; country: United States; stateProvince: Hawaii; county: Honolulu; verbatimLocality: Waianae; **Event:** verbatimEventDate: 25.ii.1920; **Record Level:** institutionCode: BPBM
- **Type status:** Other material. **Occurrence:** recordedBy: OH Swezey; individualCount: 2; lifeStage: adult; **Location:** islandGroup: Hawaiian Islands; island: Oahu; country: United States; stateProvince: Hawaii; county: Honolulu; verbatimLocality: Mt. Kaala, Makaleha; **Identification:** identifiedBy: ET Cresson; **Event:** verbatimEventDate: 8.i.1922; **Record Level:** institutionCode: BPBM
- **Type status:** Other material. **Occurrence:** recordedBy: OH Swezey; individualCount: 1; lifeStage: adult; **Location:** islandGroup: Hawaiian Islands; island: Oahu; country: United States; stateProvince: Hawaii; county: Honolulu; verbatimLocality: Mt. Tantalus; **Identification:** identifiedBy: ET Cresson; **Event:** verbatimEventDate: 2.ii.1922; **Record Level:** institutionCode: BPBM
- **Type status:** Other material. **Occurrence:** recordedBy: OH Swezey; individualCount: 2; lifeStage: adult; **Location:** islandGroup: Hawaiian Islands; island: Oahu; country: United States; stateProvince: Hawaii; county: Honolulu; verbatimLocality: Palolo; **Event:** verbatimEventDate: 26.ii.1922; **Record Level:** institutionCode: BPBM
- **Type status:** Other material. **Occurrence:** recordedBy: OH Swezey; individualCount: 2; lifeStage: adult; **Location:** islandGroup: Hawaiian Islands; island: Oahu; country: United States; stateProvince: Hawaii; county: Honolulu; verbatimLocality: Moanalua; **Identification:** identifiedBy: WW Wirth; **Event:** verbatimEventDate: 9.iv.1922; **Record Level:** institutionCode: BPBM
- **Type status:** Other material. **Occurrence:** recordedBy: OH Swezey; individualCount: 1; sex: male; lifeStage: adult; **Location:** islandGroup: Hawaiian Islands; island: Oahu; country: United States; stateProvince: Hawaii; county: Honolulu; verbatimLocality: Moanalua; **Event:** verbatimEventDate: 9.iv.1922; **Record Level:** institutionCode: UHM
- **Type status:** Other material. **Occurrence:** recordedBy: OH Swezey; individualCount: 5; lifeStage: adult; **Location:** islandGroup: Hawaiian Islands; island: Oahu; country: United States; stateProvince: Hawaii; county: Honolulu; verbatimLocality: Moanalua; **Identification:** identifiedBy: WW Wirth; **Event:** verbatimEventDate: 9.iv.1922; **Record Level:** institutionCode: BPBM
- **Type status:** Other material. **Occurrence:** recordedBy: EH Bryan Jr.; individualCount: 3; sex: female; lifeStage: adult; **Location:** islandGroup: Hawaiian Islands; island: Kauai; country: United States; stateProvince: Hawaii; county: Kauai; verbatimLocality: Awaawapuhi; **Event:** verbatimEventDate: 16.vi.1922; **Record Level:** institutionCode: BPBM
- **Type status:** Other material. **Occurrence:** recordedBy: EH Bryan Jr.; individualCount: 1; lifeStage: adult; **Location:** islandGroup: Hawaiian Islands; island: Kauai; country: United States; stateProvince: Hawaii; county: Kauai; verbatimLocality: Honopu; **Identification:** identifiedBy: ET Cresson; **Event:** verbatimEventDate: 17.vi.1922; **Record Level:** institutionCode: BPBM
- **Type status:** Other material. **Occurrence:** recordedBy: EH Bryan Jr.; individualCount: 1; lifeStage: adult; **Location:** islandGroup: Hawaiian Islands; island: Oahu; country: United States; stateProvince: Hawaii; county: Honolulu; verbatimLocality: Kalihi, Kamehameha; **Event:** verbatimEventDate: 12.ii.1923; **Record Level:** institutionCode: BPBM
- **Type status:** Other material. **Occurrence:** recordedBy: EH Bryan Jr.; individualCount: 1; lifeStage: adult; **Location:** islandGroup: Hawaiian Islands; island: Oahu; country: United States; stateProvince: Hawaii; county: Honolulu; verbatimLocality: Honolulu; **Event:** verbatimEventDate: 2.iv.1923; **Record Level:** institutionCode: BPBM
- **Type status:** Other material. **Occurrence:** recordedBy: OH Swezey; individualCount: 3; lifeStage: adult; **Location:** islandGroup: Hawaiian Islands; island: Oahu; country: United States; stateProvince: Hawaii; county: Honolulu; verbatimLocality: Niu, waterfall; **Identification:** identifiedBy: ET Cresson; **Event:** verbatimEventDate: 13.i.1924; **Record Level:** institutionCode: BPBM
- **Type status:** Other material. **Occurrence:** recordedBy: OH Swezey; individualCount: 4; lifeStage: adult; **Location:** islandGroup: Hawaiian Islands; island: Oahu; country: United States; stateProvince: Hawaii; county: Honolulu; verbatimLocality: Niu, waterfall; **Event:** verbatimEventDate: 13.i.1924; **Record Level:** institutionCode: UHM
- **Type status:** Other material. **Occurrence:** recordedBy: WM Giffard; individualCount: 2; lifeStage: adult; **Location:** islandGroup: Hawaiian Islands; island: Hawaii; country: United States; stateProvince: Hawaii; county: Hawaii; verbatimLocality: Olaa, 29 miles; **Event:** verbatimEventDate: viii.1925; **Record Level:** institutionCode: BPBM
- **Type status:** Other material. **Occurrence:** recordedBy: EH Bryan Jr.; individualCount: 9; lifeStage: adult; **Location:** islandGroup: Hawaiian Islands; island: Oahu; country: United States; stateProvince: Hawaii; county: Honolulu; verbatimLocality: Makaleha; **Identification:** identifiedBy: WW Wirth; **Event:** verbatimEventDate: 13.i.1929; **Record Level:** institutionCode: BPBM
- **Type status:** Other material. **Occurrence:** recordedBy: EH Bryan Jr.; individualCount: 1; lifeStage: adult; **Location:** islandGroup: Hawaiian Islands; island: Oahu; country: United States; stateProvince: Hawaii; county: Honolulu; verbatimLocality: Makaleha; **Event:** verbatimEventDate: 13.i.1929; **Record Level:** institutionCode: UHM
- **Type status:** Other material. **Occurrence:** recordedBy: O Bryant; individualCount: 1; lifeStage: adult; **Location:** islandGroup: Hawaiian Islands; island: Oahu; country: United States; stateProvince: Hawaii; county: Honolulu; verbatimLocality: Honolulu; **Event:** verbatimEventDate: 8.ii.1932; **Record Level:** institutionCode: BPBM
- **Type status:** Other material. **Occurrence:** recordedBy: O Bryant; individualCount: 1; sex: female; lifeStage: adult; **Location:** islandGroup: Hawaiian Islands; island: Maui; country: United States; stateProvince: Hawaii; county: Maui; verbatimLocality: Olinda; verbatimElevation: 4500; **Identification:** identifiedBy: WW Wirth; **Event:** verbatimEventDate: 15.iii.1932; **Record Level:** institutionCode: BPBM
- **Type status:** Other material. **Occurrence:** recordedBy: O Bryant; individualCount: 5; lifeStage: adult; **Location:** islandGroup: Hawaiian Islands; island: Maui; country: United States; stateProvince: Hawaii; county: Maui; verbatimLocality: Olinda; verbatimElevation: 5000-6000; **Identification:** identifiedBy: JA Tenorio; **Event:** verbatimEventDate: 19.iii.1932; **Record Level:** institutionCode: BPBM
- **Type status:** Other material. **Occurrence:** recordedBy: O Bryant; individualCount: 2; lifeStage: adult; **Location:** islandGroup: Hawaiian Islands; island: Maui; country: United States; stateProvince: Hawaii; county: Maui; verbatimLocality: Kula Pipeline; verbatimElevation: 4500; **Identification:** identifiedBy: JA Tenorio; **Event:** verbatimEventDate: 19.iii.1932; **Record Level:** institutionCode: BPBM
- **Type status:** Other material. **Occurrence:** recordedBy: O Bryant; individualCount: 1; lifeStage: adult; **Location:** islandGroup: Hawaiian Islands; island: Maui; country: United States; stateProvince: Hawaii; county: Maui; verbatimLocality: Kula Pipeline; verbatimElevation: 4500; **Identification:** identifiedBy: WW Wirth; **Event:** verbatimEventDate: 19.iii.1932; **Record Level:** institutionCode: BPBM
- **Type status:** Other material. **Occurrence:** recordedBy: O Bryant; individualCount: 3; lifeStage: adult; **Location:** islandGroup: Hawaiian Islands; island: Maui; country: United States; stateProvince: Hawaii; county: Maui; verbatimLocality: Haleakala Crater; verbatimElevation: 7000; **Identification:** identifiedBy: WW Wirth; **Event:** verbatimEventDate: 22.iii.1932; **Record Level:** institutionCode: BPBM
- **Type status:** Other material. **Occurrence:** recordedBy: O Bryant; individualCount: 1; lifeStage: adult; **Location:** islandGroup: Hawaiian Islands; island: Maui; country: United States; stateProvince: Hawaii; county: Maui; verbatimLocality: Kula Pipeline; verbatimElevation: 4500; **Identification:** identifiedBy: JA Tenorio; **Event:** verbatimEventDate: 3.iv.1932; **Record Level:** institutionCode: BPBM
- **Type status:** Other material. **Occurrence:** recordedBy: O Bryant; individualCount: 6; lifeStage: adult; **Location:** islandGroup: Hawaiian Islands; island: Maui; country: United States; stateProvince: Hawaii; county: Maui; verbatimLocality: Kula Pipeline; verbatimElevation: 4500; **Identification:** identifiedBy: JA Tenorio; **Event:** verbatimEventDate: 8.iv.1932; **Record Level:** institutionCode: BPBM
- **Type status:** Other material. **Occurrence:** recordedBy: O Bryant; individualCount: 1; lifeStage: adult; **Location:** islandGroup: Hawaiian Islands; island: Maui; country: United States; stateProvince: Hawaii; county: Maui; verbatimLocality: Olinda; verbatimElevation: 4500; **Identification:** identifiedBy: ET Cresson; **Event:** verbatimEventDate: 8.iv.1932; **Record Level:** institutionCode: BPBM
- **Type status:** Other material. **Occurrence:** recordedBy: EH Bryan Jr.; individualCount: 3; lifeStage: adult; **Location:** islandGroup: Hawaiian Islands; island: Kauai; country: United States; stateProvince: Hawaii; county: Kauai; verbatimLocality: Awaawapuhi; **Identification:** identifiedBy: WW Wirth; **Event:** verbatimEventDate: 16.vi.1932; **Record Level:** institutionCode: BPBM
- **Type status:** Other material. **Occurrence:** recordedBy: EH Bryan Jr.; individualCount: 11; lifeStage: adult; **Location:** islandGroup: Hawaiian Islands; island: Oahu; country: United States; stateProvince: Hawaii; county: Honolulu; verbatimLocality: Kamokuiki Valley, about water pool; **Event:** verbatimEventDate: 13.iv.1933; **Record Level:** institutionCode: BPBM
- **Type status:** Other material. **Occurrence:** recordedBy: EH Bryan Jr.; individualCount: 5; lifeStage: adult; **Location:** islandGroup: Hawaiian Islands; island: Oahu; country: United States; stateProvince: Hawaii; county: Honolulu; verbatimLocality: Castle Trail; **Event:** verbatimEventDate: 14.x.1934; **Record Level:** institutionCode: BPBM
- **Type status:** Other material. **Occurrence:** recordedBy: WM Giffard; individualCount: 1; lifeStage: adult; **Location:** islandGroup: Hawaiian Islands; island: Oahu; country: United States; stateProvince: Hawaii; county: Honolulu; verbatimLocality: Maunawili; verbatimElevation: 800; **Event:** verbatimEventDate: 25.ii.1935; **Record Level:** institutionCode: BPBM
- **Type status:** Other material. **Occurrence:** recordedBy: RL Usinger; individualCount: 1; lifeStage: adult; **Location:** islandGroup: Hawaiian Islands; island: Kauai; country: United States; stateProvince: Hawaii; county: Kauai; verbatimLocality: Kumuwela; **Event:** verbatimEventDate: 30.xii.1935; **Record Level:** institutionCode: BPBM
- **Type status:** Other material. **Occurrence:** recordedBy: EC Zimmerman; individualCount: 3; lifeStage: adult; **Location:** islandGroup: Hawaiian Islands; island: Oahu; country: United States; stateProvince: Hawaii; county: Honolulu; verbatimLocality: Waianae Mts.; **Event:** verbatimEventDate: 22.ii.1937; **Record Level:** institutionCode: BPBM
- **Type status:** Other material. **Occurrence:** catalogNumber: USNMENT 00972734; recordedBy: WW Wirth; individualCount: 1; sex: female; lifeStage: adult; **Location:** islandGroup: Hawaiian Islands; island: Oahu; country: United States; stateProvince: Hawaii; county: Honolulu; **Event:** verbatimEventDate: 29.iv.1945; **Record Level:** institutionCode: USNM
- **Type status:** Other material. **Occurrence:** catalogNumber: USNMENT 00972735; recordedBy: WW Wirth; individualCount: 1; sex: male; lifeStage: adult; **Location:** islandGroup: Hawaiian Islands; island: Oahu; country: United States; stateProvince: Hawaii; county: Honolulu; verbatimLocality: Hering Valley; **Event:** verbatimEventDate: 29.iv.1945; **Record Level:** institutionCode: USNM
- **Type status:** Other material. **Occurrence:** catalogNumber: USNMENT 00972736; recordedBy: WW Wirth; individualCount: 1; sex: female; lifeStage: adult; **Location:** islandGroup: Hawaiian Islands; island: Oahu; country: United States; stateProvince: Hawaii; county: Honolulu; verbatimLocality: Hering Valley; **Event:** verbatimEventDate: 29.iv.1945; **Record Level:** institutionCode: USNM
- **Type status:** Other material. **Occurrence:** recordedBy: WW Wirth; individualCount: 3; lifeStage: adult; **Location:** islandGroup: Hawaiian Islands; island: Oahu; country: United States; stateProvince: Hawaii; county: Honolulu; verbatimLocality: Mt. Kaala; verbatimElevation: 2000 ft.; **Event:** verbatimEventDate: iii.1946; **Record Level:** institutionCode: BPBM
- **Type status:** Other material. **Occurrence:** catalogNumber: USNMENT 00972737; recordedBy: WW Wirth; individualCount: 1; sex: female; lifeStage: adult; **Location:** islandGroup: Hawaiian Islands; island: Oahu; country: United States; stateProvince: Hawaii; county: Honolulu; verbatimLocality: Hering Valley; verbatimElevation: 2000 ft.; **Event:** verbatimEventDate: iii.1946; **Record Level:** institutionCode: USNM
- **Type status:** Other material. **Occurrence:** catalogNumber: USNMENT 00972733; recordedBy: WW Wirth; individualCount: 1; sex: female; lifeStage: adult; **Location:** islandGroup: Hawaiian Islands; island: Hawaii; country: United States; stateProvince: Hawaii; county: Hawaii; verbatimLocality: Mt. Kaala; verbatimElevation: 4200 ft.; **Event:** verbatimEventDate: 4.iii.1946; **Record Level:** institutionCode: USNM
- **Type status:** Other material. **Occurrence:** recordedBy: WW Wirth; individualCount: 5; lifeStage: adult; **Location:** islandGroup: Hawaiian Islands; island: Oahu; country: United States; stateProvince: Hawaii; county: Honolulu; verbatimLocality: Manoa Valley; **Event:** verbatimEventDate: 10.iv.1946; **Record Level:** institutionCode: USNM
- **Type status:** Other material. **Occurrence:** catalogNumber: USNMENT 00972738; recordedBy: WW Wirth; individualCount: 1; sex: female; lifeStage: adult; **Location:** islandGroup: Hawaiian Islands; island: Oahu; country: United States; stateProvince: Hawaii; county: Honolulu; verbatimLocality: Manoa Valley; **Event:** verbatimEventDate: 17.iv.1946; **Record Level:** institutionCode: USNM
- **Type status:** Other material. **Occurrence:** recordedBy: WW Wirth; individualCount: 1; sex: female; lifeStage: adult; **Location:** islandGroup: Hawaiian Islands; island: Oahu; country: United States; stateProvince: Hawaii; county: Honolulu; verbatimLocality: Mt. Kaala; **Event:** verbatimEventDate: 17.iv.1946; **Record Level:** institutionCode: BPBM
- **Type status:** Other material. **Occurrence:** catalogNumber: USNMENt 00972739; recordedBy: WW Wirth; individualCount: 1; sex: male; lifeStage: adult; **Location:** islandGroup: Hawaiian Islands; island: Oahu; country: United States; stateProvince: Hawaii; county: Honolulu; verbatimLocality: Mt. Kaala; **Event:** verbatimEventDate: 6.v.1945; **Record Level:** institutionCode: USNM
- **Type status:** Other material. **Occurrence:** recordedBy: WW Wirth; individualCount: 1; sex: male; lifeStage: adult; **Location:** islandGroup: Hawaiian Islands; island: Oahu; country: United States; stateProvince: Hawaii; county: Honolulu; verbatimLocality: Manoa Valley; **Event:** verbatimEventDate: 6.v.1945; **Record Level:** institutionCode: BPBM
- **Type status:** Other material. **Occurrence:** recordedBy: WW Wirth; individualCount: 2; lifeStage: adult; **Location:** islandGroup: Hawaiian Islands; island: Oahu; country: United States; stateProvince: Hawaii; county: Honolulu; verbatimLocality: Kaluanui Valley, at stream; verbatimElevation: 2000 ft.; **Event:** verbatimEventDate: 14.v.1946; **Record Level:** institutionCode: BPBM
- **Type status:** Other material. **Occurrence:** catalogNumber: USNMENT 00972743; recordedBy: WW Wirth; individualCount: 1; sex: male; lifeStage: adult; **Location:** islandGroup: Hawaiian Islands; island: Oahu; country: United States; stateProvince: Hawaii; county: Honolulu; verbatimLocality: Kaluanui Valley, at stream; verbatimElevation: 2000 ft.; **Event:** verbatimEventDate: 14.v.1946; **Record Level:** institutionCode: USNM
- **Type status:** Other material. **Occurrence:** catalogNumber: USNMENT 00972740; recordedBy: WW Wirth; individualCount: 1; sex: female; lifeStage: adult; **Location:** islandGroup: Hawaiian Islands; island: Oahu; country: United States; stateProvince: Hawaii; county: Honolulu; verbatimLocality: Kaluanui Valley, at stream; verbatimElevation: 4000 ft.; **Event:** verbatimEventDate: 25.vii.1946; **Record Level:** institutionCode: USNM
- **Type status:** Other material. **Occurrence:** catalogNumber: USNMENT 00972742; recordedBy: WW Wirth; individualCount: 1; sex: female; lifeStage: adult; **Location:** islandGroup: Hawaiian Islands; island: Oahu; country: United States; stateProvince: Hawaii; county: Honolulu; verbatimLocality: Manoa Valley; verbatimElevation: 2000 ft.; **Event:** verbatimEventDate: 14.v.1946; **Record Level:** institutionCode: USNM
- **Type status:** Other material. **Occurrence:** recordedBy: WW Wirth; individualCount: 2; sex: female; lifeStage: adult; **Location:** islandGroup: Hawaiian Islands; island: Oahu; country: United States; stateProvince: Hawaii; county: Honolulu; verbatimLocality: Mt. Kaala, bog at summit; verbatimElevation: 4000 ft.; **Event:** verbatimEventDate: 25.vii.1946; **Record Level:** institutionCode: BPBM
- **Type status:** Other material. **Occurrence:** catalogNumber: USNMENT 00972741; recordedBy: WW Wirth; individualCount: 1; sex: female; lifeStage: adult; **Location:** islandGroup: Hawaiian Islands; island: Oahu; country: United States; stateProvince: Hawaii; county: Honolulu; verbatimLocality: Mt. Kaala, bog at summit; verbatimElevation: 4000 ft.; **Event:** verbatimEventDate: 25.vii.1946; **Record Level:** institutionCode: USNM
- **Type status:** Other material. **Occurrence:** catalogNumber: USNMENT 00972744; recordedBy: WW Wirth; individualCount: 1; sex: male; lifeStage: adult; **Location:** islandGroup: Hawaiian Islands; island: Kauai; country: United States; stateProvince: Hawaii; county: Kauai; verbatimLocality: Mt. Kaala, bog at summit; verbatimElevation: 4000 ft.; **Event:** verbatimEventDate: 6.ix.1946; **Record Level:** institutionCode: USNM
- **Type status:** Other material. **Occurrence:** recordedBy: Y Kondo; individualCount: 5; lifeStage: adult; **Location:** islandGroup: Hawaiian Islands; island: Oahu; country: United States; stateProvince: Hawaii; county: Honolulu; verbatimLocality: Laulaupoe Gulch; **Event:** verbatimEventDate: 9.vii.1951; **Record Level:** institutionCode: BPBM
- **Type status:** Other material. **Occurrence:** recordedBy: CP Hoyt; individualCount: 8; lifeStage: adult; **Location:** islandGroup: Hawaiian Islands; island: Oahu; country: United States; stateProvince: Hawaii; county: Honolulu; verbatimLocality: Mokuleia, Kukuiala Valley; **Identification:** identifiedBy: JA Tenorio; **Event:** verbatimEventDate: 13.xii.1952; **Record Level:** institutionCode: BPBM
- **Type status:** Other material. **Occurrence:** recordedBy: CP Hoyt; individualCount: 16; lifeStage: adult; **Location:** islandGroup: Hawaiian Islands; island: Oahu; country: United States; stateProvince: Hawaii; county: Honolulu; verbatimLocality: Maunawili; verbatimElevation: 1200; **Identification:** identifiedBy: JA Tenorio; **Event:** verbatimEventDate: 31.i.1953; **Record Level:** institutionCode: BPBM
- **Type status:** Other material. **Occurrence:** recordedBy: CP Hoyt; individualCount: 52; lifeStage: adult; **Location:** islandGroup: Hawaiian Islands; island: Oahu; country: United States; stateProvince: Hawaii; county: Honolulu; verbatimLocality: Maunawili; verbatimElevation: 1200; **Identification:** identifiedBy: JA Tenorio; **Event:** verbatimEventDate: 31.i.1953; **Record Level:** institutionCode: BPBM
- **Type status:** Other material. **Occurrence:** recordedBy: CP Hoyt; individualCount: 1; lifeStage: adult; **Location:** islandGroup: Hawaiian Islands; island: Oahu; country: United States; stateProvince: Hawaii; county: Honolulu; verbatimLocality: Kuliouou; verbatimElevation: 1500; **Identification:** identifiedBy: JA Tenorio; **Event:** verbatimEventDate: 7.ii.1953; **Record Level:** institutionCode: BPBM
- **Type status:** Other material. **Occurrence:** recordedBy: CP Hoyt; individualCount: 10; lifeStage: adult; **Location:** islandGroup: Hawaiian Islands; island: Oahu; country: United States; stateProvince: Hawaii; county: Honolulu; verbatimLocality: Mt. Kaala; **Identification:** identifiedBy: JA Tenorio; **Event:** verbatimEventDate: 3.iv.1953; **Record Level:** institutionCode: BPBM
- **Type status:** Other material. **Occurrence:** recordedBy: JL Gressitt; individualCount: 1; lifeStage: adult; **Location:** islandGroup: Hawaiian Islands; island: Molokai; country: United States; stateProvince: Hawaii; county: Maui; verbatimLocality: Waikolu Valley; verbatimElevation: 4593; **Identification:** identifiedBy: JA Tenorio; **Event:** verbatimEventDate: 1.v.1955; **Record Level:** institutionCode: BPBM
- **Type status:** Other material. **Occurrence:** recordedBy: LW Quate; individualCount: 2; lifeStage: adult; **Location:** islandGroup: Hawaiian Islands; island: Hawaii; country: United States; stateProvince: Hawaii; county: Hawaii; verbatimLocality: Kohala Mts.; verbatimElevation: 1900; **Identification:** identifiedBy: JA Tenorio; **Event:** verbatimEventDate: 31.vii.1958; **Record Level:** institutionCode: BPBM
- **Type status:** Other material. **Occurrence:** recordedBy: LW Quate; individualCount: 3; lifeStage: adult; **Location:** islandGroup: Hawaiian Islands; island: Maui; country: United States; stateProvince: Hawaii; county: Maui; verbatimLocality: Waikamoi; verbatimElevation: 3936; **Identification:** identifiedBy: JA Tenorio; **Event:** verbatimEventDate: 14.iii.1961; **Record Level:** institutionCode: BPBM
- **Type status:** Other material. **Occurrence:** recordedBy: NLH Krauss; individualCount: 3; lifeStage: adult; **Location:** islandGroup: Hawaiian Islands; island: Kauai; country: United States; stateProvince: Hawaii; county: Kauai; verbatimLocality: Haena; **Identification:** identifiedBy: JA Tenorio; **Event:** verbatimEventDate: 10.iii.1963; **Record Level:** institutionCode: BPBM
- **Type status:** Other material. **Occurrence:** recordedBy: GA Samuelson; individualCount: 1; lifeStage: adult; **Location:** islandGroup: Hawaiian Islands; island: Oahu; country: United States; stateProvince: Hawaii; county: Honolulu; verbatimLocality: Mt. Tantalus; verbatimElevation: 984-1968; **Identification:** identifiedBy: JA Tenorio; **Event:** verbatimEventDate: 26.i.1964; **Record Level:** institutionCode: BPBM
- **Type status:** Other material. **Occurrence:** recordedBy: NLH Krauss; individualCount: 1; lifeStage: adult; **Location:** islandGroup: Hawaiian Islands; island: Lanai; country: United States; stateProvince: Hawaii; county: Maui; verbatimLocality: Lanai Mts.; **Identification:** identifiedBy: JA Tenorio; **Event:** verbatimEventDate: 17.ii.1965; **Record Level:** institutionCode: BPBM
- **Type status:** Other material. **Occurrence:** recordedBy: R. Burkhart; individualCount: 5; lifeStage: adult; **Location:** islandGroup: Hawaiian Islands; island: Maui; country: United States; stateProvince: Hawaii; county: Maui; verbatimLocality: Haleakalae National Park, Haleakala Crater, Paliku; verbatimElevation: 6400; **Identification:** identifiedBy: DE Hardy; **Event:** verbatimEventDate: 24.vi.1975; **Record Level:** institutionCode: BPBM
- **Type status:** Other material. **Occurrence:** recordedBy: R. Burkhart; individualCount: 2; lifeStage: adult; **Location:** islandGroup: Hawaiian Islands; island: Maui; country: United States; stateProvince: Hawaii; county: Maui; verbatimLocality: Haleakalae National Park, Haleakala Crater, Paliku; verbatimElevation: 6900; **Identification:** identifiedBy: DE Hardy; **Event:** verbatimEventDate: 27.vi.1975; **Record Level:** institutionCode: BPBM
- **Type status:** Other material. **Occurrence:** recordedBy: R. Burkhart; individualCount: 1; lifeStage: adult; **Location:** islandGroup: Hawaiian Islands; island: Maui; country: United States; stateProvince: Hawaii; county: Maui; verbatimLocality: Haleakalae National Park, Haleakala Crater, Paliku; verbatimElevation: 6500; **Identification:** identifiedBy: DE Hardy; **Event:** verbatimEventDate: 27.vi.1975; **Record Level:** institutionCode: BPBM
- **Type status:** Other material. **Occurrence:** recordedBy: R. Burkhart; individualCount: 1; lifeStage: adult; **Location:** islandGroup: Hawaiian Islands; island: Maui; country: United States; stateProvince: Hawaii; county: Maui; verbatimLocality: Haleakala National Park, Kaupo Gap; verbatimElevation: 6100; **Identification:** identifiedBy: DE Hardy; **Event:** verbatimEventDate: 3.vii.1975; **Record Level:** institutionCode: BPBM
- **Type status:** Other material. **Occurrence:** recordedBy: WC Gagne; individualCount: 1; lifeStage: adult; **Location:** islandGroup: Hawaiian Islands; island: Maui; country: United States; stateProvince: Hawaii; county: Maui; verbatimLocality: Haleakala National Park, Kipahulu Valley; verbatimElevation: 5002; **Identification:** identifiedBy: K Arakaki; **Event:** verbatimEventDate: 25.xi.1980; **Record Level:** institutionCode: BPBM
- **Type status:** Other material. **Occurrence:** recordedBy: GM Nishida; individualCount: 3; lifeStage: adult; **Location:** islandGroup: Hawaiian Islands; island: Oahu; country: United States; stateProvince: Hawaii; county: Honolulu; verbatimLocality: Puhawai Aqueduct, 0.25 miles E of Lulualei; verbatimElevation: 850; **Identification:** identifiedBy: K Arakaki; **Event:** verbatimEventDate: 7.iii.1996; **Record Level:** institutionCode: BPBM
- **Type status:** Other material. **Occurrence:** recordedBy: GA Samuelson; individualCount: 1; lifeStage: adult; **Location:** islandGroup: Hawaiian Islands; island: Oahu; country: United States; stateProvince: Hawaii; county: Honolulu; verbatimLocality: Lualualei Valley, Puhawai, stream gulch, 0.4 km E of aqueduct; verbatimElevation: 1050; **Identification:** identifiedBy: K Arakaki; **Event:** verbatimEventDate: 6.iii.1996; **Record Level:** institutionCode: BPBM
- **Type status:** Other material. **Occurrence:** recordedBy: GM Nishida; individualCount: 1; lifeStage: adult; **Location:** islandGroup: Hawaiian Islands; island: Oahu; country: United States; stateProvince: Hawaii; county: Honolulu; verbatimLocality: Lualualei Valley, Puhawai Waterfall; **Identification:** identifiedBy: K Arakaki; **Event:** verbatimEventDate: 23.ii.1996; **Record Level:** institutionCode: BPBM
- **Type status:** Other material. **Occurrence:** catalogNumber: EMEC7356048; recordedBy: PM O'Grady, GM Bennett, JE Gatesy, C Hayashi; individualCount: 22; lifeStage: adult; **Location:** islandGroup: Hawaiian Islands; island: Maui; country: United States; stateProvince: Hawaii; county: Maui; verbatimLocality: Manoa Valley; verbatimLatitude: 20°48'45.72"N; verbatimLongitude: 156°14'17.94"W; geodeticDatum: WGS84; **Identification:** identifiedBy: PM O'Grady; **Event:** verbatimEventDate: 4.viii.2005; **Record Level:** institutionCode: EMEC; collectionCode: 300.5
- **Type status:** Other material. **Occurrence:** catalogNumber: EMEC7356047; recordedBy: PM O'Grady, KN Magnacca, RT Lapoint, GM Bennett; individualCount: many; lifeStage: adult; **Location:** islandGroup: Hawaiian Islands; island: Maui; country: United States; stateProvince: Hawaii; county: Maui; verbatimLocality: Kaupo Gap Trail, on seep near Lobelia; verbatimLatitude: 20°42'35.88"N; verbatimLongitude: 156°8'26.82"W; geodeticDatum: WGS84; **Identification:** identifiedBy: PM O'Grady; **Event:** verbatimEventDate: 4.viii.2007; **Record Level:** institutionCode: EMEC; collectionCode: 400.1
- **Type status:** Other material. **Occurrence:** catalogNumber: EMEC7356049; recordedBy: PM O'Grady, RT Lapoint, GM Bennett, BS Ort, NA Pantoja, E Owen; lifeStage: adult; **Location:** islandGroup: Hawaiian Islands; island: Kauai; country: United States; stateProvince: Hawaii; county: Kauai; verbatimLocality: Kawaikoi Stream at Sugi Grove; verbatimLatitude: 22°7'53.12"N; verbatimLongitude: 159°37'18.12"W; geodeticDatum: WGS84; **Identification:** identifiedBy: PM O'Grady; **Event:** verbatimEventDate: 4.i.2010; **Record Level:** institutionCode: EMEC; collectionCode: 567.5
- **Type status:** Other material. **Occurrence:** catalogNumber: EMEC7356050; recordedBy: PM O'Grady, RT Lapoint, GM Bennett, BS Ort, NA Pantoja, E Owen; lifeStage: adult; **Location:** islandGroup: Hawaiian Islands; island: Kauai; country: United States; stateProvince: Hawaii; county: Kauai; verbatimLocality: Kawaikoi Stream Trail Sugi Grove to 0.5 miles; verbatimLatitude: 22°7'57.31"N; verbatimLongitude: 159°37'2.13"W; geodeticDatum: WGS84; **Identification:** identifiedBy: PM O'Grady; **Event:** verbatimEventDate: 4.i.2010; **Record Level:** institutionCode: EMEC; collectionCode: 568.6
- **Type status:** Other material. **Occurrence:** catalogNumber: EMEC7356051; recordedBy: PM O'Grady, RT Lapoint, GM Bennett, BS Ort, NA Pantoja, E Owen; individualCount: 1; lifeStage: adult; **Location:** islandGroup: Hawaiian Islands; island: Kauai; country: United States; stateProvince: Hawaii; county: Kauai; verbatimLocality: Kohua Ridge Trail, Mohihi Stream, 0-1.0 miles, sweeping stream; verbatimLatitude: 22°6'44.61"N; verbatimLongitude: 159°36'21.69"W; geodeticDatum: WGS84; **Identification:** identifiedBy: PM O'Grady; **Event:** verbatimEventDate: 6.i.2010; **Record Level:** institutionCode: EMEC; collectionCode: 574.5
- **Type status:** Other material. **Occurrence:** catalogNumber: EMEC7356046; recordedBy: PM O'Grady, RT Lapoint, GM Bennett, BS Ort, NA Pantoja, E Owen; individualCount: 4; lifeStage: adult; **Location:** islandGroup: Hawaiian Islands; island: Kauai; country: United States; stateProvince: Hawaii; county: Kauai; verbatimLocality: Upper Kawaikoi Stream @ Alakai Swamp Trail Crossing; verbatimLatitude: 22°8'48.06"N; verbatimLongitude: 159°36'49.38"W; geodeticDatum: WGS84; **Identification:** identifiedBy: PM O'Grady; **Event:** verbatimEventDate: 8.i.2010; **Record Level:** institutionCode: EMEC; collectionCode: 581.4
- **Type status:** Other material. **Occurrence:** catalogNumber: EMEC7356052; recordedBy: PM O'Grady, RT Lapoint, GM Bennett, BS Ort, NA Pantoja; lifeStage: adult; **Location:** islandGroup: Hawaiian Islands; island: Kauai; country: United States; stateProvince: Hawaii; county: Kauai; verbatimLocality: Nualolo Creek, sweeping vegetation in dry creek; verbatimLatitude: 22°8'34.75"N; verbatimLongitude: 159°41'17.74"W; geodeticDatum: WGS84; **Identification:** identifiedBy: PM O'Grady; **Event:** verbatimEventDate: 9.i.2010; **Record Level:** institutionCode: EMEC; collectionCode: 584.9
- **Type status:** Other material. **Occurrence:** catalogNumber: EMEC7356053; recordedBy: PM O'Grady, RT Lapoint, GM Bennett, BS Ort, NA Pantoja; lifeStage: adult; **Location:** islandGroup: Hawaiian Islands; island: Kauai; country: United States; stateProvince: Hawaii; county: Kauai; verbatimLocality: Kokee Stream near Canyon Trail; verbatimLatitude: 22°6'18.56"N; verbatimLongitude: 159°39'41.12"W; geodeticDatum: WGS84; **Identification:** identifiedBy: PM O'Grady; **Event:** verbatimEventDate: 10.i.2010; **Record Level:** institutionCode: EMEC; collectionCode: 588.C
- **Type status:** Other material. **Occurrence:** catalogNumber: EMEC7356054; recordedBy: PM O'Grady, RT Lapoint, GM Bennett, BS Ort; lifeStage: adult; **Location:** islandGroup: Hawaiian Islands; island: Kauai; country: United States; stateProvince: Hawaii; county: Kauai; verbatimLocality: Stream #1, Trail along North Coast; verbatimLatitude: 22°12'40.15"N; verbatimLongitude: 159°35'40.29"W; geodeticDatum: WGS84; **Identification:** identifiedBy: PM O'Grady; **Event:** verbatimEventDate: 11.i.2010; **Record Level:** institutionCode: EMEC; collectionCode: 589.4
- **Type status:** Other material. **Occurrence:** catalogNumber: EMEC7356055; recordedBy: PM O'Grady, RT Lapoint, GM Bennett, BS Ort; lifeStage: adult; **Location:** islandGroup: Hawaiian Islands; island: Kauai; country: United States; stateProvince: Hawaii; county: Kauai; verbatimLocality: Honokoa Trail, seep #2; verbatimLatitude: 22°12'22.91"N; verbatimLongitude: 159°36'3.96"W; geodeticDatum: WGS84; **Identification:** identifiedBy: PM O'Grady; **Event:** verbatimEventDate: 11.i.2010; **Record Level:** institutionCode: EMEC; collectionCode: 592.6
- **Type status:** Other material. **Occurrence:** catalogNumber: EMEC7356056; recordedBy: PM O'Grady, RT Lapoint, GM Bennett, BS Ort; individualCount: 2; lifeStage: adult; **Location:** islandGroup: Hawaiian Islands; island: Kauai; country: United States; stateProvince: Hawaii; county: Kauai; verbatimLocality: Honokoa Trail, seep #2; verbatimLatitude: 22°12'22.91"N; verbatimLongitude: 159°36'3.96"W; geodeticDatum: WGS84; **Identification:** identifiedBy: PM O'Grady; **Event:** verbatimEventDate: 11.i.2010; **Record Level:** institutionCode: EMEC; collectionCode: 592.3
- **Type status:** Other material. **Occurrence:** catalogNumber: EMEC7356057; recordedBy: PM O'Grady, RT Lapoint, GM Bennett, BS Ort; individualCount: many; lifeStage: adult; **Location:** islandGroup: Hawaiian Islands; island: Kauai; country: United States; stateProvince: Hawaii; county: Kauai; verbatimLocality: Honokoa Trail, seep #4; verbatimLatitude: 22°12'16.43"N; verbatimLongitude: 159°36'12.34"W; geodeticDatum: WGS84; **Identification:** identifiedBy: PM O'Grady; **Event:** verbatimEventDate: 11.i.2010; **Record Level:** institutionCode: EMEC; collectionCode: 594.6
- **Type status:** Other material. **Occurrence:** catalogNumber: EMEC7356077; recordedBy: KN Magnacca; lifeStage: adult; **Location:** islandGroup: Hawaiian Islands; island: Kauai; country: United States; stateProvince: Hawaii; county: Kauai; verbatimLocality: Gulch on Puu o Kila Road; geodeticDatum: WGS84; **Identification:** identifiedBy: PM O'Grady; **Event:** verbatimEventDate: 25.vii.2010; **Record Level:** institutionCode: EMEC
- **Type status:** Other material. **Occurrence:** catalogNumber: EMEC7356058; recordedBy: PM O'Grady, RT Lapoint, D Foote; lifeStage: adult; **Location:** islandGroup: Hawaiian Islands; island: Hawaii; country: United States; stateProvince: Hawaii; county: Hawaii; verbatimLocality: Kau Forest Reserve, Alahi Spring; verbatimLatitude: 19°13'40.71"N; verbatimLongitude: 155°31'6.27"W; geodeticDatum: WGS84; **Identification:** identifiedBy: PM O'Grady; **Event:** verbatimEventDate: 6.viii.2010; **Record Level:** institutionCode: EMEC; collectionCode: 631.8
- **Type status:** Other material. **Occurrence:** catalogNumber: EMEC7356059; recordedBy: PM O'Grady, RT Lapoint, GM Bennett, BS Ort, NA Pantoja; lifeStage: adult; **Location:** islandGroup: Hawaiian Islands; island: Hawaii; country: United States; stateProvince: Hawaii; county: Hawaii; verbatimLocality: Kohala State Forest, sweeping ditch and top of Waimanu Stream; verbatimLatitude: 20°4'14.57"N; verbatimLongitude: 155°40'10.70"W; geodeticDatum: WGS84; **Identification:** identifiedBy: PM O'Grady; **Event:** verbatimEventDate: 4.viii.2010; **Record Level:** institutionCode: EMEC; collectionCode: 621.4

##### Ecological interactions

###### Native status

Endemic.

##### Distribution

Hawaiian Islands: Kauai, Oahu, Molokai, Lanai, Maui, Hawaii.

##### Notes

15 specimens from USNM not examined.

#### Scatella kauaiensis

(Wirth, 1948)

##### Materials

- **Type status:** Holotype. **Occurrence:** catalogNumber: 1656; recordedBy: WW Wirth; individualCount: 1; sex: male; lifeStage: adult; **Location:** islandGroup: Hawaiian Islands; island: Kauai; country: United States; stateProvince: Hawaii; county: Kauai; verbatimLocality: Kokee, at falls near tunnel; verbatimElevation: 4000 ft.; **Event:** verbatimEventDate: 6.ix.1946; **Record Level:** institutionCode: BPBM
- **Type status:** Paratype. **Occurrence:** catalogNumber: 1656a; recordedBy: WW Wirth; individualCount: 1; sex: female; lifeStage: adult; **Location:** islandGroup: Hawaiian Islands; island: Kauai; country: United States; stateProvince: Hawaii; county: Kauai; verbatimLocality: Kokee, at falls near tunnel; verbatimElevation: 4000 ft.; **Event:** verbatimEventDate: 6.ix.1946; **Record Level:** institutionCode: BPBM
- **Type status:** Paratype. **Occurrence:** recordedBy: WW Wirth; individualCount: 45; lifeStage: adult; **Location:** islandGroup: Hawaiian Islands; island: Kauai; country: United States; stateProvince: Hawaii; county: Kauai; verbatimLocality: Kokee, at falls near tunnel; verbatimElevation: 4000 ft.; **Event:** verbatimEventDate: 6.ix.1946; **Record Level:** institutionCode: UHM
- **Type status:** Paratype. **Occurrence:** recordedBy: WW Wirth; individualCount: 3; lifeStage: adult; **Location:** islandGroup: Hawaiian Islands; island: Kauai; country: United States; stateProvince: Hawaii; county: Kauai; verbatimLocality: Kokee, at falls near tunnel; verbatimElevation: 4000 ft.; **Event:** verbatimEventDate: 6.ix.1946; **Record Level:** institutionCode: BPBM
- **Type status:** Other material. **Occurrence:** recordedBy: EH Bryan Jr.; individualCount: 14; lifeStage: adult; **Location:** islandGroup: Hawaiian Islands; island: Kauai; country: United States; stateProvince: Hawaii; county: Kauai; verbatimLocality: Awaawapuhi; **Identification:** identifiedBy: JA Tenorio; **Event:** verbatimEventDate: 16.vi.1922; **Record Level:** institutionCode: BPBM
- **Type status:** Other material. **Occurrence:** recordedBy: EH Bryan Jr.; individualCount: 6; lifeStage: adult; **Location:** islandGroup: Hawaiian Islands; island: Kauai; country: United States; stateProvince: Hawaii; county: Kauai; verbatimLocality: Honopu; **Identification:** identifiedBy: JA Tenorio; **Event:** verbatimEventDate: 17.vi.1922; **Record Level:** institutionCode: BPBM
- **Type status:** Other material. **Occurrence:** recordedBy: EH Bryan Jr.; individualCount: 4; lifeStage: adult; **Location:** islandGroup: Hawaiian Islands; island: Kauai; country: United States; stateProvince: Hawaii; county: Kauai; verbatimLocality: Kalalau; **Identification:** identifiedBy: JA Tenorio; **Event:** verbatimEventDate: 18.vi.1922; **Record Level:** institutionCode: BPBM
- **Type status:** Other material. **Occurrence:** recordedBy: EH Bryan Jr.; individualCount: 1; lifeStage: adult; **Location:** islandGroup: Hawaiian Islands; island: Kauai; country: United States; stateProvince: Hawaii; county: Kauai; verbatimLocality: Honopu; **Identification:** identifiedBy: JA Tenorio; **Event:** verbatimEventDate: 20.vi.1922; **Record Level:** institutionCode: BPBM
- **Type status:** Other material. **Occurrence:** recordedBy: RL Usinger; individualCount: 3; lifeStage: adult; **Location:** islandGroup: Hawaiian Islands; island: Kauai; country: United States; stateProvince: Hawaii; county: Kauai; verbatimLocality: Kumuwela; **Identification:** identifiedBy: JA Tenorio; **Event:** verbatimEventDate: 30.xi.1935; **Record Level:** institutionCode: BPBM
- **Type status:** Other material. **Occurrence:** recordedBy: EC Zimmerman; individualCount: 23; lifeStage: adult; **Location:** islandGroup: Hawaiian Islands; island: Kauai; country: United States; stateProvince: Hawaii; county: Kauai; verbatimLocality: Near Halemanu; **Identification:** identifiedBy: JA Tenorio; **Event:** verbatimEventDate: 10.vii.1939; **Record Level:** institutionCode: BPBM
- **Type status:** Other material. **Occurrence:** recordedBy: JW Beardsley; individualCount: 3; lifeStage: adult; **Location:** islandGroup: Hawaiian Islands; island: Kauai; country: United States; stateProvince: Hawaii; county: Kauai; verbatimLocality: Kokee, at light trap; **Identification:** identifiedBy: JA Tenorio; **Event:** verbatimEventDate: 24.viii.1959; **Record Level:** institutionCode: BPBM
- **Type status:** Other material. **Occurrence:** recordedBy: NLH Krauss; individualCount: 12; lifeStage: adult; **Location:** islandGroup: Hawaiian Islands; island: Kauai; country: United States; stateProvince: Hawaii; county: Kauai; verbatimLocality: Haena; **Identification:** identifiedBy: JA Tenorio; **Event:** verbatimEventDate: 10.iii.1963; **Record Level:** institutionCode: BPBM
- **Type status:** Other material. **Occurrence:** recordedBy: SL Montgomery; individualCount: 1; lifeStage: adult; **Location:** islandGroup: Hawaiian Islands; island: Kauai; country: United States; stateProvince: Hawaii; county: Kauai; verbatimLocality: Kokee, Honopu; **Identification:** identifiedBy: JA Tenorio; **Event:** verbatimEventDate: 25.xi.1978; **Record Level:** institutionCode: BPBM
- **Type status:** Other material. **Occurrence:** catalogNumber: EMEC7356060; recordedBy: PM O'Grady, RT Lapoint, GM Bennett, BS Ort, NA Pantoja, E Owen; lifeStage: adult; **Location:** islandGroup: Hawaiian Islands; island: Kauai; country: United States; stateProvince: Hawaii; county: Kauai; verbatimLocality: Kokee State Park, Kawaikinana Stream, at first bridge, sweeping stream; verbatimLatitude: 22°7'58.15"N; verbatimLongitude: 159°37'51.67"W; geodeticDatum: WGS84; **Identification:** identifiedBy: PM O'Grady; **Event:** verbatimEventDate: 4.i.2010; **Record Level:** institutionCode: EMEC; collectionCode: 566.2
- **Type status:** Other material. **Occurrence:** catalogNumber: EMEC7356061; recordedBy: PM O'Grady, RT Lapoint, GM Bennett, BS Ort, NA Pantoja, E Owen; lifeStage: adult; **Location:** islandGroup: Hawaiian Islands; island: Kauai; country: United States; stateProvince: Hawaii; county: Kauai; verbatimLocality: Kawaikoi Stream at Sugi Grove; verbatimLatitude: 22°7'53.12"N; verbatimLongitude: 159°37'18.12"W; geodeticDatum: WGS84; **Identification:** identifiedBy: PM O'Grady; **Event:** verbatimEventDate: 4.i.2010; **Record Level:** institutionCode: EMEC; collectionCode: 567.2
- **Type status:** Other material. **Occurrence:** catalogNumber: EMEC7356062; recordedBy: PM O'Grady, RT Lapoint, GM Bennett, BS Ort, NA Pantoja, E Owen; lifeStage: adult; **Location:** islandGroup: Hawaiian Islands; island: Kauai; country: United States; stateProvince: Hawaii; county: Kauai; verbatimLocality: Kawaikoi Stream Trail Sugi Grove to 0.5 miles; verbatimLatitude: 22°7'57.31"N; verbatimLongitude: 159°37'2.13"W; geodeticDatum: WGS84; **Identification:** identifiedBy: PM O'Grady; **Event:** verbatimEventDate: 4.i.2010; **Record Level:** institutionCode: EMEC; collectionCode: 568.1
- **Type status:** Other material. **Occurrence:** catalogNumber: EMEC7356063; recordedBy: PM O'Grady, RT Lapoint, GM Bennett, BS Ort, NA Pantoja, E Owen; individualCount: many; lifeStage: adult; **Location:** islandGroup: Hawaiian Islands; island: Kauai; country: United States; stateProvince: Hawaii; county: Kauai; verbatimLocality: Kohua Ridge Trail, Mohihi Stream, 0-1.0 miles, sweeping stream; verbatimLatitude: 22°6'44.61"N; verbatimLongitude: 159°36'21.69"W; geodeticDatum: WGS84; **Identification:** identifiedBy: PM O'Grady; **Event:** verbatimEventDate: 6.i.2010; **Record Level:** institutionCode: EMEC; collectionCode: 574.2
- **Type status:** Other material. **Occurrence:** catalogNumber: EMEC7356064; recordedBy: PM O'Grady, RT Lapoint, GM Bennett, BS Ort, NA Pantoja, E Owen; lifeStage: adult; **Location:** islandGroup: Hawaiian Islands; island: Kauai; country: United States; stateProvince: Hawaii; county: Kauai; verbatimLocality: Kokee State Park, Mohihi-Waialae Trail @ Mohihi Stream crossing, 0.25miles from parking; verbatimLatitude: 22°7'0.60"N; verbatimLongitude: 159°36'0.31"W; geodeticDatum: WGS84; **Identification:** identifiedBy: PM O'Grady; **Event:** verbatimEventDate: 7.i.2010; **Record Level:** institutionCode: EMEC; collectionCode: 577.1
- **Type status:** Other material. **Occurrence:** catalogNumber: EMEC7356065; recordedBy: PM O'Grady, RT Lapoint, GM Bennett, BS Ort, NA Pantoja; individualCount: many; lifeStage: adult; **Location:** islandGroup: Hawaiian Islands; island: Kauai; country: United States; stateProvince: Hawaii; county: Kauai; verbatimLocality: Upper Kawaikoi Stream @ Alakai Swamp Trail Crossing; verbatimLatitude: 22°8'48.06"N; verbatimLongitude: 159°36'49.38"W; geodeticDatum: WGS84; **Identification:** identifiedBy: PM O'Grady; **Event:** verbatimEventDate: 8.i.2010; **Record Level:** institutionCode: EMEC; collectionCode: 581.1
- **Type status:** Other material. **Occurrence:** catalogNumber: EMEC7356066; recordedBy: PM O'Grady, RT Lapoint, GM Bennett, BS Ort, NA Pantoja; lifeStage: adult; **Location:** islandGroup: Hawaiian Islands; island: Kauai; country: United States; stateProvince: Hawaii; county: Kauai; verbatimLocality: Nualolo Creek, sweeping vegetation in dry creek; verbatimLatitude: 22°8'34.75"N; verbatimLongitude: 159°41'17.74"W; geodeticDatum: WGS84; **Identification:** identifiedBy: PM O'Grady; **Event:** verbatimEventDate: 9.i.2010; **Record Level:** institutionCode: EMEC; collectionCode: 584.2
- **Type status:** Other material. **Occurrence:** catalogNumber: EMEC7356067; recordedBy: PM O'Grady, RT Lapoint, GM Bennett, BS Ort; individualCount: 1; lifeStage: adult; **Location:** islandGroup: Hawaiian Islands; island: Kauai; country: United States; stateProvince: Hawaii; county: Kauai; verbatimLocality: Kokee Stream near Canyon Trail; verbatimLatitude: 22°6'18.56"N; verbatimLongitude: 159°39'41.12"W; geodeticDatum: WGS84; **Identification:** identifiedBy: PM O'Grady; **Event:** verbatimEventDate: 10.i.2010; **Record Level:** institutionCode: EMEC; collectionCode: 588.2
- **Type status:** Other material. **Occurrence:** catalogNumber: EMEC7356068; recordedBy: PM O'Grady, RT Lapoint, GM Bennett, BS Ort; lifeStage: adult; **Location:** islandGroup: Hawaiian Islands; island: Kauai; country: United States; stateProvince: Hawaii; county: Kauai; verbatimLocality: Stream #1, Trail along North Coast; verbatimLatitude: 22°12'40.15"N; verbatimLongitude: 159°35'40.29"W; geodeticDatum: WGS84; **Identification:** identifiedBy: PM O'Grady; **Event:** verbatimEventDate: 11.i.2010; **Record Level:** institutionCode: EMEC; collectionCode: 589.1
- **Type status:** Other material. **Occurrence:** catalogNumber: EMEC7356069; recordedBy: PM O'Grady, RT Lapoint, GM Bennett, BS Ort; lifeStage: adult; **Location:** islandGroup: Hawaiian Islands; island: Kauai; country: United States; stateProvince: Hawaii; county: Kauai; verbatimLocality: Hanapakai Stream, sweeping beach and stream; verbatimLatitude: 22°12'31.40"N; verbatimLongitude: 159°35'51.88"W; geodeticDatum: WGS84; **Identification:** identifiedBy: PM O'Grady; **Event:** verbatimEventDate: 11.i.2010; **Record Level:** institutionCode: EMEC; collectionCode: 590.1
- **Type status:** Other material. **Occurrence:** catalogNumber: EMEC7356070; recordedBy: PM O'Grady, RT Lapoint, GM Bennett, BS Ort; lifeStage: adult; **Location:** islandGroup: Hawaiian Islands; island: Kauai; country: United States; stateProvince: Hawaii; county: Kauai; verbatimLocality: Honoka Trail, seep #1, sweeping cliff face; verbatimLatitude: 22°12'25.22"N; verbatimLongitude: 159°36'1.11"W; geodeticDatum: WGS84; **Identification:** identifiedBy: PM O'Grady; **Event:** verbatimEventDate: 11.i.2010; **Record Level:** institutionCode: EMEC; collectionCode: 591.4
- **Type status:** Other material. **Occurrence:** catalogNumber: EMEC7356071; recordedBy: PM O'Grady, RT Lapoint, GM Bennett, BS Ort; lifeStage: adult; **Location:** islandGroup: Hawaiian Islands; island: Kauai; country: United States; stateProvince: Hawaii; county: Kauai; verbatimLocality: Honokoa Trail, seep #2; verbatimLatitude: 22°12'22.91"N; verbatimLongitude: 159°36'3.96"W; geodeticDatum: WGS84; **Identification:** identifiedBy: PM O'Grady; **Event:** verbatimEventDate: 11.i.2010; **Record Level:** institutionCode: EMEC; collectionCode: 592.2
- **Type status:** Other material. **Occurrence:** catalogNumber: EMEC7356072; recordedBy: PM O'Grady, RT Lapoint, GM Bennett, BS Ort; individualCount: 2; lifeStage: adult; **Location:** islandGroup: Hawaiian Islands; island: Kauai; country: United States; stateProvince: Hawaii; county: Kauai; verbatimLocality: Honokoa Trail, seep #3; verbatimLatitude: 22°12'19.21"N; verbatimLongitude: 159°36'10.24"W; geodeticDatum: WGS84; **Identification:** identifiedBy: PM O'Grady; **Event:** verbatimEventDate: 11.i.2010; **Record Level:** institutionCode: EMEC; collectionCode: 593.1
- **Type status:** Other material. **Occurrence:** catalogNumber: EMEC7356073; recordedBy: PM O'Grady, RT Lapoint, GM Bennett, BS Ort; individualCount: many; lifeStage: adult; **Location:** islandGroup: Hawaiian Islands; island: Kauai; country: United States; stateProvince: Hawaii; county: Kauai; verbatimLocality: Honokoa Trail, seep #4; verbatimLatitude: 22°12'16.43"N; verbatimLongitude: 159°36'12.34"W; geodeticDatum: WGS84; **Identification:** identifiedBy: PM O'Grady; **Event:** verbatimEventDate: 11.i.2010; **Record Level:** institutionCode: EMEC; collectionCode: 594.1

##### Ecological interactions

###### Native status

Endemic.

##### Distribution

Hawaiian Islands: Kauai.

#### Scatella mauiensis

(Wirth, 1948)

##### Materials

- **Type status:** Holotype. **Occurrence:** recordedBy: EH Bryan Jr.; individualCount: 1; sex: male; lifeStage: adult; **Location:** islandGroup: Hawaiian Islands; island: Maui; country: United States; stateProvince: Hawaii; county: Maui; verbatimLocality: Haipuaena; **Event:** verbatimEventDate: 25.vi.1920; **Record Level:** institutionCode: UHM
- **Type status:** Other material. **Occurrence:** recordedBy: LW Quate; individualCount: 1; lifeStage: adult; **Location:** islandGroup: Hawaiian Islands; island: Maui; country: United States; stateProvince: Hawaii; county: Maui; verbatimLocality: Waikamoi; verbatimElevation: 3936 ft.; **Event:** verbatimEventDate: 14.iii.1961; **Record Level:** institutionCode: BPBM
- **Type status:** Other material. **Occurrence:** recordedBy: JL Gressitt; individualCount: 2; lifeStage: adult; **Location:** islandGroup: Hawaiian Islands; island: Molokai; country: United States; stateProvince: Hawaii; county: Maui; verbatimLocality: Halawa Valley; **Identification:** identifiedBy: K Arakaki; **Event:** verbatimEventDate: 23.iii.1970; **Record Level:** institutionCode: BPBM
- **Type status:** Other material. **Occurrence:** recordedBy: WC Gagne; individualCount: 4; lifeStage: adult; **Location:** islandGroup: Hawaiian Islands; island: Hawaii; country: United States; stateProvince: Hawaii; county: Hawaii; verbatimLocality: Kohala Mountains, Honokane Nui Stream and branch; **Identification:** identifiedBy: JA Tenorio; **Event:** verbatimEventDate: 13.vi.1970; **Record Level:** institutionCode: BPBM
- **Type status:** Other material. **Occurrence:** recordedBy: WC Gagne; individualCount: 1; lifeStage: adult; **Location:** islandGroup: Hawaiian Islands; island: Maui; country: United States; stateProvince: Hawaii; county: Maui; verbatimLocality: Haleakala National Park, Kipahulu Valley, ridge E. of Greensword Bog; verbatimElevation: 5947 ft.; **Identification:** identifiedBy: K Arakaki; **Event:** verbatimEventDate: 25.vi.1981; **Record Level:** institutionCode: BPBM
- **Type status:** Other material. **Occurrence:** recordedBy: GM Nishida; individualCount: 24; lifeStage: adult; **Location:** islandGroup: Hawaiian Islands; island: Maui; country: United States; stateProvince: Hawaii; county: Maui; verbatimLocality: E. Hahalawe Gulch, in waterfall spray zone; verbatimElevation: 1197-1345 ft.; **Identification:** identifiedBy: K Arakaki; **Event:** verbatimEventDate: 5.v.1984; **Record Level:** institutionCode: BPBM
- **Type status:** Other material. **Occurrence:** catalogNumber: EMEC7356074; recordedBy: PM O'Grady, RT Lapoint, GM Bennett, BS Ort, NA Pantoja; lifeStage: adult; **Location:** islandGroup: Hawaiian Islands; island: Hawaii; country: United States; stateProvince: Hawaii; county: Hawaii; verbatimLocality: Kolekole Beach Park, sweeping along ocean coast; verbatimLatitude: 19°52'57.93"N; verbatimLongitude: 155°7'9.22"W; geodeticDatum: WGS84; **Identification:** identifiedBy: PM O'Grady; **Event:** verbatimEventDate: 2.viii.2010; **Record Level:** institutionCode: EMEC; collectionCode: 616.5

##### Ecological interactions

###### Native status

Endemic.

##### Distribution

Hawaiian Islands: Molokai, Maui, Hawaii.

#### Scatella oahuense

Williams, 1938

##### Materials

- **Type status:** Holotype. **Occurrence:** catalogNumber: 4245; recordedBy: FX Williams; individualCount: 1; sex: female; lifeStage: adult; **Location:** islandGroup: Hawaiian Islands; island: Oahu; country: United States; stateProvince: Hawaii; county: Honolulu; verbatimLocality: Hering Valley, Tantalus, wet bank; **Event:** verbatimEventDate: 6.iii.1937; **Record Level:** institutionCode: BPBM
- **Type status:** Paratype. **Occurrence:** catalogNumber: 4245a; recordedBy: FX Williams; individualCount: 1; sex: male; lifeStage: adult; **Location:** islandGroup: Hawaiian Islands; island: Oahu; country: United States; stateProvince: Hawaii; county: Honolulu; verbatimLocality: Hering Valley, Tantalus, wet bank; **Event:** verbatimEventDate: 6.iii.1937; **Record Level:** institutionCode: BPBM
- **Type status:** Paratype. **Occurrence:** recordedBy: FX Williams; lifeStage: adult; **Location:** islandGroup: Hawaiian Islands; island: Oahu; country: United States; stateProvince: Hawaii; county: Honolulu; verbatimLocality: Tantalus; **Event:** verbatimEventDate: 1936-1937; **Record Level:** institutionCode: UHM
- **Type status:** Paratype. **Occurrence:** recordedBy: FX Williams; lifeStage: adult; **Location:** islandGroup: Hawaiian Islands; island: Oahu; country: United States; stateProvince: Hawaii; county: Honolulu; verbatimLocality: Upper Manoa Valley; **Event:** verbatimEventDate: 1936-1937; **Record Level:** institutionCode: UHM
- **Type status:** Paratype. **Occurrence:** recordedBy: FX Williams; lifeStage: adult; **Location:** islandGroup: Hawaiian Islands; island: Oahu; country: United States; stateProvince: Hawaii; county: Honolulu; verbatimLocality: Honolulu; **Event:** verbatimEventDate: 1936-1937; **Record Level:** institutionCode: UHM
- **Type status:** Paratype. **Occurrence:** recordedBy: FX Williams; lifeStage: adult; **Location:** islandGroup: Hawaiian Islands; island: Oahu; country: United States; stateProvince: Hawaii; county: Honolulu; verbatimLocality: Waikane; **Event:** verbatimEventDate: 1936-1937; **Record Level:** institutionCode: UHM
- **Type status:** Paratype. **Occurrence:** recordedBy: FX Williams; lifeStage: adult; **Location:** islandGroup: Hawaiian Islands; island: Oahu; country: United States; stateProvince: Hawaii; county: Honolulu; verbatimLocality: Kaluanui Valley; **Event:** verbatimEventDate: 1936-1937; **Record Level:** institutionCode: UHM
- **Type status:** Paratype. **Occurrence:** recordedBy: FX Williams; lifeStage: adult; **Location:** islandGroup: Hawaiian Islands; island: Oahu; country: United States; stateProvince: Hawaii; county: Honolulu; verbatimLocality: Mt. Kaala; **Event:** verbatimEventDate: 1936-1937; **Record Level:** institutionCode: UHM
- **Type status:** Other material. **Occurrence:** recordedBy: JF Illingworth; individualCount: 11; lifeStage: adult; **Location:** islandGroup: Hawaiian Islands; island: Oahu; country: United States; stateProvince: Hawaii; county: Honolulu; verbatimLocality: Palolo Falls; **Event:** verbatimEventDate: vii.1916; **Record Level:** institutionCode: UHM
- **Type status:** Other material. **Occurrence:** recordedBy: JF Illingworth; individualCount: 7; lifeStage: adult; **Location:** islandGroup: Hawaiian Islands; island: Oahu; country: United States; stateProvince: Hawaii; county: Honolulu; verbatimLocality: Palolo Falls; **Identification:** identifiedBy: WW Wirth; **Event:** verbatimEventDate: vii.1916; **Record Level:** institutionCode: BPBM
- **Type status:** Other material. **Occurrence:** recordedBy: JC Bridwell; individualCount: 1; lifeStage: adult; **Location:** islandGroup: Hawaiian Islands; island: Oahu; country: United States; stateProvince: Hawaii; county: Honolulu; verbatimLocality: Lanihuli R.; **Identification:** identifiedBy: WW Wirth; **Event:** verbatimEventDate: 3.ix.1916; **Record Level:** institutionCode: BPBM
- **Type status:** Other material. **Occurrence:** recordedBy: Timberlake; individualCount: 1; lifeStage: adult; **Location:** islandGroup: Hawaiian Islands; island: Maui; country: United States; stateProvince: Hawaii; county: Maui; verbatimLocality: Puu Nianiau; **Identification:** identifiedBy: JA Tenorio; **Event:** verbatimEventDate: 16.vii.1919; **Record Level:** institutionCode: BPBM
- **Type status:** Other material. **Occurrence:** recordedBy: EH Bryan Jr.; individualCount: 1; lifeStage: adult; **Location:** islandGroup: Hawaiian Islands; island: Maui; country: United States; stateProvince: Hawaii; county: Maui; verbatimLocality: Haipuaena; **Identification:** identifiedBy: JA Tenorio; **Event:** verbatimEventDate: 25.vi.1920; **Record Level:** institutionCode: BPBM
- **Type status:** Other material. **Occurrence:** recordedBy: OH Swezey; individualCount: 4; sex: male; lifeStage: adult; **Location:** islandGroup: Hawaiian Islands; island: Oahu; country: United States; stateProvince: Hawaii; county: Honolulu; verbatimLocality: Niu; **Event:** verbatimEventDate: 13.i.1924; **Record Level:** institutionCode: UHM
- **Type status:** Other material. **Occurrence:** recordedBy: OH Swezey; individualCount: 1; lifeStage: adult; **Location:** islandGroup: Hawaiian Islands; island: Oahu; country: United States; stateProvince: Hawaii; county: Honolulu; verbatimLocality: Niu; **Identification:** identifiedBy: WW Wirth; **Event:** verbatimEventDate: 13.i.1924; **Record Level:** institutionCode: BPBM
- **Type status:** Other material. **Occurrence:** recordedBy: NLH Krauss; individualCount: 1; lifeStage: adult; **Location:** islandGroup: Hawaiian Islands; island: Lanai; country: United States; stateProvince: Hawaii; county: Maui; verbatimLocality: Lanaihale; **Identification:** identifiedBy: JA Tenorio; **Event:** verbatimEventDate: 17.ii.1965; **Record Level:** institutionCode: BPBM
- **Type status:** Other material. **Occurrence:** recordedBy: EH Bryan Jr.; individualCount: 2; lifeStage: adult; **Location:** islandGroup: Hawaiian Islands; island: Oahu; country: United States; stateProvince: Hawaii; county: Honolulu; verbatimLocality: Makaleha; **Identification:** identifiedBy: WW Wirth; **Event:** verbatimEventDate: 13.i.1929; **Record Level:** institutionCode: BPBM
- **Type status:** Other material. **Occurrence:** recordedBy: O Bryant; individualCount: 2; lifeStage: adult; **Location:** islandGroup: Hawaiian Islands; island: Maui; country: United States; stateProvince: Hawaii; county: Maui; verbatimLocality: Haleakala Crater; verbatimElevation: 7000; **Identification:** identifiedBy: JA Tenorio; **Event:** verbatimEventDate: 23.iii.1932; **Record Level:** institutionCode: BPBM
- **Type status:** Other material. **Occurrence:** recordedBy: EH Bryan Jr.; individualCount: 21; lifeStage: adult; **Location:** islandGroup: Hawaiian Islands; island: Oahu; country: United States; stateProvince: Hawaii; county: Honolulu; verbatimLocality: Kamokuiki Valley; **Identification:** identifiedBy: WW Wirth; **Event:** verbatimEventDate: 13.iv.1933; **Record Level:** institutionCode: BPBM
- **Type status:** Other material. **Occurrence:** recordedBy: EH Bryan Jr.; individualCount: 3; lifeStage: adult; **Location:** islandGroup: Hawaiian Islands; island: Oahu; country: United States; stateProvince: Hawaii; county: Honolulu; verbatimLocality: Castle trail; **Identification:** identifiedBy: WW Wirth; **Event:** verbatimEventDate: 14.x.1934; **Record Level:** institutionCode: BPBM
- **Type status:** Other material. **Occurrence:** recordedBy: EC Zimmerman; individualCount: 1; lifeStage: adult; **Location:** islandGroup: Hawaiian Islands; island: Oahu; country: United States; stateProvince: Hawaii; county: Honolulu; verbatimLocality: Mt. Tantalus; **Identification:** identifiedBy: WW Wirth; **Event:** verbatimEventDate: 23.i.1937; **Record Level:** institutionCode: BPBM
- **Type status:** Other material. **Occurrence:** recordedBy: WW Wirth; individualCount: 3; lifeStage: adult; **Location:** islandGroup: Hawaiian Islands; island: Oahu; country: United States; stateProvince: Hawaii; county: Honolulu; verbatimLocality: Manoa Valley; **Event:** verbatimEventDate: 6.v.1945; **Record Level:** institutionCode: BPBM
- **Type status:** Other material. **Occurrence:** catalogNumber: USNMENT 00972758; recordedBy: WW Wirth; individualCount: 1; sex: male; lifeStage: adult; **Location:** islandGroup: Hawaiian Islands; island: Oahu; country: United States; stateProvince: Hawaii; county: Honolulu; verbatimLocality: Manoa Valley; **Event:** verbatimEventDate: 6.v.1945; **Record Level:** institutionCode: USNM
- **Type status:** Other material. **Occurrence:** catalogNumber: USNMENT 00972759; recordedBy: WW Wirth; individualCount: 1; sex: male; lifeStage: adult; **Location:** islandGroup: Hawaiian Islands; island: Oahu; country: United States; stateProvince: Hawaii; county: Honolulu; verbatimLocality: Manoa Valley; **Event:** verbatimEventDate: 6.v.1945; **Record Level:** institutionCode: USNM
- **Type status:** Other material. **Occurrence:** catalogNumber: USNMENT 00972760; recordedBy: WW Wirth; individualCount: 1; sex: male; lifeStage: adult; **Location:** islandGroup: Hawaiian Islands; island: Oahu; country: United States; stateProvince: Hawaii; county: Honolulu; verbatimLocality: Manoa Valley; **Event:** verbatimEventDate: 6.v.1945; **Record Level:** institutionCode: USNM
- **Type status:** Other material. **Occurrence:** recordedBy: WW Wirth; individualCount: 1; sex: male; lifeStage: adult; **Location:** islandGroup: Hawaiian Islands; island: Oahu; country: United States; stateProvince: Hawaii; county: Honolulu; verbatimLocality: Manoa Valley, on moist rocks in stream; **Event:** verbatimEventDate: 19.i.1946; **Record Level:** institutionCode: BPBM
- **Type status:** Other material. **Occurrence:** recordedBy: Y Kondo; individualCount: 1; lifeStage: adult; **Location:** islandGroup: Hawaiian Islands; island: Oahu; country: United States; stateProvince: Hawaii; county: Honolulu; verbatimLocality: Laulaupoe Gulch; **Identification:** identifiedBy: WW Wirth; **Event:** verbatimEventDate: 9.vii.1951; **Record Level:** institutionCode: BPBM
- **Type status:** Other material. **Occurrence:** recordedBy: CP Hoyt; individualCount: 6; lifeStage: adult; **Location:** islandGroup: Hawaiian Islands; island: Oahu; country: United States; stateProvince: Hawaii; county: Honolulu; verbatimLocality: Maunawili; verbatimElevation: 1200; **Identification:** identifiedBy: JA Tenorio; **Event:** verbatimEventDate: 31.i.1953; **Record Level:** institutionCode: BPBM
- **Type status:** Other material. **Occurrence:** recordedBy: CP Hoyt; individualCount: 5; lifeStage: adult; **Location:** islandGroup: Hawaiian Islands; island: Oahu; country: United States; stateProvince: Hawaii; county: Honolulu; verbatimLocality: Kuliouou; verbatimElevation: 1500; **Identification:** identifiedBy: JA Tenorio; **Event:** verbatimEventDate: 7.ii.1953; **Record Level:** institutionCode: BPBM
- **Type status:** Other material. **Occurrence:** recordedBy: CP Hoyt; individualCount: 1; lifeStage: adult; **Location:** islandGroup: Hawaiian Islands; island: Oahu; country: United States; stateProvince: Hawaii; county: Honolulu; verbatimLocality: Mt. Kaala; **Identification:** identifiedBy: JA Tenorio; **Event:** verbatimEventDate: 3.iv.1953; **Record Level:** institutionCode: BPBM
- **Type status:** Other material. **Occurrence:** recordedBy: CA Isenberg; individualCount: 2; lifeStage: adult; **Location:** islandGroup: Hawaiian Islands; island: Kauai; country: United States; stateProvince: Hawaii; county: Kauai; verbatimLocality: Wailua; **Identification:** identifiedBy: JA Tenorio; **Event:** verbatimEventDate: xii.1956; **Record Level:** institutionCode: BPBM
- **Type status:** Other material. **Occurrence:** recordedBy: NLH Krauss; individualCount: 2; lifeStage: adult; **Location:** islandGroup: Hawaiian Islands; island: Maui; country: United States; stateProvince: Hawaii; county: Maui; verbatimLocality: Ulupalakua; **Identification:** identifiedBy: JA Tenorio; **Event:** verbatimEventDate: 8.ii.1958; **Record Level:** institutionCode: BPBM
- **Type status:** Other material. **Occurrence:** recordedBy: LW Quate; individualCount: 2; lifeStage: adult; **Location:** islandGroup: Hawaiian Islands; island: Hawaii; country: United States; stateProvince: Hawaii; county: Hawaii; verbatimLocality: Head of Waipio Valley; verbatimElevation: 3936; **Identification:** identifiedBy: JA Tenorio; **Event:** verbatimEventDate: 23.iii.1961; **Record Level:** institutionCode: BPBM
- **Type status:** Other material. **Occurrence:** recordedBy: RCA Rice; individualCount: 15; lifeStage: adult; **Location:** islandGroup: Hawaiian Islands; island: Maui; country: United States; stateProvince: Hawaii; county: Maui; verbatimLocality: Haleakala National Park, Kaupo Gap; verbatimElevation: 5400; **Identification:** identifiedBy: K Arakaki; **Event:** verbatimEventDate: 24.vi.1976; **Record Level:** institutionCode: BPBM
- **Type status:** Other material. **Occurrence:** recordedBy: WC Gagne; individualCount: 3; lifeStage: adult; **Location:** islandGroup: Hawaiian Islands; island: Maui; country: United States; stateProvince: Hawaii; county: Maui; verbatimLocality: Haleakala National Park, Kipahulu Valley, ridge E. of Greensword Bog; verbatimElevation: 5947; **Identification:** identifiedBy: K Arakaki; **Event:** verbatimEventDate: 25.vii.1981; **Record Level:** institutionCode: BPBM

##### Ecological interactions

###### Native status

Endemic.

##### Distribution

Hawaiian Islands: Oahu, Lanai, Maui, Hawaii.

##### Notes

12 specimens from USNM not examined.

#### Scatella sexnotata

Cresson, 1926

##### Materials

- **Type status:** Holotype. **Occurrence:** catalogNumber: 352; recordedBy: FW Terry; individualCount: 1; sex: male; lifeStage: adult; **Location:** islandGroup: Hawaiian Islands; island: Oahu; country: United States; stateProvince: Hawaii; county: Honolulu; verbatimLocality: Waimanalo; **Event:** verbatimEventDate: 11.vi.1907; **Record Level:** institutionCode: BPBM
- **Type status:** Paratype. **Occurrence:** catalogNumber: 352a; recordedBy: FW Terry; individualCount: 1; sex: female; lifeStage: adult; **Location:** islandGroup: Hawaiian Islands; island: Oahu; country: United States; stateProvince: Hawaii; county: Honolulu; verbatimLocality: Waimanalo; **Event:** verbatimEventDate: 11.vi.1907; **Record Level:** institutionCode: BPBM
- **Type status:** Paratype. **Occurrence:** recordedBy: FW Terry; individualCount: 5; lifeStage: adult; **Location:** islandGroup: Hawaiian Islands; island: Oahu; country: United States; stateProvince: Hawaii; county: Honolulu; verbatimLocality: Waimanalo; **Event:** verbatimEventDate: 11.vi.1907; **Record Level:** institutionCode: UHM
- **Type status:** Paratype. **Occurrence:** recordedBy: FW Terry; individualCount: 3; lifeStage: adult; **Location:** islandGroup: Hawaiian Islands; island: Oahu; country: United States; stateProvince: Hawaii; county: Honolulu; verbatimLocality: Waimanalo; **Identification:** identifiedBy: ET Cresson; **Event:** verbatimEventDate: 11.vii.1907; **Record Level:** institutionCode: BPBM
- **Type status:** Other material. **Occurrence:** recordedBy: no collector given; individualCount: 1; lifeStage: adult; **Location:** islandGroup: Hawaiian Islands; island: Oahu; country: United States; stateProvince: Hawaii; county: Honolulu; verbatimLocality: Honolulu; **Event:** verbatimEventDate: no date given; **Record Level:** institutionCode: BPBM
- **Type status:** Other material. **Occurrence:** recordedBy: RCL Perkins; individualCount: 5; lifeStage: adult; **Location:** islandGroup: Hawaiian Islands; island: Oahu; country: United States; stateProvince: Hawaii; county: Honolulu; verbatimLocality: Honolulu; **Event:** verbatimEventDate: no date given; **Record Level:** institutionCode: BPBM
- **Type status:** Other material. **Occurrence:** recordedBy: RCL Perkins; individualCount: 1; lifeStage: adult; **Location:** islandGroup: Hawaiian Islands; island: Laysan; country: United States; stateProvince: Hawaii; **Event:** verbatimEventDate: no date given; **Record Level:** institutionCode: BPBM
- **Type status:** Other material. **Occurrence:** recordedBy: GP Wilder; individualCount: 38; lifeStage: adult; **Location:** islandGroup: Hawaiian Islands; island: Laysan; country: United States; stateProvince: Hawaii; **Event:** verbatimEventDate: no date given; **Record Level:** institutionCode: BPBM
- **Type status:** Other material. **Occurrence:** recordedBy: JF Illingworth; individualCount: 1; lifeStage: adult; **Location:** islandGroup: Hawaiian Islands; island: Oahu; country: United States; stateProvince: Hawaii; county: Honolulu; **Event:** verbatimEventDate: 7.i.1913; **Record Level:** institutionCode: BPBM
- **Type status:** Other material. **Occurrence:** recordedBy: JF Illingworth; individualCount: 5; lifeStage: adult; **Location:** islandGroup: Hawaiian Islands; island: Oahu; country: United States; stateProvince: Hawaii; county: Honolulu; **Event:** verbatimEventDate: ix.1914; **Record Level:** institutionCode: BPBM
- **Type status:** Other material. **Occurrence:** recordedBy: GP Wilder; individualCount: 6; lifeStage: adult; **Location:** islandGroup: Hawaiian Islands; island: Laysan; country: United States; stateProvince: Hawaii; **Event:** verbatimEventDate: viii.1920; **Record Level:** institutionCode: BPBM
- **Type status:** Other material. **Occurrence:** recordedBy: EH Bryan Jr.; individualCount: 16; lifeStage: adult; **Location:** islandGroup: Hawaiian Islands; island: Maui; country: United States; stateProvince: Hawaii; county: Maui; verbatimLocality: Kahalui Swamp; **Event:** verbatimEventDate: 7.vii.1920; **Record Level:** institutionCode: BPBM
- **Type status:** Other material. **Occurrence:** recordedBy: EH Bryan Jr.; individualCount: 1; lifeStage: adult; **Location:** islandGroup: Hawaiian Islands; island: Oahu; country: United States; stateProvince: Hawaii; county: Honolulu; verbatimLocality: Ewa Coral Plain; **Event:** verbatimEventDate: 29.i.1922; **Record Level:** institutionCode: BPBM
- **Type status:** Other material. **Occurrence:** recordedBy: EH Bryan Jr.; individualCount: 2; lifeStage: adult; **Location:** islandGroup: Hawaiian Islands; island: Oahu; country: United States; stateProvince: Hawaii; county: Honolulu; verbatimLocality: Honolulu; **Event:** verbatimEventDate: 2.iv.1923; **Record Level:** institutionCode: BPBM
- **Type status:** Other material. **Occurrence:** recordedBy: DT Fullaway; individualCount: 21; lifeStage: adult; **Location:** islandGroup: Hawaiian Islands; island: Laysan; country: United States; stateProvince: Hawaii; **Event:** verbatimEventDate: 8-11.iv.1923; **Record Level:** institutionCode: BPBM
- **Type status:** Other material. **Occurrence:** recordedBy: EH Bryan Jr.; individualCount: 11; lifeStage: adult; **Location:** islandGroup: Hawaiian Islands; island: Nihoa; country: United States; stateProvince: Hawaii; county: Nihoa; verbatimLocality: stagnant water; **Event:** verbatimEventDate: 13.vi.1923; **Record Level:** institutionCode: BPBM
- **Type status:** Other material. **Occurrence:** recordedBy: EH Bryan Jr.; individualCount: 17; lifeStage: adult; **Location:** islandGroup: Hawaiian Islands; island: Necker; country: United States; stateProvince: Hawaii; verbatimLocality: pools of salt water; **Event:** verbatimEventDate: 18.vi.1923; **Record Level:** institutionCode: BPBM
- **Type status:** Other material. **Occurrence:** recordedBy: EH Bryan Jr.; individualCount: 4; lifeStage: adult; **Location:** islandGroup: Hawaiian Islands; island: Necker; country: United States; stateProvince: Hawaii; **Event:** verbatimEventDate: 29.vi.1923; **Record Level:** institutionCode: BPBM
- **Type status:** Other material. **Occurrence:** recordedBy: O Bryant; individualCount: 1; lifeStage: adult; **Location:** islandGroup: Hawaiian Islands; island: Oahu; country: United States; stateProvince: Hawaii; county: Honolulu; verbatimLocality: Waikiki; **Event:** verbatimEventDate: 1.iii.1932; **Record Level:** institutionCode: BPBM
- **Type status:** Other material. **Occurrence:** recordedBy: EH Bryan Jr.; individualCount: 37; lifeStage: adult; **Location:** islandGroup: Hawaiian Islands; island: Oahu; country: United States; stateProvince: Hawaii; county: Honolulu; verbatimLocality: Maunalua; **Event:** verbatimEventDate: 19.iv.1937; **Record Level:** institutionCode: BPBM
- **Type status:** Other material. **Occurrence:** catalogNumber: USNMENT 00972761; recordedBy: WW Wirth; individualCount: 1; sex: male; lifeStage: adult; **Location:** islandGroup: Hawaiian Islands; island: Oahu; country: United States; stateProvince: Hawaii; county: Honolulu; verbatimLocality: Waimea, on rocks by sea; **Event:** verbatimEventDate: 31.i.1946; **Record Level:** institutionCode: USNM
- **Type status:** Other material. **Occurrence:** catalogNumber: USNMENT 00972762; recordedBy: WW Wirth; individualCount: 1; sex: male; lifeStage: adult; **Location:** islandGroup: Hawaiian Islands; island: Oahu; country: United States; stateProvince: Hawaii; county: Honolulu; verbatimLocality: Waimea, on rocks by sea; **Event:** verbatimEventDate: 31.i.1946; **Record Level:** institutionCode: USNM
- **Type status:** Other material. **Occurrence:** recordedBy: WW Wirth; individualCount: 1; lifeStage: adult; **Location:** islandGroup: Hawaiian Islands; island: Oahu; country: United States; stateProvince: Hawaii; county: Honolulu; verbatimLocality: Kalihi, light trap; **Event:** verbatimEventDate: ii.1946; **Record Level:** institutionCode: UHM
- **Type status:** Other material. **Occurrence:** catalogNumber: USNMENT 00972763; recordedBy: WW Wirth; individualCount: 1; sex: male; lifeStage: adult; **Location:** islandGroup: Hawaiian Islands; island: Oahu; country: United States; stateProvince: Hawaii; county: Honolulu; verbatimLocality: Kalihi, light trap; **Event:** verbatimEventDate: ii.1946; **Record Level:** institutionCode: USNM
- **Type status:** Other material. **Occurrence:** catalogNumber: USNMENT 00972764; recordedBy: WW Wirth; individualCount: 1; sex: female; lifeStage: adult; **Location:** islandGroup: Hawaiian Islands; island: Oahu; country: United States; stateProvince: Hawaii; county: Honolulu; verbatimLocality: Kalihi, light trap; **Event:** verbatimEventDate: ii.1946; **Record Level:** institutionCode: USNM
- **Type status:** Other material. **Occurrence:** catalogNumber: USNMENT 00972765; recordedBy: WW Wirth; individualCount: 1; sex: ?; lifeStage: adult; **Location:** islandGroup: Hawaiian Islands; island: Oahu; country: United States; stateProvince: Hawaii; county: Honolulu; verbatimLocality: Kalihi, light trap; **Event:** verbatimEventDate: ii.1946; **Record Level:** institutionCode: USNM
- **Type status:** Other material. **Occurrence:** catalogNumber: USNMENT 00972766; recordedBy: WW Wirth; individualCount: 1; sex: female; lifeStage: adult; **Location:** islandGroup: Hawaiian Islands; island: Oahu; country: United States; stateProvince: Hawaii; county: Honolulu; verbatimLocality: Kalihi, light trap; **Event:** verbatimEventDate: ii.1946; **Record Level:** institutionCode: USNM
- **Type status:** Other material. **Occurrence:** catalogNumber: USNMENT 00972767; recordedBy: WW Wirth; individualCount: 1; sex: male; lifeStage: adult; **Location:** islandGroup: Hawaiian Islands; island: Oahu; country: United States; stateProvince: Hawaii; county: Honolulu; verbatimLocality: Kalihi, light trap; **Event:** verbatimEventDate: ii.1946; **Record Level:** institutionCode: USNM
- **Type status:** Other material. **Occurrence:** catalogNumber: USNMENT 00972768; recordedBy: WW Wirth; individualCount: 1; sex: male; lifeStage: adult; **Location:** islandGroup: Hawaiian Islands; island: Oahu; country: United States; stateProvince: Hawaii; county: Honolulu; verbatimLocality: Kalihi, light trap; **Event:** verbatimEventDate: ii.1946; **Record Level:** institutionCode: USNM
- **Type status:** Other material. **Occurrence:** catalogNumber: USNMENT 00972769; recordedBy: WW Wirth; individualCount: 1; sex: male; lifeStage: adult; **Location:** islandGroup: Hawaiian Islands; island: Oahu; country: United States; stateProvince: Hawaii; county: Honolulu; verbatimLocality: Kalihi, light trap; **Event:** verbatimEventDate: ii.1946; **Record Level:** institutionCode: USNM
- **Type status:** Other material. **Occurrence:** catalogNumber: USNMENT 00972770; recordedBy: WW Wirth; individualCount: 1; sex: male; lifeStage: adult; **Location:** islandGroup: Hawaiian Islands; island: Oahu; country: United States; stateProvince: Hawaii; county: Honolulu; verbatimLocality: Kalihi, light trap; **Event:** verbatimEventDate: ii.1946; **Record Level:** institutionCode: USNM
- **Type status:** Other material. **Occurrence:** catalogNumber: USNMENT 00972771; recordedBy: WW Wirth; individualCount: 1; sex: male; lifeStage: adult; **Location:** islandGroup: Hawaiian Islands; island: Oahu; country: United States; stateProvince: Hawaii; county: Honolulu; verbatimLocality: Kalihi, light trap; **Event:** verbatimEventDate: ii.1946; **Record Level:** institutionCode: USNM
- **Type status:** Other material. **Occurrence:** catalogNumber: USNMENT 00972772; recordedBy: WW Wirth; individualCount: 1; sex: female; lifeStage: adult; **Location:** islandGroup: Hawaiian Islands; island: Oahu; country: United States; stateProvince: Hawaii; county: Honolulu; verbatimLocality: Kalihi, light trap; **Event:** verbatimEventDate: ii.1946; **Record Level:** institutionCode: USNM
- **Type status:** Other material. **Occurrence:** recordedBy: no collector given; individualCount: 2; lifeStage: adult; **Location:** islandGroup: Hawaiian Islands; island: Oahu; country: United States; stateProvince: Hawaii; county: Honolulu; verbatimLocality: Waipio, at light trap; **Event:** verbatimEventDate: 29.i-4.ii.1946; **Record Level:** institutionCode: BPBM
- **Type status:** Other material. **Occurrence:** recordedBy: WW Wirth; individualCount: 1; lifeStage: adult; **Location:** islandGroup: Hawaiian Islands; island: Oahu; country: United States; stateProvince: Hawaii; county: Honolulu; verbatimLocality: Kahuku, light trap; **Event:** verbatimEventDate: 1.ii.1946; **Record Level:** institutionCode: UHM
- **Type status:** Other material. **Occurrence:** catalogNumber: USNMENT 00972773; recordedBy: WW Wirth; individualCount: 1; sex: female; lifeStage: adult; **Location:** islandGroup: Hawaiian Islands; island: Oahu; country: United States; stateProvince: Hawaii; county: Honolulu; verbatimLocality: Kahuku, light trap; **Event:** verbatimEventDate: 1.ii.1946; **Record Level:** institutionCode: USNM
- **Type status:** Other material. **Occurrence:** catalogNumber: USNMENT 00972774; recordedBy: WW Wirth; individualCount: 1; sex: female; lifeStage: adult; **Location:** islandGroup: Hawaiian Islands; island: Oahu; country: United States; stateProvince: Hawaii; county: Honolulu; verbatimLocality: Kahuku, light trap; **Event:** verbatimEventDate: 1.ii.1946; **Record Level:** institutionCode: USNM
- **Type status:** Other material. **Occurrence:** catalogNumber: USNMENT 00972775; recordedBy: WW Wirth; individualCount: 1; sex: female; lifeStage: adult; **Location:** islandGroup: Hawaiian Islands; island: Oahu; country: United States; stateProvince: Hawaii; county: Honolulu; verbatimLocality: Kahuku, light trap; **Event:** verbatimEventDate: 1.ii.1946; **Record Level:** institutionCode: USNM
- **Type status:** Other material. **Occurrence:** catalogNumber: USNMENT 00972776; recordedBy: WW Wirth; individualCount: 1; sex: female; lifeStage: adult; **Location:** islandGroup: Hawaiian Islands; island: Oahu; country: United States; stateProvince: Hawaii; county: Honolulu; verbatimLocality: Kahuku, light trap; **Event:** verbatimEventDate: 1.ii.1946; **Record Level:** institutionCode: USNM
- **Type status:** Other material. **Occurrence:** catalogNumber: USNMENT 00972777; recordedBy: WW Wirth; individualCount: 1; sex: male; lifeStage: adult; **Location:** islandGroup: Hawaiian Islands; island: Oahu; country: United States; stateProvince: Hawaii; county: Honolulu; verbatimLocality: Kahuku, light trap; **Event:** verbatimEventDate: 1.ii.1946; **Record Level:** institutionCode: USNM
- **Type status:** Other material. **Occurrence:** recordedBy: WW Wirth; individualCount: 1; sex: female; lifeStage: adult; **Location:** islandGroup: Hawaiian Islands; island: Oahu; country: United States; stateProvince: Hawaii; county: Honolulu; verbatimLocality: Kaneohe, marshy bay shore; **Event:** verbatimEventDate: 13.iii.1946; **Record Level:** institutionCode: UHM
- **Type status:** Other material. **Occurrence:** catalogNumber: USNMENT 00972778; recordedBy: WW Wirth; individualCount: 1; sex: female; lifeStage: adult; **Location:** islandGroup: Hawaiian Islands; island: Oahu; country: United States; stateProvince: Hawaii; county: Honolulu; verbatimLocality: Kaneohe, marshy bay shore; **Event:** verbatimEventDate: 13.iii.1946; **Record Level:** institutionCode: USNM
- **Type status:** Other material. **Occurrence:** catalogNumber: USNMENT 00972779; recordedBy: WW Wirth; individualCount: 1; sex: female; lifeStage: adult; **Location:** islandGroup: Hawaiian Islands; island: Oahu; country: United States; stateProvince: Hawaii; county: Honolulu; verbatimLocality: Kaneohe, marshy bay shore; **Event:** verbatimEventDate: 13.iii.1946; **Record Level:** institutionCode: USNM
- **Type status:** Other material. **Occurrence:** recordedBy: WW Wirth; individualCount: 5; lifeStage: adult; **Location:** islandGroup: Hawaiian Islands; island: Oahu; country: United States; stateProvince: Hawaii; county: Honolulu; verbatimLocality: Ewa, on stranded seaweed on beach; **Event:** verbatimEventDate: 14.iii.1946; **Record Level:** institutionCode: UHM
- **Type status:** Other material. **Occurrence:** catalogNumber: USNMENT 00972781; recordedBy: WW Wirth; individualCount: 1; sex: female; lifeStage: adult; **Location:** islandGroup: Hawaiian Islands; island: Oahu; country: United States; stateProvince: Hawaii; county: Honolulu; verbatimLocality: Ewa, on stranded seaweed on beach; **Event:** verbatimEventDate: 14.iii.1946; **Record Level:** institutionCode: USNM
- **Type status:** Other material. **Occurrence:** catalogNumber: USNMENT 00972782; recordedBy: WW Wirth; individualCount: 1; sex: female; lifeStage: adult; **Location:** islandGroup: Hawaiian Islands; island: Oahu; country: United States; stateProvince: Hawaii; county: Honolulu; verbatimLocality: Ewa, on stranded seaweed on beach; **Event:** verbatimEventDate: 14.iii.1946; **Record Level:** institutionCode: USNM
- **Type status:** Other material. **Occurrence:** catalogNumber: USNMENT 00972783; recordedBy: WW Wirth; individualCount: 1; sex: female; lifeStage: adult; **Location:** islandGroup: Hawaiian Islands; island: Oahu; country: United States; stateProvince: Hawaii; county: Honolulu; verbatimLocality: Ewa, on stranded seaweed on beach; **Event:** verbatimEventDate: 14.iii.1946; **Record Level:** institutionCode: USNM
- **Type status:** Other material. **Occurrence:** catalogNumber: USNMENT 00972785; recordedBy: WW Wirth; individualCount: 1; sex: female; lifeStage: adult; **Location:** islandGroup: Hawaiian Islands; island: Oahu; country: United States; stateProvince: Hawaii; county: Honolulu; verbatimLocality: Ewa, on stranded seaweed on beach; **Event:** verbatimEventDate: 14.iii.1946; **Record Level:** institutionCode: USNM
- **Type status:** Other material. **Occurrence:** catalogNumber: USNMENT 00972786; recordedBy: WW Wirth; individualCount: 1; sex: male; lifeStage: adult; **Location:** islandGroup: Hawaiian Islands; island: Oahu; country: United States; stateProvince: Hawaii; county: Honolulu; verbatimLocality: Ewa, on stranded seaweed on beach; **Event:** verbatimEventDate: 14.iii.1946; **Record Level:** institutionCode: USNM
- **Type status:** Other material. **Occurrence:** catalogNumber: USNMENT 00972780; recordedBy: WW Wirth; individualCount: 1; sex: female; lifeStage: adult; **Location:** islandGroup: Hawaiian Islands; island: Oahu; country: United States; stateProvince: Hawaii; county: Honolulu; verbatimLocality: Ewa, on stranded seaweed on beach; **Event:** verbatimEventDate: 14.iii.1946; **Record Level:** institutionCode: USNM
- **Type status:** Other material. **Occurrence:** catalogNumber: USNMENT 00972787; recordedBy: WW Wirth; individualCount: 1; sex: female; lifeStage: adult; **Location:** islandGroup: Hawaiian Islands; island: Oahu; country: United States; stateProvince: Hawaii; county: Honolulu; verbatimLocality: Ewa, on stranded seaweed on beach; **Event:** verbatimEventDate: 14.iii.1946; **Record Level:** institutionCode: USNM
- **Type status:** Other material. **Occurrence:** catalogNumber: USNMENT 00972788; recordedBy: WW Wirth; individualCount: 1; sex: female; lifeStage: adult; **Location:** islandGroup: Hawaiian Islands; island: Oahu; country: United States; stateProvince: Hawaii; county: Honolulu; verbatimLocality: Ewa, on stranded seaweed on beach; **Event:** verbatimEventDate: 14.iii.1946; **Record Level:** institutionCode: USNM
- **Type status:** Other material. **Occurrence:** catalogNumber: USNMENT 00972789; recordedBy: WW Wirth; individualCount: 1; sex: female; lifeStage: adult; **Location:** islandGroup: Hawaiian Islands; island: Oahu; country: United States; stateProvince: Hawaii; county: Honolulu; verbatimLocality: Ewa, on stranded seaweed on beach; **Event:** verbatimEventDate: 14.iii.1946; **Record Level:** institutionCode: USNM
- **Type status:** Other material. **Occurrence:** catalogNumber: USNMENT 00972790; recordedBy: WW Wirth; individualCount: 1; sex: male; lifeStage: adult; **Location:** islandGroup: Hawaiian Islands; island: Oahu; country: United States; stateProvince: Hawaii; county: Honolulu; verbatimLocality: Ewa, on stranded seaweed on beach; **Event:** verbatimEventDate: 14.iii.1946; **Record Level:** institutionCode: USNM
- **Type status:** Other material. **Occurrence:** catalogNumber: USNMENT 00972791; recordedBy: WW Wirth; individualCount: 1; sex: female; lifeStage: adult; **Location:** islandGroup: Hawaiian Islands; island: Oahu; country: United States; stateProvince: Hawaii; county: Honolulu; verbatimLocality: Ewa, on stranded seaweed on beach; **Event:** verbatimEventDate: 14.iii.1946; **Record Level:** institutionCode: USNM
- **Type status:** Other material. **Occurrence:** catalogNumber: USNMENT 00972792; recordedBy: WW Wirth; individualCount: 1; sex: female; lifeStage: adult; **Location:** islandGroup: Hawaiian Islands; island: Oahu; country: United States; stateProvince: Hawaii; county: Honolulu; verbatimLocality: Ewa, on stranded seaweed on beach; **Event:** verbatimEventDate: 14.iii.1946; **Record Level:** institutionCode: USNM
- **Type status:** Other material. **Occurrence:** catalogNumber: USNMENT 00972793; recordedBy: WW Wirth; individualCount: 1; sex: female; lifeStage: adult; **Location:** islandGroup: Hawaiian Islands; island: Oahu; country: United States; stateProvince: Hawaii; county: Honolulu; verbatimLocality: Ewa, on stranded seaweed on beach; **Event:** verbatimEventDate: 14.iii.1946; **Record Level:** institutionCode: USNM
- **Type status:** Other material. **Occurrence:** catalogNumber: USNMENT 00972794; recordedBy: WW Wirth; individualCount: 1; sex: male; lifeStage: adult; **Location:** islandGroup: Hawaiian Islands; island: Oahu; country: United States; stateProvince: Hawaii; county: Honolulu; verbatimLocality: Ewa, on stranded seaweed on beach; **Event:** verbatimEventDate: 14.iii.1946; **Record Level:** institutionCode: USNM
- **Type status:** Other material. **Occurrence:** catalogNumber: USNMENT 00972795; recordedBy: WW Wirth; individualCount: 1; sex: male; lifeStage: adult; **Location:** islandGroup: Hawaiian Islands; island: Oahu; country: United States; stateProvince: Hawaii; county: Honolulu; verbatimLocality: Ewa, on stranded seaweed on beach; **Event:** verbatimEventDate: 14.iii.1946; **Record Level:** institutionCode: USNM
- **Type status:** Other material. **Occurrence:** recordedBy: CP Hoyt; individualCount: 1; lifeStage: adult; **Location:** islandGroup: Hawaiian Islands; island: Hawaii; country: United States; stateProvince: Hawaii; county: Hawaii; verbatimLocality: Keanakolu; verbatimElevation: 4500 ft.; **Event:** verbatimEventDate: 28.x.1952; **Record Level:** institutionCode: BPBM
- **Type status:** Other material. **Occurrence:** recordedBy: CP Hoyt; individualCount: 1; lifeStage: adult; **Location:** islandGroup: Hawaiian Islands; island: Oahu; country: United States; stateProvince: Hawaii; county: Honolulu; verbatimLocality: N. Halawa Trail; **Event:** verbatimEventDate: 24.xi.1952; **Record Level:** institutionCode: BPBM
- **Type status:** Other material. **Occurrence:** recordedBy: JL Gressitt; individualCount: 5; lifeStage: adult; **Location:** islandGroup: Hawaiian Islands; island: Oahu; country: United States; stateProvince: Hawaii; county: Honolulu; verbatimLocality: Kailua; **Event:** verbatimEventDate: 2.xii.1956; **Record Level:** institutionCode: BPBM
- **Type status:** Other material. **Occurrence:** recordedBy: RE Leech; individualCount: 1; lifeStage: adult; **Location:** islandGroup: Hawaiian Islands; island: Oahu; country: United States; stateProvince: Hawaii; county: Honolulu; verbatimLocality: Waianae Mts.; verbatimElevation: 2000 ft.; **Event:** verbatimEventDate: 11.x.1957; **Record Level:** institutionCode: BPBM
- **Type status:** Other material. **Occurrence:** recordedBy: no collector given; individualCount: 1; lifeStage: adult; **Location:** islandGroup: Hawaiian Islands; island: Oahu; country: United States; stateProvince: Hawaii; county: Honolulu; verbatimLocality: Barber's Point, at light trap; **Event:** verbatimEventDate: i.1959; **Record Level:** institutionCode: BPBM
- **Type status:** Other material. **Occurrence:** recordedBy: GD Butler; individualCount: 19; lifeStage: adult; **Location:** islandGroup: Hawaiian Islands; island: Laysan; country: United States; stateProvince: Hawaii; **Event:** verbatimEventDate: 27.iv-7.v.1959; **Record Level:** institutionCode: BPBM
- **Type status:** Other material. **Occurrence:** recordedBy: GD Butler; individualCount: 51; lifeStage: adult; **Location:** islandGroup: Hawaiian Islands; island: Laysan; country: United States; stateProvince: Hawaii; **Event:** verbatimEventDate: 20-28.vii.1959; **Record Level:** institutionCode: BPBM
- **Type status:** Other material. **Occurrence:** recordedBy: D Tsuda; individualCount: 1; lifeStage: adult; **Location:** islandGroup: Hawaiian Islands; island: Oahu; country: United States; stateProvince: Hawaii; county: Honolulu; verbatimLocality: Honolulu, Manoa; **Event:** verbatimEventDate: 2.iii.1962; **Record Level:** institutionCode: BPBM
- **Type status:** Other material. **Occurrence:** recordedBy: JW Beardsley; individualCount: 6; lifeStage: adult; **Location:** islandGroup: Hawaiian Islands; island: Laysan; country: United States; stateProvince: Hawaii; **Event:** verbatimEventDate: 16-21.vi.1962; **Record Level:** institutionCode: BPBM
- **Type status:** Other material. **Occurrence:** recordedBy: N Wilson; individualCount: 15; lifeStage: adult; **Location:** islandGroup: Hawaiian Islands; island: Laysan; country: United States; stateProvince: Hawaii; **Event:** verbatimEventDate: 8.xii.1963; **Record Level:** institutionCode: BPBM
- **Type status:** Other material. **Occurrence:** recordedBy: JW Beardsley; individualCount: 1179; lifeStage: adult; **Location:** islandGroup: Hawaiian Islands; island: Laysan; country: United States; stateProvince: Hawaii; verbatimLocality: sweeping around lagoon; **Event:** verbatimEventDate: 19.ix.1964; **Record Level:** institutionCode: BPBM
- **Type status:** Other material. **Occurrence:** recordedBy: JW Beardsley; individualCount: 25; lifeStage: adult; **Location:** islandGroup: Hawaiian Islands; island: Nihoa; country: United States; stateProvince: Hawaii; county: Nihoa; verbatimLocality: ex Chenopodium oahuense; **Event:** verbatimEventDate: 23.ix.1964; **Record Level:** institutionCode: BPBM
- **Type status:** Other material. **Occurrence:** recordedBy: JW Beardsley; individualCount: 12; lifeStage: adult; **Location:** islandGroup: Hawaiian Islands; island: Nihoa; country: United States; stateProvince: Hawaii; county: Nihoa; verbatimLocality: ex Eragrotis variabilis; **Event:** verbatimEventDate: 24.ix.1964; **Record Level:** institutionCode: BPBM
- **Type status:** Other material. **Occurrence:** recordedBy: JW Beardsley; individualCount: 1; lifeStage: adult; **Location:** islandGroup: Hawaiian Islands; island: Necker; country: United States; stateProvince: Hawaii; **Event:** verbatimEventDate: 26-27.ix.1964; **Record Level:** institutionCode: BPBM
- **Type status:** Other material. **Occurrence:** recordedBy: JL Gressitt; individualCount: 72; lifeStage: adult; **Location:** islandGroup: Hawaiian Islands; island: Molokai; country: United States; stateProvince: Hawaii; county: Maui; verbatimLocality: South Coast, Kaunakakai, E. of UH Fish Pond, shore mud; **Event:** verbatimEventDate: 23.iii.1970; **Record Level:** institutionCode: BPBM
- **Type status:** Other material. **Occurrence:** recordedBy: JL Gressitt; individualCount: 3; lifeStage: adult; **Location:** islandGroup: Hawaiian Islands; island: Molokai; country: United States; stateProvince: Hawaii; county: Maui; verbatimLocality: Alii Pond; **Event:** verbatimEventDate: 24.iii.1970; **Record Level:** institutionCode: BPBM
- **Type status:** Other material. **Occurrence:** recordedBy: J Strazanac; individualCount: 6; lifeStage: adult; **Location:** islandGroup: Hawaiian Islands; island: Nihoa; country: United States; stateProvince: Hawaii; county: Nihoa; verbatimLocality: Miller Valley; verbatimElevation: 100 ft.; **Event:** verbatimEventDate: 22.vi.1990; **Record Level:** institutionCode: BPBM
- **Type status:** Other material. **Occurrence:** recordedBy: DJ Preston; individualCount: 7; lifeStage: adult; **Location:** islandGroup: Hawaiian Islands; island: Oahu; country: United States; stateProvince: Hawaii; county: Honolulu; verbatimLocality: Pearl Harbor, mouth of Waimalu Stream; **Identification:** identifiedBy: DJ Preston; **Event:** verbatimEventDate: 3.xi.1997; **Record Level:** institutionCode: BPBM
- **Type status:** Other material. **Occurrence:** recordedBy: DJ Preston, R Englund, R Wolfe; individualCount: 3; lifeStage: adult; **Location:** islandGroup: Hawaiian Islands; island: Oahu; country: United States; stateProvince: Hawaii; county: Honolulu; verbatimLocality: Pearl Harbor, Honoluiuli Stream, near pipeline bridge; **Identification:** identifiedBy: DJ Preston; **Event:** verbatimEventDate: 19.xi.1997; **Record Level:** institutionCode: BPBM
- **Type status:** Other material. **Occurrence:** recordedBy: DJ Preston, R Englund, R Wolfe; individualCount: 1; lifeStage: adult; **Location:** islandGroup: Hawaiian Islands; island: Oahu; country: United States; stateProvince: Hawaii; county: Honolulu; verbatimLocality: Pearl Harbor, Halawa Stream, mud bar near Salt Lake Blvd Bridge; **Identification:** identifiedBy: DJ Preston; **Event:** verbatimEventDate: 8.xii.1997; **Record Level:** institutionCode: BPBM
- **Type status:** Other material. **Occurrence:** recordedBy: DJ Preston; individualCount: 1; lifeStage: adult; **Location:** islandGroup: Hawaiian Islands; island: Oahu; country: United States; stateProvince: Hawaii; county: Honolulu; verbatimLocality: Waianae Mountains, Mt Kaala, at summit area near newly buried electrical cable; **Identification:** identifiedBy: DJ Preston; **Event:** verbatimEventDate: ii.1998; **Record Level:** institutionCode: BPBM
- **Type status:** Other material. **Occurrence:** recordedBy: DJ Preston, R Englund; individualCount: 5; lifeStage: adult; **Location:** islandGroup: Hawaiian Islands; island: Oahu; country: United States; stateProvince: Hawaii; county: Honolulu; verbatimLocality: Waianae Mountains, Pearl Harbor, Makaha Wetlands, N of roadway; **Identification:** identifiedBy: DJ Preston; **Event:** verbatimEventDate: 2.ii.1998; **Record Level:** institutionCode: BPBM
- **Type status:** Other material. **Occurrence:** recordedBy: DJ Preston, R Englund, R Wolfe; individualCount: 5; lifeStage: adult; **Location:** islandGroup: Hawaiian Islands; island: Oahu; country: United States; stateProvince: Hawaii; county: Honolulu; verbatimLocality: Koolau Mountains, Pearl Harbor, Halawa Stream, S of Salt Lake Blvd Bridge; **Identification:** identifiedBy: DJ Preston; **Event:** verbatimEventDate: 23.iii.1998; **Record Level:** institutionCode: BPBM
- **Type status:** Other material. **Occurrence:** recordedBy: WC Gagne; individualCount: 10; lifeStage: adult; **Location:** islandGroup: Hawaiian Islands; island: Nihoa; country: United States; stateProvince: Hawaii; county: Nihoa; verbatimLocality: Miller Valley; verbatimElevation: 150 ft.; **Event:** verbatimEventDate: 29.iv.1983; **Record Level:** institutionCode: BPBM
- **Type status:** Other material. **Occurrence:** recordedBy: K Arakaki; individualCount: 12; lifeStage: adult; **Location:** islandGroup: Hawaiian Islands; island: Oahu; country: United States; stateProvince: Hawaii; county: Honolulu; verbatimLocality: Baisdell Park, shoreline; **Identification:** identifiedBy: K Arakaki; **Event:** verbatimEventDate: 28.v.1998; **Record Level:** institutionCode: BPBM
- **Type status:** Other material. **Occurrence:** recordedBy: GA Samuelson; individualCount: 8; lifeStage: adult; **Location:** islandGroup: Hawaiian Islands; island: Oahu; country: United States; stateProvince: Hawaii; county: Honolulu; verbatimLocality: Waimalu Stream, near Blaisdell Park on narrow trail; **Identification:** identifiedBy: K Arakaki; **Event:** verbatimEventDate: 28.v.1998; **Record Level:** institutionCode: BPBM
- **Type status:** Other material. **Occurrence:** recordedBy: GA Samuelson; individualCount: 2; lifeStage: adult; **Location:** islandGroup: Hawaiian Islands; island: Oahu; country: United States; stateProvince: Hawaii; county: Honolulu; verbatimLocality: Baisdell Park, Waimalu Stream; **Identification:** identifiedBy: K Arakaki; **Event:** verbatimEventDate: 28.v.1998; **Record Level:** institutionCode: BPBM
- **Type status:** Other material. **Occurrence:** recordedBy: GA Samuelson; individualCount: 1; lifeStage: adult; **Location:** islandGroup: Hawaiian Islands; island: Oahu; country: United States; stateProvince: Hawaii; county: Honolulu; verbatimLocality: Halawa Stream (lower); **Identification:** identifiedBy: K Arakaki; **Event:** verbatimEventDate: 2.vi.1998; **Record Level:** institutionCode: BPBM
- **Type status:** Other material. **Occurrence:** recordedBy: K Arakaki; individualCount: 2; lifeStage: adult; **Location:** islandGroup: Hawaiian Islands; island: Oahu; country: United States; stateProvince: Hawaii; county: Honolulu; verbatimLocality: Pearl Harbor, Waiawa Springs; **Identification:** identifiedBy: K Arakaki; **Event:** verbatimEventDate: 3.vi.1998; **Record Level:** institutionCode: BPBM
- **Type status:** Other material. **Occurrence:** recordedBy: K Arakaki; individualCount: 7; lifeStage: adult; **Location:** islandGroup: Hawaiian Islands; island: Oahu; country: United States; stateProvince: Hawaii; county: Honolulu; verbatimLocality: Honouliuli; **Identification:** identifiedBy: K Arakaki; **Event:** verbatimEventDate: 17.vi.1998; **Record Level:** institutionCode: BPBM
- **Type status:** Other material. **Occurrence:** recordedBy: K Arakaki; individualCount: 6; lifeStage: adult; **Location:** islandGroup: Hawaiian Islands; island: Oahu; country: United States; stateProvince: Hawaii; county: Honolulu; verbatimLocality: Pearl Harbor, Puohala Marsh; **Identification:** identifiedBy: K Arakaki; **Event:** verbatimEventDate: 19.vi.1998; **Record Level:** institutionCode: BPBM
- **Type status:** Other material. **Occurrence:** recordedBy: K Arakaki; individualCount: 1; lifeStage: adult; **Location:** islandGroup: Hawaiian Islands; island: Oahu; country: United States; stateProvince: Hawaii; county: Honolulu; verbatimLocality: Pearl Harbor, E'o Stream, waterline near bridge foundation; **Identification:** identifiedBy: K Arakaki; **Event:** verbatimEventDate: 19.vi.1998; **Record Level:** institutionCode: BPBM
- **Type status:** Other material. **Occurrence:** recordedBy: K Arakaki; individualCount: 1; lifeStage: adult; **Location:** islandGroup: Hawaiian Islands; island: Oahu; country: United States; stateProvince: Hawaii; county: Honolulu; verbatimLocality: Aiea Stream; **Identification:** identifiedBy: K Arakaki; **Event:** verbatimEventDate: 24.vi.1998; **Record Level:** institutionCode: BPBM
- **Type status:** Other material. **Occurrence:** recordedBy: K Arakaki; individualCount: 1; lifeStage: adult; **Location:** islandGroup: Hawaiian Islands; island: Oahu; country: United States; stateProvince: Hawaii; county: Honolulu; verbatimLocality: Aiea Bay; **Identification:** identifiedBy: K Arakaki; **Event:** verbatimEventDate: 24.vi.1998; **Record Level:** institutionCode: BPBM
- **Type status:** Other material. **Occurrence:** recordedBy: K Arakaki; individualCount: 1; lifeStage: adult; **Location:** islandGroup: Hawaiian Islands; island: Oahu; country: United States; stateProvince: Hawaii; county: Honolulu; verbatimLocality: Kalauao stream; **Identification:** identifiedBy: K Arakaki; **Event:** verbatimEventDate: 29.vi.1998; **Record Level:** institutionCode: BPBM
- **Type status:** Other material. **Occurrence:** recordedBy: K Arakaki; individualCount: 1; lifeStage: adult; **Location:** islandGroup: Hawaiian Islands; island: Oahu; country: United States; stateProvince: Hawaii; county: Honolulu; verbatimLocality: Pearl Harbor, Waiau Hawaiian Electric Power Plant, near dam at lower end of pond; **Identification:** identifiedBy: K Arakaki; **Event:** verbatimEventDate: 30.vi.1998; **Record Level:** institutionCode: BPBM

##### Ecological interactions

###### Native status

Immigrant?

##### Distribution

Pacific: Wake Island. Hawaiian Islands, Laysan, Necker, Nihoa, Kauai, Oahu, Molokai, Maui, Hawaii.

##### Notes

47 specimens from USNM not examined.

#### Scatella stagnalis

(Fallen, 1813)

##### Materials

- **Type status:** Holotype. **Occurrence:** recordedBy: no collector given; lifeStage: adult; **Location:** islandGroup: Hawaiian Islands; country: United States; stateProvince: Hawaii; **Record Level:** institutionCode: MZLU
- **Type status:** Other material. **Occurrence:** recordedBy: JA Tenorio; individualCount: 9; lifeStage: adult; **Location:** islandGroup: Hawaiian Islands; island: Hawaii; country: United States; stateProvince: Hawaii; county: Hawaii; verbatimLocality: Hilo, Waiakea Pond; **Identification:** identifiedBy: JA Tenorio; **Event:** verbatimEventDate: 27.xii.1969; **Record Level:** institutionCode: BPBM
- **Type status:** Other material. **Occurrence:** recordedBy: Tenorio; individualCount: 1; lifeStage: adult; **Location:** islandGroup: Hawaiian Islands; island: Hawaii; country: United States; stateProvince: Hawaii; county: Hawaii; verbatimLocality: Kalapana, on beach; **Identification:** identifiedBy: JA Tenorio; **Event:** verbatimEventDate: 29.xi.1969; **Record Level:** institutionCode: BPBM
- **Type status:** Other material. **Occurrence:** recordedBy: CW Mills III; individualCount: 2; lifeStage: adult; **Location:** islandGroup: Hawaiian Islands; island: Oahu; country: United States; stateProvince: Hawaii; county: Honolulu; verbatimLocality: Hickam Air Force Base; **Identification:** identifiedBy: K Arakaki; **Event:** verbatimEventDate: 29.x.1977; **Record Level:** institutionCode: BPBM
- **Type status:** Other material. **Occurrence:** recordedBy: CW Mill III; individualCount: 5; lifeStage: adult; **Location:** islandGroup: Hawaiian Islands; island: Oahu; country: United States; stateProvince: Hawaii; county: Honolulu; verbatimLocality: Hickam Air Force Base, at light; **Identification:** identifiedBy: K Arakaki; **Event:** verbatimEventDate: 8.i.1978; **Record Level:** institutionCode: BPBM
- **Type status:** Other material. **Occurrence:** recordedBy: WC Gagne; individualCount: 3; lifeStage: adult; **Location:** islandGroup: Hawaiian Islands; island: Oahu; country: United States; stateProvince: Hawaii; county: Honolulu; verbatimLocality: Barber's Point; **Identification:** identifiedBy: K Arakaki; **Event:** verbatimEventDate: 6.ii.1982; **Record Level:** institutionCode: BPBM
- **Type status:** Other material. **Occurrence:** recordedBy: GM Nishida; individualCount: 20; lifeStage: adult; **Location:** islandGroup: Hawaiian Islands; island: Midway; country: United States; stateProvince: Hawaii; verbatimLocality: Sand Island, Dump Lake; **Identification:** identifiedBy: K Arakaki; **Event:** verbatimEventDate: 17.xii.1997; **Record Level:** institutionCode: BPBM
- **Type status:** Other material. **Occurrence:** recordedBy: DJ Preston; individualCount: 3; lifeStage: adult; **Location:** islandGroup: Hawaiian Islands; island: Oahu; country: United States; stateProvince: Hawaii; county: Honolulu; verbatimLocality: Mt Kaala, at summit area near newly buried electrical cable; **Identification:** identifiedBy: K Arakaki; **Event:** verbatimEventDate: ii.1998; **Record Level:** institutionCode: BPBM
- **Type status:** Other material. **Occurrence:** recordedBy: DJ Preston; individualCount: 3; lifeStage: adult; **Location:** islandGroup: Hawaiian Islands; island: Oahu; country: United States; stateProvince: Hawaii; county: Honolulu; verbatimLocality: Koolau Mountains, Pearl Harbor, Halawa Stream, S. of Salt Lake Bird Bridge; **Identification:** identifiedBy: K Arakaki; **Event:** verbatimEventDate: 23.iii.1998; **Record Level:** institutionCode: BPBM
- **Type status:** Other material. **Occurrence:** recordedBy: GA Samuelson; individualCount: 1; lifeStage: adult; **Location:** islandGroup: Hawaiian Islands; island: Oahu; country: United States; stateProvince: Hawaii; county: Honolulu; verbatimLocality: Pearl Harbor, Middle Loch near Waiwa Springs, along shore; **Identification:** identifiedBy: K Arakaki; **Event:** verbatimEventDate: 6.v.1998; **Record Level:** institutionCode: BPBM
- **Type status:** Other material. **Occurrence:** recordedBy: K Arakaki; individualCount: 1; lifeStage: adult; **Location:** islandGroup: Hawaiian Islands; island: Oahu; country: United States; stateProvince: Hawaii; county: Honolulu; verbatimLocality: Baisdell Park, Waimalu Stream; **Identification:** identifiedBy: K Arakaki; **Event:** verbatimEventDate: 28.v.1998; **Record Level:** institutionCode: BPBM
- **Type status:** Other material. **Occurrence:** recordedBy: GA Samuelson; individualCount: 1; lifeStage: adult; **Location:** islandGroup: Hawaiian Islands; island: Oahu; country: United States; stateProvince: Hawaii; county: Honolulu; verbatimLocality: Koolau Mountains, Halawa Stream, McDonald's Area; **Identification:** identifiedBy: K Arakaki; **Event:** verbatimEventDate: 2.vi.1998; **Record Level:** institutionCode: BPBM
- **Type status:** Other material. **Occurrence:** recordedBy: GA Samuelson; individualCount: 1; lifeStage: adult; **Location:** islandGroup: Hawaiian Islands; island: Oahu; country: United States; stateProvince: Hawaii; county: Honolulu; verbatimLocality: Waikele Stream, near Cultural Garden; **Identification:** identifiedBy: K Arakaki; **Event:** verbatimEventDate: 9.vi.1998; **Record Level:** institutionCode: BPBM
- **Type status:** Other material. **Occurrence:** recordedBy: K Arakaki; individualCount: 3; lifeStage: adult; **Location:** islandGroup: Hawaiian Islands; island: Oahu; country: United States; stateProvince: Hawaii; county: Honolulu; verbatimLocality: Waiawa Stream; **Identification:** identifiedBy: K Arakaki; **Event:** verbatimEventDate: 16.vi.1998; **Record Level:** institutionCode: BPBM
- **Type status:** Other material. **Occurrence:** recordedBy: K Arakaki; individualCount: 1; lifeStage: adult; **Location:** islandGroup: Hawaiian Islands; island: Oahu; country: United States; stateProvince: Hawaii; county: Honolulu; verbatimLocality: Pearl Harbor, Honouliuli; **Identification:** identifiedBy: K Arakaki; **Event:** verbatimEventDate: 17.vi.1998; **Record Level:** institutionCode: BPBM
- **Type status:** Other material. **Occurrence:** recordedBy: K Arakaki; individualCount: 3; lifeStage: adult; **Location:** islandGroup: Hawaiian Islands; island: Oahu; country: United States; stateProvince: Hawaii; county: Honolulu; verbatimLocality: Pearl Harbor, Puohala Marsh, first pool; **Identification:** identifiedBy: K Arakaki; **Event:** verbatimEventDate: 19.vi.1998; **Record Level:** institutionCode: BPBM
- **Type status:** Other material. **Occurrence:** recordedBy: K Arakaki; individualCount: 2; lifeStage: adult; **Location:** islandGroup: Hawaiian Islands; island: Oahu; country: United States; stateProvince: Hawaii; county: Honolulu; verbatimLocality: Kapakahi Stream, at edge; **Identification:** identifiedBy: K Arakaki; **Event:** verbatimEventDate: 19.vi.1998; **Record Level:** institutionCode: BPBM
- **Type status:** Other material. **Occurrence:** recordedBy: K Arakaki; individualCount: 9; lifeStage: adult; **Location:** islandGroup: Hawaiian Islands; island: Oahu; country: United States; stateProvince: Hawaii; county: Honolulu; verbatimLocality: Pearly Harbor, Waiau Hawaiian Electric Power Plant, near dam at lower end of pond; **Identification:** identifiedBy: K Arakaki; **Event:** verbatimEventDate: 20.vi.1998; **Record Level:** institutionCode: BPBM
- **Type status:** Other material. **Occurrence:** recordedBy: K Arakaki; individualCount: 8; lifeStage: adult; **Location:** islandGroup: Hawaiian Islands; island: Oahu; country: United States; stateProvince: Hawaii; county: Honolulu; verbatimLocality: Pearl Harbor, Aiea Bay, park area; **Identification:** identifiedBy: K Arakaki; **Event:** verbatimEventDate: 24.vi.1998; **Record Level:** institutionCode: BPBM
- **Type status:** Other material. **Occurrence:** recordedBy: K Arakaki; individualCount: 1; lifeStage: adult; **Location:** islandGroup: Hawaiian Islands; island: Oahu; country: United States; stateProvince: Hawaii; county: Honolulu; verbatimLocality: Aiea Bay, Aiea Stream, mouth; **Identification:** identifiedBy: K Arakaki; **Event:** verbatimEventDate: 24.vi.1998; **Record Level:** institutionCode: BPBM
- **Type status:** Other material. **Occurrence:** recordedBy: K Arakaki; individualCount: 2; lifeStage: adult; **Location:** islandGroup: Hawaiian Islands; island: Oahu; country: United States; stateProvince: Hawaii; county: Honolulu; verbatimLocality: Aiea Stream; **Identification:** identifiedBy: K Arakaki; **Event:** verbatimEventDate: 24.vi.1998; **Record Level:** institutionCode: BPBM
- **Type status:** Other material. **Occurrence:** recordedBy: K Arakaki; individualCount: 2; lifeStage: adult; **Location:** islandGroup: Hawaiian Islands; island: Oahu; country: United States; stateProvince: Hawaii; county: Honolulu; verbatimLocality: Kalauao Stream; **Identification:** identifiedBy: K Arakaki; **Event:** verbatimEventDate: 29.vi.1998; **Record Level:** institutionCode: BPBM
- **Type status:** Other material. **Occurrence:** recordedBy: K Arakaki; individualCount: 3; lifeStage: adult; **Location:** islandGroup: Hawaiian Islands; island: Oahu; country: United States; stateProvince: Hawaii; county: Honolulu; verbatimLocality: Pearl Harbor, Kalauao ponds, stream from watercress ponds; **Identification:** identifiedBy: K Arakaki; **Event:** verbatimEventDate: 29.vi.1998; **Record Level:** institutionCode: BPBM
- **Type status:** Other material. **Occurrence:** recordedBy: K Arakaki; individualCount: 4; lifeStage: adult; **Location:** islandGroup: Hawaiian Islands; island: Oahu; country: United States; stateProvince: Hawaii; county: Honolulu; verbatimLocality: Pearl Harbor, E'o, Canal Ted Makalena Golf Course, on fairway; **Identification:** identifiedBy: K Arakaki; **Event:** verbatimEventDate: 27.vii.1998; **Record Level:** institutionCode: BPBM
- **Type status:** Other material. **Occurrence:** recordedBy: RA Englund; individualCount: 6; lifeStage: adult; **Location:** islandGroup: Hawaiian Islands; island: Kauai; country: United States; stateProvince: Hawaii; county: Kauai; verbatimLocality: Mouth of Lower Kaeke Stream; **Identification:** identifiedBy: K Arakaki; **Event:** verbatimEventDate: 28.ix.2001; **Record Level:** institutionCode: BPBM
- **Type status:** Other material. **Occurrence:** recordedBy: D. Montgomery-Brock; individualCount: 5; lifeStage: adult; **Location:** islandGroup: Hawaiian Islands; island: Oahu; country: United States; stateProvince: Hawaii; county: Honolulu; verbatimLocality: Kahuku, Kahuku Shrimp Farm; **Identification:** identifiedBy: K Arakaki; **Event:** verbatimEventDate: 2.iv.2004; **Record Level:** institutionCode: BPBM

##### Ecological interactions

###### Native status

Immigrant.

##### Distribution

Cosmopolitan. Hawaiian Islands: Midway, Hawaii, Oahu, Hawaii.

#### Scatella terryi

Cresson, 1926

##### Materials

- **Type status:** Holotype. **Occurrence:** catalogNumber: 9175; recordedBy: EH Bryan Jr.; individualCount: 1; sex: male; lifeStage: adult; **Location:** islandGroup: Hawaiian Islands; island: Oahu; country: United States; stateProvince: Hawaii; county: Honolulu; verbatimLocality: Wawamalu Beach, near Koko Crater; **Event:** verbatimEventDate: 12.xii.1922; **Record Level:** institutionCode: BPBM
- **Type status:** Other material. **Occurrence:** recordedBy: WW Wirth; individualCount: 1; sex: male; lifeStage: adult; **Location:** islandGroup: Hawaiian Islands; island: Oahu; country: United States; stateProvince: Hawaii; county: Honolulu; verbatimLocality: Hanauma Bay, on rocks by sea; **Event:** verbatimEventDate: 4.i.1946; **Record Level:** institutionCode: BPBM
- **Type status:** Other material. **Occurrence:** recordedBy: WW Wirth; individualCount: 2; lifeStage: adult; **Location:** islandGroup: Hawaiian Islands; island: Oahu; country: United States; stateProvince: Hawaii; county: Honolulu; verbatimLocality: Waimea, on rocks by sea; **Event:** verbatimEventDate: 15.i.1946; **Record Level:** institutionCode: BPBM
- **Type status:** Other material. **Occurrence:** catalogNumber: USNMENT 00972796; recordedBy: WW Wirth; individualCount: 1; sex: male; lifeStage: adult; **Location:** islandGroup: Hawaiian Islands; island: Oahu; country: United States; stateProvince: Hawaii; county: Honolulu; verbatimLocality: Waimea, on rocks by sea; **Event:** verbatimEventDate: 15.i.1946; **Record Level:** institutionCode: USNM
- **Type status:** Other material. **Occurrence:** catalogNumber: USNMENT 00972797; recordedBy: WW Wirth; individualCount: 1; sex: male; lifeStage: adult; **Location:** islandGroup: Hawaiian Islands; island: Oahu; country: United States; stateProvince: Hawaii; county: Honolulu; verbatimLocality: Waimea, on rocks by sea; **Event:** verbatimEventDate: 15.i.1946; **Record Level:** institutionCode: USNM
- **Type status:** Other material. **Occurrence:** recordedBy: MS Adachi; individualCount: 2; lifeStage: adult; **Location:** islandGroup: Hawaiian Islands; island: Oahu; country: United States; stateProvince: Hawaii; county: Honolulu; verbatimLocality: Waimanalo; **Identification:** identifiedBy: JA Tenorio; **Event:** verbatimEventDate: 26.xii.1949; **Record Level:** institutionCode: BPBM

##### Ecological interactions

###### Native status

Endemic.

##### Distribution

Hawaiian Islands: Oahu, Maui.

#### Scatella warreni

Cresson, 1926

##### Materials

- **Type status:** Holotype. **Occurrence:** catalogNumber: 353; recordedBy: EH Bryan Jr.; individualCount: 1; sex: male; lifeStage: adult; **Location:** islandGroup: Hawaiian Islands; island: Maui; country: United States; stateProvince: Hawaii; county: Maui; verbatimLocality: Haipuaena; **Event:** verbatimEventDate: 25.vii.1920; **Record Level:** institutionCode: BPBM
- **Type status:** Paratype. **Occurrence:** catalogNumber: 353a; recordedBy: EH Bryan Jr.; individualCount: 1; sex: female; lifeStage: adult; **Location:** islandGroup: Hawaiian Islands; island: Maui; country: United States; stateProvince: Hawaii; county: Maui; verbatimLocality: Haipuaena; **Event:** verbatimEventDate: 25.vii.1920; **Record Level:** institutionCode: BPBM
- **Type status:** Paratype. **Occurrence:** recordedBy: EH Bryan Jr.; individualCount: 3; lifeStage: adult; **Location:** islandGroup: Hawaiian Islands; island: Maui; country: United States; stateProvince: Hawaii; county: Maui; verbatimLocality: Haipuaena; **Event:** verbatimEventDate: 25.vii.1920; **Record Level:** institutionCode: UHM
- **Type status:** Paratype. **Occurrence:** recordedBy: EH Bryan Jr.; individualCount: 3; lifeStage: adult; **Location:** islandGroup: Hawaiian Islands; island: Maui; country: United States; stateProvince: Hawaii; county: Maui; verbatimLocality: Haipuaena; **Identification:** identifiedBy: WW Wirth; **Event:** verbatimEventDate: 25.vi.1920; **Record Level:** institutionCode: BPBM
- **Type status:** Other material. **Occurrence:** recordedBy: NLH Krauss; individualCount: 9; lifeStage: adult; **Location:** islandGroup: Hawaiian Islands; island: Maui; country: United States; stateProvince: Hawaii; county: Maui; verbatimLocality: Nahiku; **Identification:** identifiedBy: JA Tenorio; **Event:** verbatimEventDate: date unclear; **Record Level:** institutionCode: BPBM
- **Type status:** Other material. **Occurrence:** recordedBy: no collector given; individualCount: 1; lifeStage: adult; **Location:** islandGroup: Hawaiian Islands; island: Oahu; country: United States; stateProvince: Hawaii; county: Honolulu; **Identification:** identifiedBy: ET Cresson; **Event:** verbatimEventDate: i.1913; **Record Level:** institutionCode: BPBM
- **Type status:** Other material. **Occurrence:** recordedBy: A Warren; individualCount: 2; lifeStage: adult; **Location:** islandGroup: Hawaiian Islands; island: Oahu; country: United States; stateProvince: Hawaii; county: Honolulu; verbatimLocality: Honolulu; **Identification:** identifiedBy: ET Cresson; **Event:** verbatimEventDate: 26.x.1913; **Record Level:** institutionCode: BPBM
- **Type status:** Other material. **Occurrence:** recordedBy: JF Illingworth; individualCount: 4; lifeStage: adult; **Location:** islandGroup: Hawaiian Islands; island: Oahu; country: United States; stateProvince: Hawaii; county: Honolulu; **Event:** verbatimEventDate: vii.1914; **Record Level:** institutionCode: BPBM
- **Type status:** Other material. **Occurrence:** recordedBy: JF Illingworth; individualCount: 1; lifeStage: adult; **Location:** islandGroup: Hawaiian Islands; island: Maui; country: United States; stateProvince: Hawaii; county: Maui; verbatimLocality: Iao Valley; **Identification:** identifiedBy: ET Cresson; **Event:** verbatimEventDate: 3.i.1915; **Record Level:** institutionCode: BPBM
- **Type status:** Other material. **Occurrence:** recordedBy: C Bridwell; individualCount: 1; lifeStage: adult; **Location:** islandGroup: Hawaiian Islands; island: Oahu; country: United States; stateProvince: Hawaii; county: Honolulu; verbatimLocality: Lanihuli R.; **Identification:** identifiedBy: WW Wirth; **Event:** verbatimEventDate: 2.ix.1916; **Record Level:** institutionCode: BPBM
- **Type status:** Other material. **Occurrence:** recordedBy: EH Bryan Jr.; individualCount: 9; lifeStage: adult; **Location:** islandGroup: Hawaiian Islands; island: Oahu; country: United States; stateProvince: Hawaii; county: Honolulu; verbatimLocality: Moanalua; **Identification:** identifiedBy: WW Wirth; **Event:** verbatimEventDate: 9.iv.1922; **Record Level:** institutionCode: BPBM
- **Type status:** Other material. **Occurrence:** recordedBy: EH Bryan Jr.; individualCount: 1; lifeStage: adult; **Location:** islandGroup: Hawaiian Islands; island: Oahu; country: United States; stateProvince: Hawaii; county: Honolulu; verbatimLocality: Kalihi Valley; **Identification:** identifiedBy: ET Cresson; **Event:** verbatimEventDate: 12.ii.1923; **Record Level:** institutionCode: BPBM
- **Type status:** Other material. **Occurrence:** recordedBy: EH Bryan Jr.; individualCount: 1; lifeStage: adult; **Location:** islandGroup: Hawaiian Islands; island: Oahu; country: United States; stateProvince: Hawaii; county: Honolulu; verbatimLocality: Palolo; **Identification:** identifiedBy: ET Cresson; **Event:** verbatimEventDate: 25.ii.1925; **Record Level:** institutionCode: BPBM
- **Type status:** Other material. **Occurrence:** recordedBy: EH Bryan Jr.; individualCount: 2; lifeStage: adult; **Location:** islandGroup: Hawaiian Islands; island: Oahu; country: United States; stateProvince: Hawaii; county: Honolulu; verbatimLocality: Kamokuiki Valley, about water pool; **Event:** verbatimEventDate: 13.iv.1933; **Record Level:** institutionCode: BPBM
- **Type status:** Other material. **Occurrence:** recordedBy: EH Bryan Jr.; individualCount: 1; lifeStage: adult; **Location:** islandGroup: Hawaiian Islands; island: Oahu; country: United States; stateProvince: Hawaii; county: Honolulu; verbatimLocality: Kamokuiki Valley, ex stagnant water; **Event:** verbatimEventDate: 13.iv.1933; **Record Level:** institutionCode: BPBM
- **Type status:** Other material. **Occurrence:** recordedBy: K Ito; individualCount: 9; lifeStage: adult; **Location:** islandGroup: Hawaiian Islands; island: Oahu; country: United States; stateProvince: Hawaii; county: Honolulu; verbatimLocality: Waipio, Waihole Ditch; **Identification:** identifiedBy: JA Tenorio; **Event:** verbatimEventDate: 26.iv.1935; **Record Level:** institutionCode: BPBM
- **Type status:** Other material. **Occurrence:** recordedBy: RL Usinger; individualCount: 1; lifeStage: adult; **Location:** islandGroup: Hawaiian Islands; island: Oahu; country: United States; stateProvince: Hawaii; county: Honolulu; verbatimLocality: Waianae; **Event:** verbatimEventDate: 7.vii.1935; **Record Level:** institutionCode: BPBM
- **Type status:** Other material. **Occurrence:** recordedBy: RL Usinger; individualCount: 3; lifeStage: adult; **Location:** islandGroup: Hawaiian Islands; island: Hawaii; country: United States; stateProvince: Hawaii; county: Hawaii; verbatimLocality: Hamakua Ditch Trail; **Identification:** identifiedBy: WW Wirth; **Event:** verbatimEventDate: 15.viii.1935; **Record Level:** institutionCode: BPBM
- **Type status:** Other material. **Occurrence:** recordedBy: FX Williams; individualCount: 1; lifeStage: adult; **Location:** islandGroup: Hawaiian Islands; island: Oahu; country: United States; stateProvince: Hawaii; county: Honolulu; verbatimLocality: Waianae, swift water ditch; **Event:** verbatimEventDate: 10.iii.1936; **Record Level:** institutionCode: BPBM
- **Type status:** Other material. **Occurrence:** recordedBy: EC Zimmerman; individualCount: 13; lifeStage: adult; **Location:** islandGroup: Hawaiian Islands; island: Oahu; country: United States; stateProvince: Hawaii; county: Honolulu; verbatimLocality: Kalihi Valley; **Event:** verbatimEventDate: 13.iii.1937; **Record Level:** institutionCode: BPBM
- **Type status:** Other material. **Occurrence:** recordedBy: FX Williams; individualCount: 2; lifeStage: adult; **Location:** islandGroup: Hawaiian Islands; island: Oahu; country: United States; stateProvince: Hawaii; county: Honolulu; verbatimLocality: Honolulu, Kalihi Stream, wet boulder; **Event:** verbatimEventDate: 14.iii.1937; **Record Level:** institutionCode: BPBM
- **Type status:** Other material. **Occurrence:** recordedBy: RL Usinger; individualCount: 1; lifeStage: adult; **Location:** islandGroup: Hawaiian Islands; island: Kauai; country: United States; stateProvince: Hawaii; county: Kauai; verbatimLocality: Kumuwela; **Identification:** identifiedBy: WW Wirth; **Event:** verbatimEventDate: 20.xii.1935; **Record Level:** institutionCode: BPBM
- **Type status:** Other material. **Occurrence:** recordedBy: EH Bryan Jr.; individualCount: 1; lifeStage: adult; **Location:** islandGroup: Hawaiian Islands; island: Maui; country: United States; stateProvince: Hawaii; county: Maui; verbatimLocality: Wailuaiki; **Identification:** identifiedBy: WW Wirth; **Event:** verbatimEventDate: 2.vii.1920; **Record Level:** institutionCode: BPBM
- **Type status:** Other material. **Occurrence:** recordedBy: O Bryant; individualCount: 1; lifeStage: adult; **Location:** islandGroup: Hawaiian Islands; island: Maui; country: United States; stateProvince: Hawaii; county: Maui; verbatimLocality: Olinda; verbatimElevation: 4500 ft.; **Identification:** identifiedBy: WW Wirth; **Event:** verbatimEventDate: 8.iv.1932; **Record Level:** institutionCode: BPBM
- **Type status:** Other material. **Occurrence:** recordedBy: EY Hosaka; individualCount: 1; lifeStage: adult; **Location:** islandGroup: Hawaiian Islands; island: Hawaii; country: United States; stateProvince: Hawaii; county: Hawaii; verbatimLocality: Kohala, near Kahua Ranch; verbatimElevation: 4500 ft.; **Identification:** identifiedBy: WW Wirth; **Event:** verbatimEventDate: 4.ix.1936; **Record Level:** institutionCode: BPBM
- **Type status:** Other material. **Occurrence:** recordedBy: WC Gagne; individualCount: 1; lifeStage: adult; **Location:** islandGroup: Hawaiian Islands; island: Maui; country: United States; stateProvince: Hawaii; county: Maui; verbatimLocality: Haleakala National Park, Kipahulu Valley; verbatimElevation: 1500 ft.; **Identification:** identifiedBy: NL Evenhuis; **Event:** verbatimEventDate: 5.vii.1980; **Record Level:** institutionCode: BPBM
- **Type status:** Other material. **Occurrence:** recordedBy: EH Bryan Jr.; individualCount: 3; lifeStage: adult; **Location:** islandGroup: Hawaiian Islands; island: Maui; country: United States; stateProvince: Hawaii; county: Maui; verbatimLocality: Haipuaena; **Identification:** identifiedBy: ET Cresson; **Event:** verbatimEventDate: 25.vi.1920; **Record Level:** institutionCode: BPBM
- **Type status:** Other material. **Occurrence:** recordedBy: FX Williams; individualCount: 2; lifeStage: adult; **Location:** islandGroup: Hawaiian Islands; island: Hawaii; country: United States; stateProvince: Hawaii; county: Hawaii; verbatimLocality: Akaka Falls, about water; **Identification:** identifiedBy: WW Wirth; **Event:** verbatimEventDate: 24.x.1931; **Record Level:** institutionCode: BPBM
- **Type status:** Other material. **Occurrence:** recordedBy: FX Williams; individualCount: 1; lifeStage: adult; **Location:** islandGroup: Hawaiian Islands; island: Oahu; country: United States; stateProvince: Hawaii; county: Honolulu; verbatimLocality: Kapahulu Rd.; **Identification:** identifiedBy: ET Cresson; **Event:** verbatimEventDate: 26.x.1931; **Record Level:** institutionCode: BPBM
- **Type status:** Other material. **Occurrence:** recordedBy: no collector given; individualCount: 1; lifeStage: adult; **Location:** islandGroup: Hawaiian Islands; island: Hawaii; country: United States; stateProvince: Hawaii; county: Hawaii; verbatimLocality: Hilo substation; **Identification:** identifiedBy: WW Wirth; **Event:** verbatimEventDate: vii.1936; **Record Level:** institutionCode: BPBM
- **Type status:** Other material. **Occurrence:** recordedBy: EC Zimmerman; individualCount: 15; lifeStage: adult; **Location:** islandGroup: Hawaiian Islands; island: Kauai; country: United States; stateProvince: Hawaii; county: Kauai; verbatimLocality: Near Halemanu; **Identification:** identifiedBy: JA Tenorio; **Event:** verbatimEventDate: 10.vii.1937; **Record Level:** institutionCode: BPBM
- **Type status:** Other material. **Occurrence:** recordedBy: EH Bryan Jr.; individualCount: 1; lifeStage: adult; **Location:** islandGroup: Hawaiian Islands; island: Kauai; country: United States; stateProvince: Hawaii; county: Kauai; verbatimLocality: Honopu; **Identification:** identifiedBy: JA Tenorio; **Event:** verbatimEventDate: 17.vi.1922; **Record Level:** institutionCode: BPBM
- **Type status:** Other material. **Occurrence:** recordedBy: EH Bryan Jr.; individualCount: 4; lifeStage: adult; **Location:** islandGroup: Hawaiian Islands; island: Maui; country: United States; stateProvince: Hawaii; county: Maui; verbatimLocality: Wailuaiki; **Identification:** identifiedBy: ET Cresson; **Event:** verbatimEventDate: 3.vii.1920; **Record Level:** institutionCode: BPBM
- **Type status:** Other material. **Occurrence:** recordedBy: NLH Krauss; individualCount: 4; lifeStage: adult; **Location:** islandGroup: Hawaiian Islands; island: Maui; country: United States; stateProvince: Hawaii; county: Maui; verbatimLocality: Olawalu; **Identification:** identifiedBy: JA Tenorio; **Event:** verbatimEventDate: 21.iii.1967; **Record Level:** institutionCode: BPBM
- **Type status:** Other material. **Occurrence:** recordedBy: NLH Krauss; individualCount: 1; lifeStage: adult; **Location:** islandGroup: Hawaiian Islands; island: Maui; country: United States; stateProvince: Hawaii; county: Maui; verbatimLocality: Olinda; **Identification:** identifiedBy: JA Tenorio; **Event:** verbatimEventDate: 1.viii.1932; **Record Level:** institutionCode: BPBM
- **Type status:** Other material. **Occurrence:** recordedBy: EJ Ford; individualCount: 1; lifeStage: adult; **Location:** islandGroup: Hawaiian Islands; island: Molokai; country: United States; stateProvince: Hawaii; county: Maui; verbatimLocality: Waikolu Valley; verbatimElevation: 4593 ft.; **Identification:** identifiedBy: JA Tenorio; **Event:** verbatimEventDate: 1.v.1955; **Record Level:** institutionCode: BPBM
- **Type status:** Other material. **Occurrence:** recordedBy: NLH Krauss; individualCount: 2; lifeStage: adult; **Location:** islandGroup: Hawaiian Islands; island: Maui; country: United States; stateProvince: Hawaii; county: Maui; verbatimLocality: Ulupalakua; **Identification:** identifiedBy: JA Tenorio; **Event:** verbatimEventDate: 8.ii.1958; **Record Level:** institutionCode: BPBM
- **Type status:** Other material. **Occurrence:** recordedBy: NLH Krauss; individualCount: 1; lifeStage: adult; **Location:** islandGroup: Hawaiian Islands; island: Maui; country: United States; stateProvince: Hawaii; county: Maui; verbatimLocality: Kaupo; **Identification:** identifiedBy: JA Tenorio; **Event:** verbatimEventDate: ii.1958; **Record Level:** institutionCode: BPBM
- **Type status:** Other material. **Occurrence:** recordedBy: NLH Krauss; individualCount: 1; lifeStage: adult; **Location:** islandGroup: Hawaiian Islands; island: Maui; country: United States; stateProvince: Hawaii; county: Maui; verbatimLocality: Nahiku; **Identification:** identifiedBy: JA Tenorio; **Event:** verbatimEventDate: ii.1958; **Record Level:** institutionCode: BPBM
- **Type status:** Other material. **Occurrence:** recordedBy: LW Quate; individualCount: 1; lifeStage: adult; **Location:** islandGroup: Hawaiian Islands; island: Maui; country: United States; stateProvince: Hawaii; county: Maui; verbatimLocality: Waikamoi; verbatimElevation: 3936 ft.; **Identification:** identifiedBy: JA Tenorio; **Event:** verbatimEventDate: 14.iii.1961; **Record Level:** institutionCode: BPBM
- **Type status:** Other material. **Occurrence:** recordedBy: no collector given; individualCount: 1; lifeStage: adult; **Location:** islandGroup: Hawaiian Islands; island: Maui; country: United States; stateProvince: Hawaii; county: Maui; verbatimLocality: Haleakala; **Identification:** identifiedBy: JA Tenorio; **Event:** verbatimEventDate: no date given; **Record Level:** institutionCode: BPBM
- **Type status:** Other material. **Occurrence:** recordedBy: LW Quate; individualCount: 1; lifeStage: adult; **Location:** islandGroup: Hawaiian Islands; island: Hawaii; country: United States; stateProvince: Hawaii; county: Hawaii; verbatimLocality: Pololu Valley; **Identification:** identifiedBy: JA Tenorio; **Event:** verbatimEventDate: 2.viii.1958; **Record Level:** institutionCode: BPBM
- **Type status:** Other material. **Occurrence:** recordedBy: LW Quate; individualCount: 5; lifeStage: adult; **Location:** islandGroup: Hawaiian Islands; island: Hawaii; country: United States; stateProvince: Hawaii; county: Hawaii; verbatimLocality: Kohala Mts.; verbatimElevation: 1900 ft.; **Identification:** identifiedBy: JA Tenorio; **Event:** verbatimEventDate: 31.vii.1958; **Record Level:** institutionCode: BPBM
- **Type status:** Other material. **Occurrence:** recordedBy: LW Quate; individualCount: 2; lifeStage: adult; **Location:** islandGroup: Hawaiian Islands; island: Hawaii; country: United States; stateProvince: Hawaii; county: Hawaii; verbatimLocality: Head of Waipio Valley; verbatimElevation: 3936 ft.; **Identification:** identifiedBy: JA Tenorio; **Event:** verbatimEventDate: 23.iii.1961; **Record Level:** institutionCode: BPBM
- **Type status:** Other material. **Occurrence:** recordedBy: CP Hoyt; individualCount: 38; lifeStage: adult; **Location:** islandGroup: Hawaiian Islands; island: Oahu; country: United States; stateProvince: Hawaii; county: Honolulu; verbatimLocality: Honolulu, Lulumahu Valley; **Identification:** identifiedBy: JA Tenorio; **Event:** verbatimEventDate: 20.ii.1953; **Record Level:** institutionCode: BPBM
- **Type status:** Other material. **Occurrence:** recordedBy: CP Hoyt; individualCount: 1; lifeStage: adult; **Location:** islandGroup: Hawaiian Islands; island: Oahu; country: United States; stateProvince: Hawaii; county: Honolulu; verbatimLocality: Makapu; **Identification:** identifiedBy: JA Tenorio; **Event:** verbatimEventDate: 21.viii.1953; **Record Level:** institutionCode: BPBM
- **Type status:** Other material. **Occurrence:** recordedBy: NLH Krauss; individualCount: 2; lifeStage: adult; **Location:** islandGroup: Hawaiian Islands; island: Oahu; country: United States; stateProvince: Hawaii; county: Honolulu; **Identification:** identifiedBy: JA Tenorio; **Event:** verbatimEventDate: 1932; **Record Level:** institutionCode: BPBM
- **Type status:** Other material. **Occurrence:** recordedBy: RCA Rice; individualCount: 1; lifeStage: adult; **Location:** islandGroup: Hawaiian Islands; island: Maui; country: United States; stateProvince: Hawaii; county: Maui; verbatimLocality: Haleakala National Park, Kaupo Gap; verbatimElevation: 5400 ft.; **Identification:** identifiedBy: K Arakaki; **Event:** verbatimEventDate: 24.vi.1976; **Record Level:** institutionCode: BPBM
- **Type status:** Other material. **Occurrence:** recordedBy: WW Wirth; individualCount: 16; lifeStage: adult; **Location:** islandGroup: Hawaiian Islands; island: Hawaii; country: United States; stateProvince: Hawaii; county: Hawaii; verbatimLocality: Akaka Falls; **Event:** verbatimEventDate: 5.iii.1946; **Record Level:** institutionCode: UHM
- **Type status:** Other material. **Occurrence:** catalogNumber: USNMENT 00972811; recordedBy: WW Wirth; individualCount: 1; sex: female; lifeStage: adult; **Location:** islandGroup: Hawaiian Islands; island: Hawaii; country: United States; stateProvince: Hawaii; county: Hawaii; verbatimLocality: Akaka Falls; **Event:** verbatimEventDate: 5.iii.1946; **Record Level:** institutionCode: USNM
- **Type status:** Other material. **Occurrence:** catalogNumber: USNMENT 00972812; recordedBy: WW Wirth; individualCount: 1; sex: male; lifeStage: adult; **Location:** islandGroup: Hawaiian Islands; island: Hawaii; country: United States; stateProvince: Hawaii; county: Hawaii; verbatimLocality: Akaka Falls; **Event:** verbatimEventDate: 5.iii.1946; **Record Level:** institutionCode: USNM
- **Type status:** Other material. **Occurrence:** catalogNumber: USNMENT 00972813; recordedBy: WW Wirth; individualCount: 1; sex: female; lifeStage: adult; **Location:** islandGroup: Hawaiian Islands; island: Hawaii; country: United States; stateProvince: Hawaii; county: Hawaii; verbatimLocality: Akaka Falls; **Event:** verbatimEventDate: 5.iii.1946; **Record Level:** institutionCode: USNM
- **Type status:** Other material. **Occurrence:** catalogNumber: USNMENT 00972814; recordedBy: WW Wirth; individualCount: 1; sex: male; lifeStage: adult; **Location:** islandGroup: Hawaiian Islands; island: Hawaii; country: United States; stateProvince: Hawaii; county: Hawaii; verbatimLocality: Akaka Falls; **Event:** verbatimEventDate: 5.iii.1946; **Record Level:** institutionCode: USNM
- **Type status:** Other material. **Occurrence:** catalogNumber: USNMENT 00972815; recordedBy: WW Wirth; individualCount: 1; sex: male; lifeStage: adult; **Location:** islandGroup: Hawaiian Islands; island: Hawaii; country: United States; stateProvince: Hawaii; county: Hawaii; verbatimLocality: Akaka Falls; **Event:** verbatimEventDate: 5.iii.1946; **Record Level:** institutionCode: USNM
- **Type status:** Other material. **Occurrence:** catalogNumber: USNMENT 00972816; recordedBy: WW Wirth; individualCount: 1; sex: female; lifeStage: adult; **Location:** islandGroup: Hawaiian Islands; island: Hawaii; country: United States; stateProvince: Hawaii; county: Hawaii; verbatimLocality: Akaka Falls; **Event:** verbatimEventDate: 5.iii.1946; **Record Level:** institutionCode: USNM
- **Type status:** Other material. **Occurrence:** catalogNumber: USNMENT 00972817; recordedBy: WW Wirth; individualCount: 1; sex: male; lifeStage: adult; **Location:** islandGroup: Hawaiian Islands; island: Hawaii; country: United States; stateProvince: Hawaii; county: Hawaii; verbatimLocality: Akaka Falls; **Event:** verbatimEventDate: 5.iii.1946; **Record Level:** institutionCode: USNM
- **Type status:** Other material. **Occurrence:** catalogNumber: USNMENT 00972818; recordedBy: WW Wirth; individualCount: 1; sex: female; lifeStage: adult; **Location:** islandGroup: Hawaiian Islands; island: Hawaii; country: United States; stateProvince: Hawaii; county: Hawaii; verbatimLocality: Akaka Falls; **Event:** verbatimEventDate: 5.iii.1946; **Record Level:** institutionCode: USNM
- **Type status:** Other material. **Occurrence:** catalogNumber: USNMENT 00972819; recordedBy: WW Wirth; individualCount: 1; sex: female; lifeStage: adult; **Location:** islandGroup: Hawaiian Islands; island: Hawaii; country: United States; stateProvince: Hawaii; county: Hawaii; verbatimLocality: Akaka Falls; **Event:** verbatimEventDate: 5.iii.1946; **Record Level:** institutionCode: USNM
- **Type status:** Other material. **Occurrence:** catalogNumber: USNMENT 00972821; recordedBy: WW Wirth; individualCount: 1; sex: male; lifeStage: adult; **Location:** islandGroup: Hawaiian Islands; island: Hawaii; country: United States; stateProvince: Hawaii; county: Hawaii; verbatimLocality: Akaka Falls; **Event:** verbatimEventDate: 5.iii.1946; **Record Level:** institutionCode: USNM
- **Type status:** Other material. **Occurrence:** catalogNumber: USNMENT 00972822; recordedBy: WW Wirth; individualCount: 1; sex: male; lifeStage: adult; **Location:** islandGroup: Hawaiian Islands; island: Hawaii; country: United States; stateProvince: Hawaii; county: Hawaii; verbatimLocality: Akaka Falls; **Event:** verbatimEventDate: 5.iii.1946; **Record Level:** institutionCode: USNM
- **Type status:** Other material. **Occurrence:** catalogNumber: USNMENT 00972823; recordedBy: WW Wirth; individualCount: 1; sex: female; lifeStage: adult; **Location:** islandGroup: Hawaiian Islands; island: Hawaii; country: United States; stateProvince: Hawaii; county: Hawaii; verbatimLocality: Akaka Falls; **Event:** verbatimEventDate: 5.iii.1946; **Record Level:** institutionCode: USNM
- **Type status:** Other material. **Occurrence:** catalogNumber: USNMENT 00972824; recordedBy: WW Wirth; individualCount: 1; sex: male; lifeStage: adult; **Location:** islandGroup: Hawaiian Islands; island: Hawaii; country: United States; stateProvince: Hawaii; county: Hawaii; verbatimLocality: Akaka Falls; **Event:** verbatimEventDate: 5.iii.1946; **Record Level:** institutionCode: USNM
- **Type status:** Other material. **Occurrence:** catalogNumber: USNMENT 00972825; recordedBy: WW Wirth; individualCount: 1; sex: female; lifeStage: adult; **Location:** islandGroup: Hawaiian Islands; island: Hawaii; country: United States; stateProvince: Hawaii; county: Hawaii; verbatimLocality: Akaka Falls; **Event:** verbatimEventDate: 5.iii.1946; **Record Level:** institutionCode: USNM
- **Type status:** Other material. **Occurrence:** catalogNumber: USNMENT 00972826; recordedBy: WW Wirth; individualCount: 1; sex: female; lifeStage: adult; **Location:** islandGroup: Hawaiian Islands; island: Hawaii; country: United States; stateProvince: Hawaii; county: Hawaii; verbatimLocality: Akaka Falls; **Event:** verbatimEventDate: 5.iii.1946; **Record Level:** institutionCode: USNM
- **Type status:** Other material. **Occurrence:** catalogNumber: USNMENT 00972827; recordedBy: WW Wirth; individualCount: 1; sex: female; lifeStage: adult; **Location:** islandGroup: Hawaiian Islands; island: Hawaii; country: United States; stateProvince: Hawaii; county: Hawaii; verbatimLocality: Akaka Falls; **Event:** verbatimEventDate: 5.iii.1946; **Record Level:** institutionCode: USNM
- **Type status:** Other material. **Occurrence:** catalogNumber: USNMENT 00972828; recordedBy: WW Wirth; individualCount: 1; sex: female; lifeStage: adult; **Location:** islandGroup: Hawaiian Islands; island: Hawaii; country: United States; stateProvince: Hawaii; county: Hawaii; verbatimLocality: Akaka Falls; **Event:** verbatimEventDate: 5.iii.1946; **Record Level:** institutionCode: USNM
- **Type status:** Other material. **Occurrence:** catalogNumber: USNMENT 00972829; recordedBy: WW Wirth; individualCount: 1; sex: male; lifeStage: adult; **Location:** islandGroup: Hawaiian Islands; island: Hawaii; country: United States; stateProvince: Hawaii; county: Hawaii; verbatimLocality: Akaka Falls; **Event:** verbatimEventDate: 5.iii.1946; **Record Level:** institutionCode: USNM
- **Type status:** Other material. **Occurrence:** catalogNumber: USNMENT 00972830; recordedBy: WW Wirth; individualCount: 1; sex: female; lifeStage: adult; **Location:** islandGroup: Hawaiian Islands; island: Hawaii; country: United States; stateProvince: Hawaii; county: Hawaii; verbatimLocality: Akaka Falls; **Event:** verbatimEventDate: 5.iii.1946; **Record Level:** institutionCode: USNM
- **Type status:** Other material. **Occurrence:** catalogNumber: USNMENT 00972831; recordedBy: WW Wirth; individualCount: 1; sex: male; lifeStage: adult; **Location:** islandGroup: Hawaiian Islands; island: Hawaii; country: United States; stateProvince: Hawaii; county: Hawaii; verbatimLocality: Akaka Falls; **Event:** verbatimEventDate: 5.iii.1946; **Record Level:** institutionCode: USNM
- **Type status:** Other material. **Occurrence:** catalogNumber: USNMENT 00972832; recordedBy: WW Wirth; individualCount: 1; sex: female; lifeStage: adult; **Location:** islandGroup: Hawaiian Islands; island: Hawaii; country: United States; stateProvince: Hawaii; county: Hawaii; verbatimLocality: Akaka Falls; **Event:** verbatimEventDate: 5.iii.1946; **Record Level:** institutionCode: USNM
- **Type status:** Other material. **Occurrence:** catalogNumber: USNMENT 00972833; recordedBy: WW Wirth; individualCount: 1; sex: male; lifeStage: adult; **Location:** islandGroup: Hawaiian Islands; island: Hawaii; country: United States; stateProvince: Hawaii; county: Hawaii; verbatimLocality: Akaka Falls; **Event:** verbatimEventDate: 5.iii.1946; **Record Level:** institutionCode: USNM
- **Type status:** Other material. **Occurrence:** catalogNumber: USNMENT 00972834; recordedBy: WW Wirth; individualCount: 1; sex: female; lifeStage: adult; **Location:** islandGroup: Hawaiian Islands; island: Hawaii; country: United States; stateProvince: Hawaii; county: Hawaii; verbatimLocality: Akaka Falls; **Event:** verbatimEventDate: 5.iii.1946; **Record Level:** institutionCode: USNM
- **Type status:** Other material. **Occurrence:** catalogNumber: USNMENT 00972835; recordedBy: WW Wirth; individualCount: 1; sex: female; lifeStage: adult; **Location:** islandGroup: Hawaiian Islands; island: Hawaii; country: United States; stateProvince: Hawaii; county: Hawaii; verbatimLocality: Akaka Falls; **Event:** verbatimEventDate: 5.iii.1946; **Record Level:** institutionCode: USNM
- **Type status:** Other material. **Occurrence:** catalogNumber: USNMENT 00972836; recordedBy: WW Wirth; individualCount: 1; sex: female; lifeStage: adult; **Location:** islandGroup: Hawaiian Islands; island: Hawaii; country: United States; stateProvince: Hawaii; county: Hawaii; verbatimLocality: Akaka Falls; **Event:** verbatimEventDate: 5.iii.1946; **Record Level:** institutionCode: USNM
- **Type status:** Other material. **Occurrence:** catalogNumber: USNMENT 00972837; recordedBy: WW Wirth; individualCount: 1; sex: male; lifeStage: adult; **Location:** islandGroup: Hawaiian Islands; island: Hawaii; country: United States; stateProvince: Hawaii; county: Hawaii; verbatimLocality: Akaka Falls; **Event:** verbatimEventDate: 5.iii.1946; **Record Level:** institutionCode: USNM
- **Type status:** Other material. **Occurrence:** catalogNumber: USNMENT 00972838; recordedBy: WW Wirth; individualCount: 1; sex: male; lifeStage: adult; **Location:** islandGroup: Hawaiian Islands; island: Hawaii; country: United States; stateProvince: Hawaii; county: Hawaii; verbatimLocality: Akaka Falls; **Event:** verbatimEventDate: 5.iii.1946; **Record Level:** institutionCode: USNM
- **Type status:** Other material. **Occurrence:** catalogNumber: USNMENT 00972801; recordedBy: WW Wirth; individualCount: 1; sex: female; lifeStage: adult; **Location:** islandGroup: Hawaiian Islands; island: Hawaii; country: United States; stateProvince: Hawaii; county: Hawaii; verbatimLocality: Rainbow Falls, Hilo; **Event:** verbatimEventDate: 3.iii.1946; **Record Level:** institutionCode: USNM
- **Type status:** Other material. **Occurrence:** catalogNumber: USNMENT 00972802; recordedBy: WW Wirth; individualCount: 1; sex: female; lifeStage: adult; **Location:** islandGroup: Hawaiian Islands; island: Hawaii; country: United States; stateProvince: Hawaii; county: Hawaii; verbatimLocality: Rainbow Falls, Hilo; **Event:** verbatimEventDate: 3.iii.1946; **Record Level:** institutionCode: USNM
- **Type status:** Other material. **Occurrence:** catalogNumber: USNMENT 00972803; recordedBy: WW Wirth; individualCount: 1; sex: male; lifeStage: adult; **Location:** islandGroup: Hawaiian Islands; island: Hawaii; country: United States; stateProvince: Hawaii; county: Hawaii; verbatimLocality: Rainbow Falls, Hilo; **Event:** verbatimEventDate: 3.iii.1946; **Record Level:** institutionCode: USNM
- **Type status:** Other material. **Occurrence:** catalogNumber: USNMENT 00972804; recordedBy: WW Wirth; individualCount: 1; sex: male; lifeStage: adult; **Location:** islandGroup: Hawaiian Islands; island: Hawaii; country: United States; stateProvince: Hawaii; county: Hawaii; verbatimLocality: Rainbow Falls, Hilo; **Event:** verbatimEventDate: 3.iii.1946; **Record Level:** institutionCode: USNM
- **Type status:** Other material. **Occurrence:** catalogNumber: USNMENT 00972805; recordedBy: WW Wirth; individualCount: 1; sex: female; lifeStage: adult; **Location:** islandGroup: Hawaiian Islands; island: Hawaii; country: United States; stateProvince: Hawaii; county: Hawaii; verbatimLocality: Rainbow Falls, Hilo; **Event:** verbatimEventDate: 3.iii.1946; **Record Level:** institutionCode: USNM
- **Type status:** Other material. **Occurrence:** catalogNumber: USNMENT 00972806; recordedBy: WW Wirth; individualCount: 1; sex: female; lifeStage: adult; **Location:** islandGroup: Hawaiian Islands; island: Hawaii; country: United States; stateProvince: Hawaii; county: Hawaii; verbatimLocality: Rainbow Falls, Hilo; **Event:** verbatimEventDate: 3.iii.1946; **Record Level:** institutionCode: USNM
- **Type status:** Other material. **Occurrence:** catalogNumber: USNMENT 00972807; recordedBy: WW Wirth; individualCount: 1; sex: female; lifeStage: adult; **Location:** islandGroup: Hawaiian Islands; island: Hawaii; country: United States; stateProvince: Hawaii; county: Hawaii; verbatimLocality: Rainbow Falls, Hilo; **Event:** verbatimEventDate: 3.iii.1946; **Record Level:** institutionCode: USNM
- **Type status:** Other material. **Occurrence:** catalogNumber: USNMENT 00972808; recordedBy: WW Wirth; individualCount: 1; sex: male; lifeStage: adult; **Location:** islandGroup: Hawaiian Islands; island: Hawaii; country: United States; stateProvince: Hawaii; county: Hawaii; verbatimLocality: Rainbow Falls, Hilo; **Event:** verbatimEventDate: 3.iii.1946; **Record Level:** institutionCode: USNM
- **Type status:** Other material. **Occurrence:** catalogNumber: USNMENT 00972809; recordedBy: WW Wirth; individualCount: 1; sex: male; lifeStage: adult; **Location:** islandGroup: Hawaiian Islands; island: Hawaii; country: United States; stateProvince: Hawaii; county: Hawaii; verbatimLocality: Rainbow Falls, Hilo; **Event:** verbatimEventDate: 3.iii.1946; **Record Level:** institutionCode: USNM
- **Type status:** Other material. **Occurrence:** catalogNumber: USNMENT 00972810; recordedBy: WW Wirth; individualCount: 1; sex: female; lifeStage: adult; **Location:** islandGroup: Hawaiian Islands; island: Hawaii; country: United States; stateProvince: Hawaii; county: Hawaii; verbatimLocality: Rainbow Falls, Hilo; **Event:** verbatimEventDate: 3.iii.1946; **Record Level:** institutionCode: USNM
- **Type status:** Other material. **Occurrence:** recordedBy: FX Williams; individualCount: 2; lifeStage: adult; **Location:** islandGroup: Hawaiian Islands; island: Hawaii; country: United States; stateProvince: Hawaii; county: Hawaii; verbatimLocality: Akaka Falls; **Event:** verbatimEventDate: 21.x.1931; **Record Level:** institutionCode: UHM
- **Type status:** Other material. **Occurrence:** recordedBy: FX Williams; individualCount: 9; lifeStage: adult; **Location:** islandGroup: Hawaiian Islands; island: Hawaii; country: United States; stateProvince: Hawaii; county: Hawaii; verbatimLocality: Hilo, flume; **Event:** verbatimEventDate: vii.1936; **Record Level:** institutionCode: UHM
- **Type status:** Other material. **Occurrence:** recordedBy: JF Illingworth; individualCount: 2; lifeStage: adult; **Location:** islandGroup: Hawaiian Islands; island: Maui; country: United States; stateProvince: Hawaii; county: Maui; verbatimLocality: Iao Valley; **Event:** verbatimEventDate: 3.i.1915; **Record Level:** institutionCode: UHM
- **Type status:** Other material. **Occurrence:** recordedBy: EH Bryan Jr.; individualCount: 1; sex: male; lifeStage: adult; **Location:** islandGroup: Hawaiian Islands; island: Maui; country: United States; stateProvince: Hawaii; county: Maui; verbatimLocality: Kailua; **Event:** verbatimEventDate: 20.vi.1920; **Record Level:** institutionCode: UHM
- **Type status:** Other material. **Occurrence:** recordedBy: EH Bryan Jr.; individualCount: 1; sex: male; lifeStage: adult; **Location:** islandGroup: Hawaiian Islands; island: Maui; country: United States; stateProvince: Hawaii; county: Maui; verbatimLocality: Wailua-iki; **Event:** verbatimEventDate: 3.vii.1920; **Record Level:** institutionCode: UHM
- **Type status:** Other material. **Occurrence:** recordedBy: WW Wirth; individualCount: 1; sex: male; lifeStage: adult; previousIdentifications: holotype of N. fimbriata; **Location:** islandGroup: Hawaiian Islands; island: Oahu; country: United States; stateProvince: Hawaii; county: Honolulu; verbatimLocality: Palolo Valley; verbatimElevation: 1500 ft.; **Event:** verbatimEventDate: 19.i.1946; **Record Level:** institutionCode: BPBM
- **Type status:** Other material. **Occurrence:** recordedBy: WW Wirth; individualCount: 1; sex: female; lifeStage: adult; previousIdentifications: allotype of N. fimbriata; **Location:** islandGroup: Hawaiian Islands; island: Oahu; country: United States; stateProvince: Hawaii; county: Honolulu; verbatimLocality: Kaluanui Valley, at stream; verbatimElevation: 2000 ft.; **Event:** verbatimEventDate: 14.v.1946; **Record Level:** institutionCode: BPBM
- **Type status:** Other material. **Occurrence:** catalogNumber: USNMENT 00972799; recordedBy: WW Wirth; individualCount: 1; sex: male; lifeStage: adult; **Location:** islandGroup: Hawaiian Islands; island: Oahu; country: United States; stateProvince: Hawaii; county: Honolulu; verbatimLocality: Kaluanui Valley, at stream; verbatimElevation: 2000 ft.; **Event:** verbatimEventDate: 14.v.1946; **Record Level:** institutionCode: USNM
- **Type status:** Other material. **Occurrence:** catalogNumber: USNMENT 00972800; recordedBy: WW Wirth; individualCount: 1; sex: female; lifeStage: adult; **Location:** islandGroup: Hawaiian Islands; island: Oahu; country: United States; stateProvince: Hawaii; county: Honolulu; verbatimLocality: Kaluanui Valley, at stream; verbatimElevation: 2000 ft.; **Event:** verbatimEventDate: 14.v.1946; **Record Level:** institutionCode: USNM
- **Type status:** Other material. **Occurrence:** recordedBy: WW Wirth; individualCount: 1; sex: male; lifeStage: adult; previousIdentifications: paratype of N. fimbriata; **Location:** islandGroup: Hawaiian Islands; island: Oahu; country: United States; stateProvince: Hawaii; county: Honolulu; verbatimLocality: Manoa Valley; **Event:** verbatimEventDate: 6.v.1945; **Record Level:** institutionCode: BPBM
- **Type status:** Other material. **Occurrence:** catalogNumber: USNMENT 00972798; recordedBy: WW Wirth; individualCount: 1; sex: male; lifeStage: adult; **Location:** islandGroup: Hawaiian Islands; island: Oahu; country: United States; stateProvince: Hawaii; county: Honolulu; verbatimLocality: Manoa Valley; **Event:** verbatimEventDate: 6.v.1945; **Record Level:** institutionCode: USNM
- **Type status:** Other material. **Occurrence:** recordedBy: WW Wirth; individualCount: 3; lifeStage: adult; previousIdentifications: paratype of N. fimbriata; **Location:** islandGroup: Hawaiian Islands; island: Oahu; country: United States; stateProvince: Hawaii; county: Honolulu; verbatimLocality: Kaluanui Valley, at stream; verbatimElevation: 2000 ft.; **Event:** verbatimEventDate: 14.v.1946; **Record Level:** institutionCode: BPBM
- **Type status:** Other material. **Occurrence:** recordedBy: WW Wirth; individualCount: 1; sex: male; lifeStage: adult; previousIdentifications: paratype of N. fimbriata; **Location:** islandGroup: Hawaiian Islands; island: Oahu; country: United States; stateProvince: Hawaii; county: Honolulu; verbatimLocality: Mt. Kaala; **Event:** verbatimEventDate: 17.iv.1946; **Record Level:** institutionCode: BPBM
- **Type status:** Other material. **Occurrence:** recordedBy: EH Bryan Jr.; individualCount: 6; lifeStage: adult; previousIdentifications: paratype of N. fimbriata; **Location:** islandGroup: Hawaiian Islands; island: Oahu; country: United States; stateProvince: Hawaii; county: Honolulu; verbatimLocality: Makaleha; **Identification:** identifiedBy: WW Wirth; **Event:** verbatimEventDate: 13.i.1929; **Record Level:** institutionCode: BPBM
- **Type status:** Other material. **Occurrence:** recordedBy: FX Williams; individualCount: 3; lifeStage: adult; previousIdentifications: paratype of N. fimbriata; **Location:** islandGroup: Hawaiian Islands; island: Oahu; country: United States; stateProvince: Hawaii; county: Honolulu; verbatimLocality: Waianae, swift water ditch; **Event:** verbatimEventDate: 10.v.1936; **Record Level:** institutionCode: UHM
- **Type status:** Other material. **Occurrence:** recordedBy: FX Williams; individualCount: 4; sex: female; lifeStage: adult; **Location:** islandGroup: Hawaiian Islands; island: Oahu; country: United States; stateProvince: Hawaii; county: Honolulu; verbatimLocality: Kalihi Stream, Honolulu, wet boulder; **Event:** verbatimEventDate: 13-14.v.1937; **Record Level:** institutionCode: UHM
- **Type status:** Other material. **Occurrence:** recordedBy: EH Bryan Jr.; individualCount: 8; lifeStage: adult; **Location:** islandGroup: Hawaiian Islands; island: Oahu; country: United States; stateProvince: Hawaii; county: Honolulu; verbatimLocality: Moanalua; **Event:** verbatimEventDate: 9.iv.1922; **Record Level:** institutionCode: UHM
- **Type status:** Other material. **Occurrence:** recordedBy: EH Bryan Jr.; individualCount: 1; sex: female; lifeStage: adult; **Location:** islandGroup: Hawaiian Islands; island: Oahu; country: United States; stateProvince: Hawaii; county: Honolulu; verbatimLocality: Palolo; **Event:** verbatimEventDate: 25.ii.1922; **Record Level:** institutionCode: UHM

##### Ecological interactions

###### Native status

Endemic.

##### Distribution

Hawaiian Islands: Kauai, Oahu, Molokai, Maui, Hawaii.

##### Notes

32 specimens from USNM not examined.

#### Scatella williamsi

(Wirth, 1948)

##### Materials

- **Type status:** Holotype. **Occurrence:** recordedBy: WW Wirth; individualCount: 1; sex: female; lifeStage: adult; **Location:** islandGroup: Hawaiian Islands; island: Hawaii; country: United States; stateProvince: Hawaii; county: Hawaii; verbatimLocality: Akaka Falls, Hilo; **Event:** verbatimEventDate: 5.iii.1946; **Record Level:** institutionCode: BPBM
- **Type status:** Paratype. **Occurrence:** recordedBy: WW Wirth; individualCount: 1; sex: male; lifeStage: adult; **Location:** islandGroup: Hawaiian Islands; island: Hawaii; country: United States; stateProvince: Hawaii; county: Hawaii; verbatimLocality: Akaka Falls, Hilo; **Event:** verbatimEventDate: 5.iii.1946; **Record Level:** institutionCode: BPBM
- **Type status:** Paratype. **Occurrence:** recordedBy: WW Wirth; individualCount: 7; lifeStage: adult; **Location:** islandGroup: Hawaiian Islands; island: Hawaii; country: United States; stateProvince: Hawaii; county: Hawaii; verbatimLocality: Akaka Falls, Hilo; **Event:** verbatimEventDate: 5.iii.1946; **Record Level:** institutionCode: BPBM
- **Type status:** Other material. **Occurrence:** recordedBy: FX Williams; individualCount: 1; lifeStage: adult; **Location:** islandGroup: Hawaiian Islands; island: Hawaii; country: United States; stateProvince: Hawaii; county: Hawaii; verbatimLocality: Akaka Falls, about water; **Identification:** identifiedBy: WW Wirth; **Event:** verbatimEventDate: 24.x.1931; **Record Level:** institutionCode: BPBM
- **Type status:** Other material. **Occurrence:** recordedBy: LW Quate; individualCount: 1; lifeStage: adult; **Location:** islandGroup: Hawaiian Islands; island: Hawaii; country: United States; stateProvince: Hawaii; county: Hawaii; verbatimLocality: Kohala Mts.; verbatimElevation: 1900 ft.; **Identification:** identifiedBy: JA Tenorio; **Event:** verbatimEventDate: 31.vii.1958; **Record Level:** institutionCode: BPBM
- **Type status:** Other material. **Occurrence:** recordedBy: LW Quate; individualCount: 4; lifeStage: adult; **Location:** islandGroup: Hawaiian Islands; island: Hawaii; country: United States; stateProvince: Hawaii; county: Hawaii; verbatimLocality: Head of Waipio Valley; verbatimElevation: 3936 ft.; **Identification:** identifiedBy: JA Tenorio; **Event:** verbatimEventDate: 23.iii.1961; **Record Level:** institutionCode: BPBM
- **Type status:** Other material. **Occurrence:** recordedBy: JA Tenorio; individualCount: 4; lifeStage: adult; **Location:** islandGroup: Hawaiian Islands; island: Hawaii; country: United States; stateProvince: Hawaii; county: Hawaii; verbatimLocality: Akaka Falls; **Identification:** identifiedBy: JA Tenorio; **Event:** verbatimEventDate: 28.v.1970; **Record Level:** institutionCode: BPBM
- **Type status:** Other material. **Occurrence:** catalogNumber: EMEC7356075; recordedBy: PM O'Grady, RT Lapoint, GM Bennett, BS Ort, NA Pantoja; lifeStage: adult; **Location:** islandGroup: Hawaiian Islands; island: Hawaii; country: United States; stateProvince: Hawaii; county: Hawaii; verbatimLocality: 0.5 miles from start of scenic drive to Onomea Bay, first major bridge, sweeping along stream; verbatimLatitude: 19°47'42.20"N; verbatimLongitude: 155°5'38.58"W; geodeticDatum: WGS84; **Identification:** identifiedBy: PM O'Grady; **Event:** verbatimEventDate: 2.viii.2010; **Record Level:** institutionCode: EMEC; collectionCode: 613.4

##### Ecological interactions

###### Native status

Endemic.

##### Distribution

Hawaiian Islands: Hawaii.

#### Scatella wirthi

Tenorio, 1980

##### Materials

- **Type status:** Holotype. **Occurrence:** catalogNumber: 9478; recordedBy: CP Hoyt; individualCount: 1; sex: male; lifeStage: adult; **Location:** islandGroup: Hawaiian Islands; island: Hawaii; country: United States; stateProvince: Hawaii; county: Hawaii; verbatimLocality: Keanakolu, Kaula Gulch; verbatimElevation: 7000 ft.; **Event:** verbatimEventDate: 29.x.1952; **Record Level:** institutionCode: BPBM
- **Type status:** Paratype. **Occurrence:** recordedBy: O Bryant; lifeStage: adult; **Location:** islandGroup: Hawaiian Islands; island: Maui; country: United States; stateProvince: Hawaii; county: Maui; verbatimLocality: Kula Pipe Line; verbatimElevation: 4600-5000 ft.; **Event:** verbatimEventDate: 15.iii.1932; **Record Level:** institutionCode: UHM
- **Type status:** Paratype. **Occurrence:** recordedBy: O Bryant; lifeStage: adult; **Location:** islandGroup: Hawaiian Islands; island: Maui; country: United States; stateProvince: Hawaii; county: Maui; verbatimLocality: Haleakala; **Event:** verbatimEventDate: 23.iii.1932; **Record Level:** institutionCode: UHM
- **Type status:** Paratype. **Occurrence:** recordedBy: RL Usinger; lifeStage: adult; **Location:** islandGroup: Hawaiian Islands; island: Hawaii; country: United States; stateProvince: Hawaii; county: Hawaii; verbatimLocality: Hamuula; **Event:** verbatimEventDate: 7.vii.1935; **Record Level:** institutionCode: UHM
- **Type status:** Paratype. **Occurrence:** recordedBy: DE Hardy; lifeStage: adult; **Location:** islandGroup: Hawaiian Islands; island: Hawaii; country: United States; stateProvince: Hawaii; county: Hawaii; verbatimLocality: Kilauea; **Event:** verbatimEventDate: viii.1949; **Record Level:** institutionCode: UHM
- **Type status:** Paratype. **Occurrence:** recordedBy: DE Hardy; lifeStage: adult; **Location:** islandGroup: Hawaiian Islands; island: Hawaii; country: United States; stateProvince: Hawaii; county: Hawaii; verbatimLocality: Pauahi; **Event:** verbatimEventDate: 12.viii.1949; **Record Level:** institutionCode: UHM
- **Type status:** Paratype. **Occurrence:** recordedBy: E Dresner; individualCount: 2; lifeStage: adult; **Location:** islandGroup: Hawaiian Islands; island: Hawaii; country: United States; stateProvince: Hawaii; county: Hawaii; verbatimLocality: Lake Waiau, Mauna Kea; **Event:** verbatimEventDate: x.1951; **Record Level:** institutionCode: BPBM
- **Type status:** Paratype. **Occurrence:** recordedBy: DE Hardy; individualCount: 1; lifeStage: adult; **Location:** islandGroup: Hawaiian Islands; island: Hawaii; country: United States; stateProvince: Hawaii; county: Hawaii; verbatimLocality: Kulani; verbatimElevation: 5200 ft.; **Event:** verbatimEventDate: vii.1952; **Record Level:** institutionCode: BPBM
- **Type status:** Paratype. **Occurrence:** recordedBy: WC Mitchel; individualCount: 1; lifeStage: adult; **Location:** islandGroup: Hawaiian Islands; island: Hawaii; country: United States; stateProvince: Hawaii; county: Hawaii; verbatimLocality: Kulani; verbatimElevation: 5200 ft.; **Event:** verbatimEventDate: vii.1952; **Record Level:** institutionCode: BPBM
- **Type status:** Paratype. **Occurrence:** recordedBy: DE Hardy; lifeStage: adult; **Location:** islandGroup: Hawaiian Islands; island: Maui; country: United States; stateProvince: Hawaii; county: Maui; verbatimLocality: Paliku, Haleakala; verbatimElevation: 6000 ft.; **Event:** verbatimEventDate: vii.1952; **Record Level:** institutionCode: UHM
- **Type status:** Paratype. **Occurrence:** recordedBy: DE Hardy; lifeStage: adult; **Location:** islandGroup: Hawaiian Islands; island: Hawaii; country: United States; stateProvince: Hawaii; county: Hawaii; verbatimLocality: Puu Kalepa Pond; verbatimElevation: 8000 ft.; **Event:** verbatimEventDate: x.1952; **Record Level:** institutionCode: UHM
- **Type status:** Paratype. **Occurrence:** catalogNumber: 9478a; recordedBy: CP Hoyt; individualCount: 1; sex: female; lifeStage: adult; **Location:** islandGroup: Hawaiian Islands; island: Hawaii; country: United States; stateProvince: Hawaii; county: Hawaii; verbatimLocality: Keanakolu, Kaula Gulch; verbatimElevation: 7000 ft.; **Event:** verbatimEventDate: 29.x.1952; **Record Level:** institutionCode: BPBM
- **Type status:** Paratype. **Occurrence:** recordedBy: CP Hoyt; individualCount: 2; lifeStage: adult; **Location:** islandGroup: Hawaiian Islands; island: Hawaii; country: United States; stateProvince: Hawaii; county: Hawaii; verbatimLocality: Keanakolu, Kaula Gulch; verbatimElevation: 7000 ft.; **Event:** verbatimEventDate: 29.x.1952; **Record Level:** institutionCode: BPBM
- **Type status:** Paratype. **Occurrence:** recordedBy: CP Hoyt; individualCount: 9; lifeStage: adult; **Location:** islandGroup: Hawaiian Islands; island: Hawaii; country: United States; stateProvince: Hawaii; county: Hawaii; verbatimLocality: Keanakolu, Kaula Gulch; verbatimElevation: 7000 ft.; **Event:** verbatimEventDate: 29.x.1952; **Record Level:** institutionCode: UHM
- **Type status:** Paratype. **Occurrence:** recordedBy: DE Hardy; lifeStage: adult; **Location:** islandGroup: Hawaiian Islands; island: Maui; country: United States; stateProvince: Hawaii; county: Maui; verbatimLocality: Holua; verbatimElevation: 6500 ft.; **Event:** verbatimEventDate: vi.1953; **Record Level:** institutionCode: UHM
- **Type status:** Paratype. **Occurrence:** recordedBy: M Tamashiro; individualCount: 1; lifeStage: adult; **Location:** islandGroup: Hawaiian Islands; island: Maui; country: United States; stateProvince: Hawaii; county: Maui; verbatimLocality: Puu Nianiau; verbatimElevation: 7000 ft.; **Event:** verbatimEventDate: iv.1954; **Record Level:** institutionCode: BPBM
- **Type status:** Paratype. **Occurrence:** recordedBy: NLH Krauss; lifeStage: adult; **Location:** islandGroup: Hawaiian Islands; island: Maui; country: United States; stateProvince: Hawaii; county: Maui; verbatimLocality: Haiku; **Event:** verbatimEventDate: iii.1956; **Record Level:** institutionCode: UHM
- **Type status:** Paratype. **Occurrence:** recordedBy: R Namba, DE Hardy; lifeStage: adult; **Location:** islandGroup: Hawaiian Islands; island: Maui; country: United States; stateProvince: Hawaii; county: Maui; verbatimLocality: Puu Nianiau; verbatimElevation: 7000 ft.; **Event:** verbatimEventDate: vii.1956; **Record Level:** institutionCode: UHM
- **Type status:** Paratype. **Occurrence:** recordedBy: DE Hardy; lifeStage: adult; **Location:** islandGroup: Hawaiian Islands; island: Hawaii; country: United States; stateProvince: Hawaii; county: Hawaii; verbatimLocality: Upper Olaa Forest; verbatimElevation: 4000 ft.; **Event:** verbatimEventDate: vii.1956; **Record Level:** institutionCode: UHM
- **Type status:** Paratype. **Occurrence:** recordedBy: JW Beardsley; individualCount: 1; lifeStage: adult; **Location:** islandGroup: Hawaiian Islands; island: Maui; country: United States; stateProvince: Hawaii; county: Maui; verbatimLocality: Haleakala; verbatimElevation: 10000 ft.; **Event:** verbatimEventDate: ix.1956; **Record Level:** institutionCode: BPBM
- **Type status:** Paratype. **Occurrence:** recordedBy: DE Hardy; lifeStage: adult; **Location:** islandGroup: Hawaiian Islands; island: Hawaii; country: United States; stateProvince: Hawaii; county: Hawaii; verbatimLocality: Hualalei; **Event:** verbatimEventDate: 19.x.1963; **Record Level:** institutionCode: UHM
- **Type status:** Paratype. **Occurrence:** recordedBy: JW Beardsley; lifeStage: adult; **Location:** islandGroup: Hawaiian Islands; island: Maui; country: United States; stateProvince: Hawaii; county: Maui; verbatimLocality: Oili Puu, Haleakala; **Event:** verbatimEventDate: 23.vii.1965; **Record Level:** institutionCode: UHM
- **Type status:** Paratype. **Occurrence:** recordedBy: JA Tenorio; lifeStage: adult; **Location:** islandGroup: Hawaiian Islands; island: Maui; country: United States; stateProvince: Hawaii; county: Maui; verbatimLocality: Paliku, Haleakala, sweeping over mud; **Event:** verbatimEventDate: 12.ix.1968; **Record Level:** institutionCode: UHM
- **Type status:** Other material. **Occurrence:** recordedBy: WM Giffard; individualCount: 3; lifeStage: adult; **Location:** islandGroup: Hawaiian Islands; island: Hawaii; country: United States; stateProvince: Hawaii; county: Hawaii; verbatimLocality: 29 mi Olaa; **Identification:** identifiedBy: JA Tenorio; **Event:** verbatimEventDate: viii.1925; **Record Level:** institutionCode: BPBM
- **Type status:** Other material. **Occurrence:** recordedBy: RL Usinger; individualCount: 2; lifeStage: adult; **Location:** islandGroup: Hawaiian Islands; island: Hawaii; country: United States; stateProvince: Hawaii; county: Hawaii; verbatimLocality: Humuula; **Identification:** identifiedBy: JA Tenorio; **Event:** verbatimEventDate: 7.viii.1935; **Record Level:** institutionCode: BPBM
- **Type status:** Other material. **Occurrence:** recordedBy: RL Usinger; individualCount: 1; lifeStage: adult; **Location:** islandGroup: Hawaiian Islands; island: Hawaii; country: United States; stateProvince: Hawaii; county: Hawaii; verbatimLocality: Hookomo; **Identification:** identifiedBy: JA Tenorio; **Event:** verbatimEventDate: 9.vii.1935; **Record Level:** institutionCode: BPBM
- **Type status:** Other material. **Occurrence:** recordedBy: LW Quate; individualCount: 1; lifeStage: adult; **Location:** islandGroup: Hawaiian Islands; island: Maui; country: United States; stateProvince: Hawaii; county: Maui; verbatimLocality: Haleakala Road; verbatimElevation: 6396 ft.; **Identification:** identifiedBy: JA Tenorio; **Event:** verbatimEventDate: 15.iii.1951; **Record Level:** institutionCode: BPBM
- **Type status:** Other material. **Occurrence:** recordedBy: CP Hoyt; individualCount: 1; lifeStage: adult; **Location:** islandGroup: Hawaiian Islands; island: Hawaii; country: United States; stateProvince: Hawaii; county: Hawaii; verbatimLocality: Kaluakauka, Keanakolu Trail; verbatimElevation: 5000 ft.; **Identification:** identifiedBy: JA Tenorio; **Event:** verbatimEventDate: 30.x.1952; **Record Level:** institutionCode: BPBM
- **Type status:** Other material. **Occurrence:** recordedBy: S Kimoto; individualCount: 1; lifeStage: adult; **Location:** islandGroup: Hawaiian Islands; island: Hawaii; country: United States; stateProvince: Hawaii; county: Hawaii; verbatimLocality: Mt. Hualalai, above Captain Cook; verbatimElevation: 4000 ft.; **Identification:** identifiedBy: JA Tenorio; **Event:** verbatimEventDate: 12.v.1959; **Record Level:** institutionCode: BPBM

##### Ecological interactions

###### Native status

Endemic.

##### Distribution

Hawaiian Islands: Maui, Hawaii.

## Discussion

Although Ephydridae represent only a small percentage of the Diptera diversity present in the Hawaiian Islands, these taxa are notable for exploiting aquatic environments ranging from marine to brackish swamps to high-elevation freshwater streams. Hawaiian *Scatella* feed on algae and microorganisms, and can be found in a range of habitats, from stagnant water in the lowlands or clean fast-running mountain streams (Tenorio 1980). Larvae of species inhabiting swift-flowing streams possess strong spines on the posterior spiracular tubes that enable the larvae to cling onto substrates of algae and rocks so they would not be dislodged by the rushing water (Tenorio 1980). In contrast, larvae of species that inhabit calm, stagnant waters do not exhibit these spines, but instead have palmate “hydrofuge” hairs surrounding the posterior spiracular openings (Tenorio 1980). When these species forage for food on the water surface, the palmate hairs spread out over the water, break the water tension, and allow the spiracles to have direct contact with the air (Tenorio 1980). It is possible that many species of *Scatella*, because of their ancestral ability to tolerate saline environments, can disperse across the ocean channels surrounding the main Hawaiian Islands. Because different species tend to exhibit different morphological characteristics and occupy different niche when they exist in the same area, *Scatella* may be another example of adaptive radiation, albeit a smaller example, in Hawaii.

Thirteen of the 17 species we treat here are widespread and found on multiple islands. This is similar to what is seen in the endemic crane flies (*Dicranomyia*; Goodman and O'Grady 2013, Nitta and O’Grady 2008) and may be a general pattern for aquatic taxa with tolerance to salt water environments and a correspondingly higher dispersal ability than those species without such adaptations. Four species in the genus *Scatella*, *Scatella cinereifacies*, *Scatella fluvialis*, *Scatella kauaiensis*, and *Scatella terryi*, are single island endemics. Three of these are found at high elevations in association with fresh water, suggesting that dispersal ability has been lost or is much reduced in these species. Of the single island endemics, only *Scatella terryi* is found at lower elevations in association with salt water. There are only a few known collection records of this species, all of which were made prior to 1950. It is possible that it exists on other islands but has never been collected because it is rare. It is also possible that this is truly a narrow endemic and may have been driven to the point of extinction by human activity. Many of these are in areas that are subject to high use by humans (*e.g.*, Hanauma Bay, Waimanalo, Koko Crater). Additional collections are needed to fully assess the status of this taxon.

## Supplementary Material

Supplementary material 1 Distribution of Scatella amnica Data type: occurence
Brief description: Distribution of *Scatella amnica*. Samples with GPS coordinates shown in white; georeferenced historical samples shown in red.
File: oo_8578.kml PM O'Grady

Supplementary material 2 Distribution of Scatella bryani Data type: occurence
Brief description: Distribution of *Scatella bryani*. Samples with GPS coordinates shown in white; georeferenced historical samples shown in red.
File: oo_8579.kml PM O'Grady

Supplementary material 3 Distribution of Scatella cilipes Data type: occurence
Brief description: Distribution of *Scatella cilipes*. Samples with GPS coordinates shown in white; georeferenced historical samples shown in red.
File: oo_8580.kml PM O'Grady

Supplementary material 4 Distribution of Scatella cinereifacies Data type: occurence
Brief description: Distribution of *Scatella cinereifacies*. Samples with GPS coordinates shown in white; georeferenced historical samples shown in red.
File: oo_8581.kml PM O'Grady

Supplementary material 5 Distribution of Scatella clavipes
Data type: occurence
Brief description: Distribution of *Scatella clavipes*. Samples with GPS coordinates shown in white; georeferenced historical samples shown in red.
File: oo_8582.kml PM O'Grady

Supplementary material 6 Distribution of Scatella femoralis Data type: occurence
Brief description: Distribution of *Scatella femoralis*. Samples with GPS coordinates shown in white; georeferenced historical samples shown in red.
File: oo_8583.kml PM O'Grady

Supplementary material 7 Distribution of Scatella fluvialis Data type: occurence
Brief description: Distribution of *Scatella fluvialis*. Samples with GPS coordinates shown in white; georeferenced historical samples shown in red.
File: oo_8584.kml PM O'Grady

Supplementary material 8 Distribution of Scatella hawaiiensis Data type: occurence
Brief description: Distribution of *Scatella hawaiiensis*. Samples with GPS coordinates shown in white; georeferenced historical samples shown in red.
File: oo_8585.kml PM O'Grady

Supplementary material 9 Distribution of Scatella kauaiensis Data type: occurence
Brief description: Distribution of *Scatella kauaiensis*. Samples with GPS coordinates shown in white; georeferenced historical samples shown in red.
File: oo_8586.kml PM O'Grady

Supplementary material 10 Distribution of Scatella mauiensis Data type: occurence
Brief description: Distribution of *Scatella mauiensis*. Samples with GPS coordinates shown in white; georeferenced historical samples shown in red.
File: oo_8587.kml PM O'Grady

Supplementary material 11 Distribution of Scatella oahuense Data type: occurence
Brief description: Distribution of *Scatella oahuense*. Samples with GPS coordinates shown in white; georeferenced historical samples shown in red.
File: oo_8588.kml PM O'Grady

Supplementary material 12 Distribution of Scatella terryi Data type: occurence
Brief description: Distribution of *Scatella terryi*. Samples with GPS coordinates shown in white; georeferenced historical samples shown in red.
File: oo_8589.kml PM O'Grady

Supplementary material 13 Distribution of Scatella warreni Data type: occurence
Brief description: Distribution of *Scatella warreni*. Samples with GPS coordinates shown in white; georeferenced historical samples shown in red.
File: oo_8590.kml PM O'Grady

Supplementary material 14 Distribution of Scatella williamsi Data type: occurence
Brief description: Distribution of *Scatella williamsi*. Samples with GPS coordinates shown in white; georeferenced historical samples shown in red.
File: oo_8591.kml PM O'Grady

Supplementary material 15 Distribution of Scatella wirthi Data type: occurence
Brief description: Distribution of *Scatella wirthi*. Samples with GPS coordinates shown in white; georeferenced historical samples shown in red.
File: oo_8592.kml PM O'Grady

XML Treatment for Scatella amnica

XML Treatment for Scatella bryani

XML Treatment for Scatella cilipes

XML Treatment for Scatella cinereifacies

XML Treatment for Scatella clavipes

XML Treatment for Scatella femoralis

XML Treatment for Scatella fluvialis

XML Treatment for Scatella hawaiiensis

XML Treatment for Scatella kauaiensis

XML Treatment for Scatella mauiensis

XML Treatment for Scatella oahuense

XML Treatment for Scatella sexnotata

XML Treatment for Scatella stagnalis

XML Treatment for Scatella terryi

XML Treatment for Scatella warreni

XML Treatment for Scatella williamsi

XML Treatment for Scatella wirthi

## Figures and Tables

**Table 1.** **Distribution of *Scatella* species in Hawaii.** Status indicates whether the species are endemic (E), introduced (I) or adventive (A). Islands sampled are: Kauai (K), Oahu (O), Molokai (Mo), Lanai (L), Maui (Ma), and Hawaii (H). Presence of a species on a given island is designated by an X. An asterisk denotes a new island record for the species. There are two species with records in the Northwest Hawaiian Islands: *Scatella sexnotata* (1) has also been recorded from Nihoa, Necker and Laysan, *Scatella stagnalis* (2) is known from Midway.

Species	Status	K	O	Mo	L	Ma	H
*Scatella amnica*	E	X*		X		X	X
*Scatella bryani*	E	X	X	X	X	X	X
*Scatella cilipes*	E	X	X	X		X	X
*Scatella cinereifacies*	E	X					
*Scatella clavipes*	E	X	X	X		X	X
*Scatella femoralis*	E					X	
*Scatella fluvialis*	E		X				X
*Scatella hawaiiensis*	E	X	X	X	X	X	X
*Scatella kauaiensis*	E	X					
*Scatella mauiensis*	E			X		X	X
*Scatella oahuense*	E		X		X*	X	X
*Scatella sexnotata* ^1^	I	X	X	X		X	X
*Scatella stagnalis* ^2^	A	X*	X				X
*Scatella terryi*	E		X			X*	
*Scatella warreni*	E	X	X	X		X	X
*Scatella williamsi*	E						X
*Scatella wirthi*	E					X	X
